# Investigations on small molecule inhibitors targeting the histone H3K4 tri-methyllysine binding PHD-finger of JmjC histone demethylases

**DOI:** 10.1016/j.bmc.2018.03.030

**Published:** 2018-07-15

**Authors:** Bhaskar Bhushan, Alexandre Erdmann, Yijia Zhang, Roman Belle, Catrine Johannson, Udo Oppermann, Richard J. Hopkinson, Christopher J. Schofield, Akane Kawamura

**Affiliations:** a Department of Chemistry, University of Oxford, Mansfield Road, Oxford OX1 3TA, United Kingdom; b Radcliffe Department of Medicine, Division of Cardiovascular Medicine, University of Oxford, The Wellcome Trust Centre for Human Genetics, Roosevelt Drive, Oxford OX3 7BN, United Kingdom; c Botnar Research Centre, NIHR Oxford Biomedical Research Unit, University of Oxford, Oxford, United Kingdom

**Keywords:** Epigenetics, PHD-finger inhibitor, Plant Homeodomain, JmjC-KDMs, Histone demethylases

## Abstract

Plant homeodomain (PHD) containing proteins are important epigenetic regulators and are of interest as potential drug targets. Inspired by the amiodarone derivatives reported to inhibit the PHD finger 3 of KDM5A (KDM5A(PHD3)), a set of compounds were synthesised. Amiodarone and its derivatives were observed to weakly disrupt the interactions of a histone H3K4me3 peptide with KDM5A(PHD3). Selected amiodarone derivatives inhibited catalysis of KDM5A, but in a PHD-finger independent manner. Amiodarone derivatives also bind to H3K4me3-binding PHD-fingers from the KDM7 subfamily. Further work is required to develop potent and selective PHD finger inhibitors.

## Graphical abstract

**Figure:**
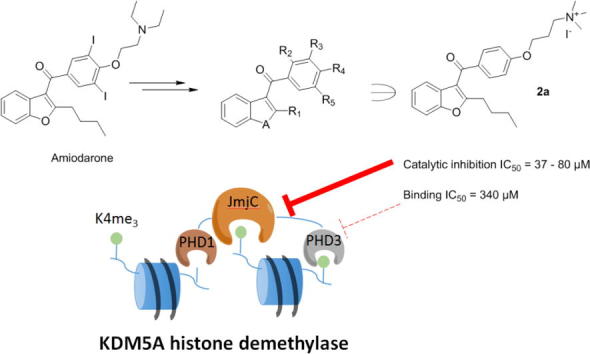

## 1 Introduction

Most human genes are subject to epigenetic regulation, including by the post-translational modifications of histones. These ‘epigenetic marks’ are recognised and maintained by a diverse set of regulatory proteins and enzymes.^1^ The maintenance of these marks is vital for the functioning and maintenance of cells, and their dysregulation is linked to multiple diseases, including cancer, cardiovascular disease, and developmental disorders.^2^, ^3^, ^4^, ^5^

PHD (plant homeodomain) fingers are C4HC3 type zinc-finger binding domains present in many chromatin-modifying proteins.^6^, ^7^ These small 50–100^33^ residueS domains bind to histones to enable the localisation of enzyme(s) to specific targets and promote the recruitment of transcription factors or chromatin-associated protein complexes.^7^ While the roles of many PHD-fingers are unclear, some PHD-fingers recognise specific histone modifications, including non-methylated or methylated lysines (e.g., histone H3 at K4 and K9), arginines (e.g., H3R2me1/me2), and acetylated lysines (e.g., H3K14).^7^, ^8^ PHD-fingers can also function as allosteric modulators of the activities of associated enzymes. Mutations, deletions or chromosomal translocations of PHD-finger encoding genes are linked to a range of diseases, including cancer, immunodeficiency and neurological disorders;^6^, ^7^ thus PHD-fingers are important epigenetic regulators.

**Fig. 1:**
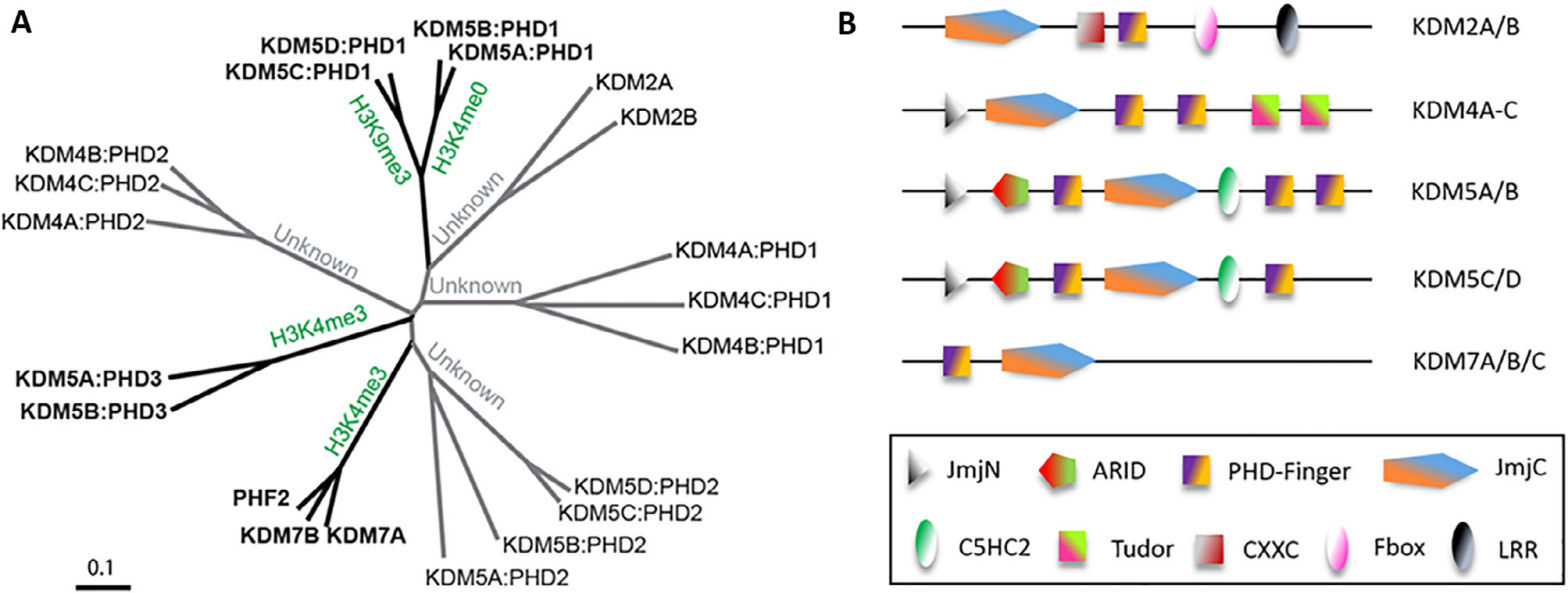
PHD-finger domains associated with the JmjC-KDMs. A) Phylogenetic tree of the PHD-finger domains in human JmjC-KDM family proteins. Branch lengths are indicated as a cladogram, and recognized histone marks in green. B) Domain architectures of selected JmjC-KDMs with PHD-fingers.

Histone modifying enzymes, such as in the Jumonji-C (JmjC) domain-containing histone lysine demethylases (JmjC-KDMs), sometimes contain multiple PHD-fingers (Fig. 1).^9^ The KDM5 subfamily of JmjC-KDMs (KDM5A-D) catalyses demethylation of the transcriptionally activating tri- and di-methylated lysine-4 mark on histone H3 (H3K4me3/2), and is generally associated with transcriptional repression.^10^, ^11^, ^12^, ^13^ The KDM5s are associated with development and progression of multiple cancers,^11^, ^14^ and can mediate cancer cell drug tolerance and maintain tumour-initiating cells.^15^, ^16^ KDM5A/B have three PHD-fingers (PHD1-3, numbered sequentially from the *N*-terminus), whereas KDM5C/D have two. The roles of KDM5 PHD-fingers are partially characterised: KDM5A/B(PHD3) binds to H3K4me3, with decreasing affinity for lower methylation states,^17^ whereas KDM5A/B(PHD1) recognizes H3K4me0^17^ (demethylation product), and is implicated in ‘allosteric’ activation of KDM5 catalysis.^18^ It is proposed that PHD3 of KDM5A/B directs the JmjC domain to the H3K4me3 site; PHD1 binds to the demethylated product H3K4me0 and activates the JmjC domain through a positive-feedback mechanism. This is thought to propagate the transcriptionally inactive state of chromatin by K4me3 removal along the H3K4me3-enriched promoters.^17^, ^18^ KDM5A(PHD3) is implicated in acute myeloid leukemia (AML) and forms a fusion protein with nucleoporin protein 98 (NUP98), a common translocation partner.^19^ This fused KDM5A(PHD3):NUP98 found in AML patients directs the “oncoprotein” to H3K4me3 promoter sites, inducing aberrant active transcription leading to AML, as shown in cellular and animal models.^20^ PHD3 mutations that disrupt H3K4me3 binding inhibit leukaemic transformation.^20^ In ER^-^ breast cancers, KDM5A promotes progression and metastasis, but its critical role in metastasis is apparently independent of its catalytic activity and regulated by the *N*-terminal PHD1/ARID domain regions.^21^ A point mutation (A388P) in KDM5C(PHD1) is linked to patients with X-linked mental retardation, and manifests reduced H3K4 demethylase activity.^13^ Overall, these results identify their PHD domains to be crucial to the KDM5 function(s) (both catalytic and non-catalytic), and in addition to JmjC-targeting,^22^, ^23^ suggest they are interesting targets for oncology.

By contrast to the catalytic domains of epigenetic proteins, (e.g., DNA methyltransferases, histone deacetylases, histone methyltransferases, demethylases and bromodomains),^24^, ^25^ chemical tools available for PHD-fingers are lacking and progress towards inhibitor development has been limited.^26^, ^27^ PHD-finger inhibitors will be useful in exploring their biological functions and therapeutic potential.

In 2012, Wagner et al. identified amiodarone (AMI), an antiarrhythmic drug, as an inhibitor of KDM5A(PHD3).^26^ Analogues of AMI (WAG-003, WAG-005) were reported to inhibit the binding of KDM5A(PHD3) to H3K4me3 with IC_50_ values of 30 ± 14 µM and 41 ± 16 µM, respectively, on the basis of a HaloTag-based peptide displacement assay, and supported by fluorescence polarisation assay results.^26^ While WAG-003 also inhibited other H3K4me3 binding domains (PHD in RAG and double tudor domain (DTD) in KDM4A), it showed modest selectivity over other tested PHD-fingers and Tudor domains (AIRE PHD1, BHC80 PHD, UHRF1(TDD)). However, the mode of action of AMI derivatives and their potential effect on KDM5A catalytic activity was unclear.

**Fig. 2:**
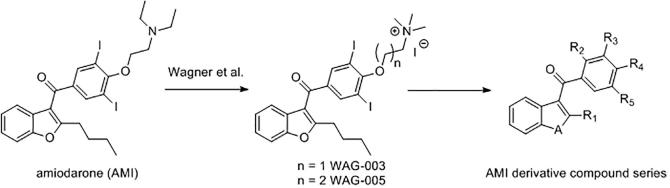
Design of potential PHD-finger binders from the structure of amiodarone.

We describe the synthesis of a series of AMI derivatives and structure-activity-relationship (SAR) studies on their KDM5A(PHD3) binding and H3K4me3 demethylation catalysis by KDM5A (Fig. 2). The results reveal that, while AMI and its derivatives bind weakly to PHD-fingers of KDM5A and other PHD-fingers within the JmjC-KDMs, they also inhibit the demethylation activity in a PHD-finger independent manner, suggesting AMI derivatives can act via more than one binding mode.

## 2 Results

### 2.1 Synthesis of AMI derivatives

**Scheme 1:**
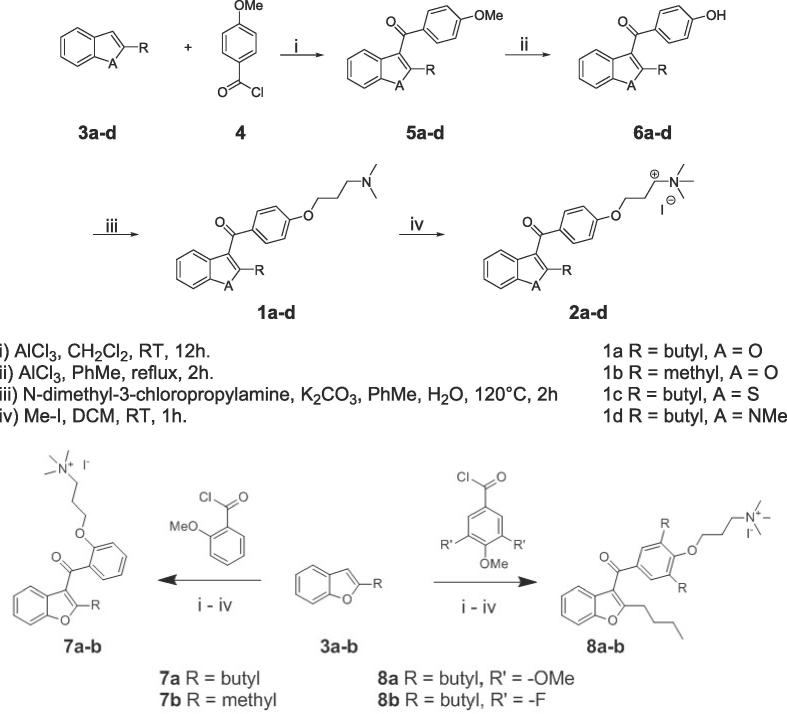
Synthesis of derivatives of **1a**.

The AMI derivatives were synthesised as in Scheme 1. To explore the importance of alkylation at the 2′-position of the benzofuran core, benzofuran derivatives **3** were reacted with 4-methoxybenzoyl chloride **4** using AlCl_3_ mediated Friedel-Crafts acylation to give **5a**–**d**. AlCl_3_-mediated demethylation yielded **6a**–**d**; alkylation of the phenol of these with *N*,*N*-dimethyl-3-chloropropylamine afforded dimethylated lysine analogues **1a**–**d**. Treatment with iodomethane gave the corresponding quaternary ammonium compounds **2a**–**d**. For diversification at the aryl portion of AMI, **3a** and **3b** (Scheme 1**b**) were employed. 3,4,5-Trimethoxybenzoyl chloride and 3,5-difluoro-4-methoxybenzoyl chloride were used to prepare **8a** and **8b** respectively. 2-Methoxybenzoyl chloride was used to prepare **7a** and **7b**, the regioisomers of **2a** and **2b**, respectively, to investigate the effect of altering the position of the 3-(trimethylammino)-propoxy group from the *para*- to the *ortho*-position.

**Scheme 2:**
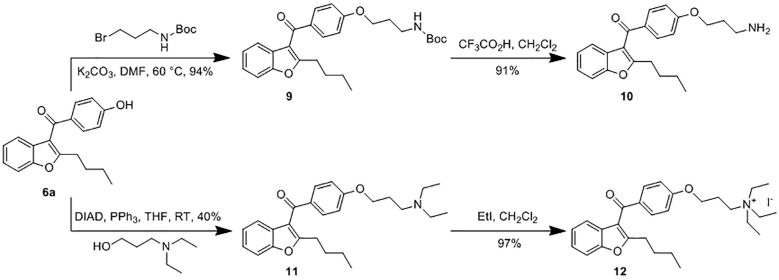
Modification of the quaternary ammonium group of potential PHD inhibitors.

To explore the importance of the quaternary ammonium of histone lysines for binding to KDM5A(PHD3), different alkylated amines of AMI were prepared, starting from **6a** (Scheme 2). Thus, alkylation of **6a** gave **9**, which was deprotected to give primary amine **10**. Mitsunobu reaction of **6a** with *N*,*N*-diethyl-3-aminopropanol gave **11** in moderate yield, which was converted to **12** by reaction with iodoethane.^34^

### 2.2 Development of KDM5A(PHD3) AlphaScreen binding assay

**Fig. 3:**
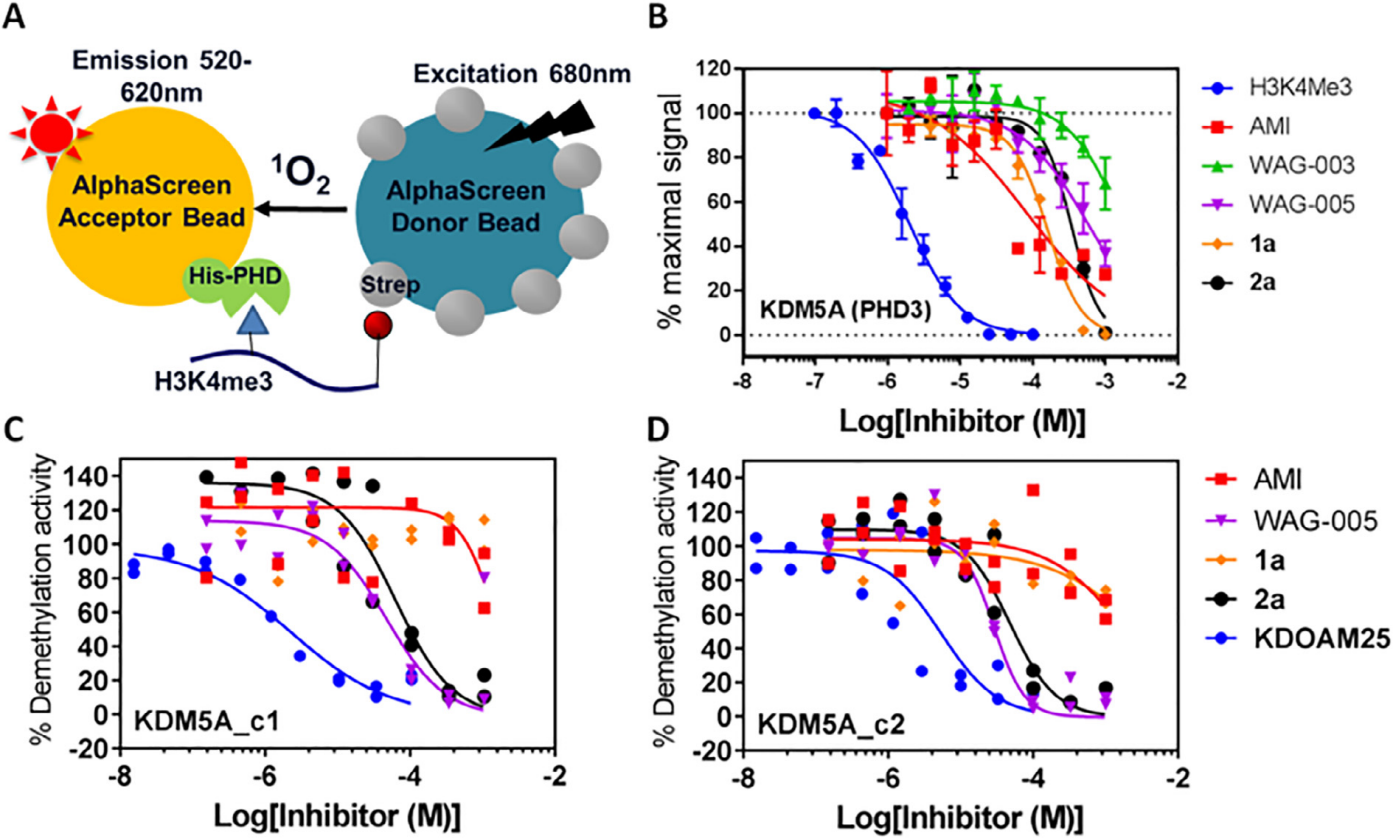
Screening of AMI analogues for binding and catalytic inhibition of KDM5A. (A) AlphaScreen assay for H3K4me3 and His-KDM5A(PHD3) interactions. (B) Normalised dose–response inhibition curves for displacement of H3K4me3-Bn from KDM5A(PHD3) by representative AMI derivatives. Average ± StdDev (N ≥ 3 independent replicates). **(C, D)** Dose-response inhibition curves of H3K4me3 demethylation activity by AMI derivatives for KDM5A using a MALDI-TOF MS-based assay. AMI derivatives were tested against two active KDM5A constructs, KDM5A_c1 (M1-L801), and KDM5A_c2 (ΔARID/PHD1, L88-G353). KDOAM25a is a small molecule JmjC-domain inhibitor of KDM5.

To assess whether the AMI derivatives inhibit binding of histone H3K4me3 peptide to KDM5A(PHD3), we developed an AlphaScreen displacement assay. AlphaScreen is a homogenous bead-based assay used to study protein-protein interactions.^28^ We used a streptavidin-conjugated donor and nickel-conjugated acceptor bead pair to detect the interactions of His_6_-tagged KDM5A(PHD3) (His-KDM5A(PHD3)) and C-terminally biotinylated-H3(1–21)K4me3 (H3K4me3-Bn) (Fig. 3A). Optimised assay conditions were determined to ensure that the His-KDM5A(PHD3) and H3K4me3-Bn interactions could be detected in the linear range of the assay, with good signal-to-background ratio (Supplementary Fig. S1). To investigate the inhibition of the protein-peptide interaction, His-KDM5A(PHD3) was pre-incubated with compounds (15 min), followed by incubation with H3K4me3-biotin (30 min). AlphaScreen beads were then added and incubated for 1 h. Changes in the AlphaScreen signals with increasing inhibitor concentrations were then measured to quantify the level of KDM5A(PHD3) and H3K4me3 binding, and IC_50_ values were determined. Using this setup, IC_50_ values for displacement of biotinylated H3K4me3 by non-biotinylated histone peptides were seen to decrease with increasing methylation state at H3K4 (Kme3 < Kme2 < Kme1 < Kme0) (Supplementary Fig. S2) confirming the rank order of peptide affinities to KDM5A(PHD3), as previously reported based on isothermal titration calorimetry.^20^ Following assay optimisation, the set of AMI derivatives was tested for KDM5A (PHD3) inhibitory activity (Table 1, Fig. 3B, Supplementary Fig. S3).

**Table 1:** AMI derivatives (Fig. 2) and associated Alphascreen IC_50_ values for displacement of H3K4me3-Bn from His-KDM5A(PHD3). Average ± StdDev (n ≥ 3 independent replicates) shown. H3(1–21)K4me3 IC50: 218 ± 16 nM.

Compounds	A	R_1_	R_2_	R_3_ = R_5_	R_4_	IC_50_ (µM)	Biotin-His_6_ counterscreen
**Amiodarone** **(AMI)**	O	butyl	H	I		99 ± 23	178 ± 58
**WAG-003**	O	butyl	H	I		1658 ± 490	11280 ± 1258
**WAG-005**	O	butyl	H	I		562 ± 130	2584 ± 127
**1a**	O	butyl	H	H		166 ± 20	484 ± 82
**2a**	O	butyl	H	H		340 ± 44	4297 ± 163
**2b**	O	methyl	H	H		844 ± 23	6291 ± 1865
**2c**	S	methyl	H	H		533 ± 109	805 ± 55
**2d**	NMe	H	H	H		1310 ± 39	970 ± 60
**7a**	O	butyl		H	H	1146 ± 100	DNC^a^
**7b**	O	methyl		H	H	2305 ± 82	DNC^a^
**8a**	O	butyl	H	OMe		223 ± 60	309 ± 70
**8b**	O	butyl	H	F		198 ± 50	455 ± 57
**9**	O	butyl	H	H		NM^b^	NM^b^
**10**	O	butyl	H	H		198 ± 41	376 ± 69
**12**	O	butyl	H	H		314 ± 30	1335 ± 326

a DNC: data did not converge to binding model.

b NM: not measurable within the solubility range of the compound. Final concentration of protein and peptide were 25 nM.

Interestingly, although the AlphaScreen-based peptide displacement assay works on similar principles to the HaloTag assay reported by Wagner et al.,^26^ IC_50_ values measured against KDM5A(PHD3) differed for the set of AMI derivatives in our assays. Whilst WAG-005 and WAG-003 are reported to have IC_50_ values of 30–40 µM using the Halo-tag assay, they had >10-fold (562 µM) and >50-fold higher IC_50_ values (1658 µM), respectively, in our assay. While AMI had an IC_50_ value of 99 µM, non-specific interference of AMI (and some derivatives) with the AlphaScreen assay in the counter screen using biotin-His_6_ was observed (Table 1). Taking this into account, SAR analysis of further AMI variants on KDM5A(PHD3) indicates that removal of the 3,5-di-iodo substitutions from the WAG-005 aryl group increased potency (**2a**, IC_50_ = 340 µM); alternative substitutions (3,5-dimethoxy (**8a**), 3,5-difluoro (**8b**)) were also favourable (198 µM, 223 µM respectively). The results reveal that *p*-substitution (**2a**) is favoured over *o*-substitution (**7a**) for the 3-(trimethylammino)-propoxy group. Alkylation of amines was then explored (**2a**, **7a**, **10**, **12**). The tertiary amine (di-methyl (**1a**)) was the most potent inhibitor with an IC_50_ value of 166 µM, followed by the primary amine (**10**), with the quaternary amine (tri-methyl- (**2a**), tri-ethyl (**12**)) being least potent. Reducing the length of the alkyl chain at R1 from butyl (**2a**) to methyl led to a reduction in activity (**2b**). The benzophenone **2c** was more potent than **2b** for KDM5A(PHD3), whilst the *N*-methylindole analogue (**2d**) had reduced activity. These studies suggest that further exploration and diversification of the heterocyclic rings is of interest. Overall, the most potent inhibitor in this panel against KDM5A(PHD3) was **1a** with an IC_50_ of 166 µM.

### 2.3 Inhibition of KDM5A demethylation activity by AMI derivatives

We next investigated the effect of AMI derivatives on the demethylation activity of KDM5A. Two catalytically active constructs of KDM5A were employed, KDM5A_c1 (M1-L801) and KDM5A_c2 (ΔARID/PHD1, P13-S744 with L88-G353 (ARID/PHD1) replaced with a GGGG linker) and both KDM5A_c1 and KDM5A_c2 constructs lack the PHD2 and PHD3. We thus proposed that KDM5A catalytic activity should not be affected by AMI derivatives since the PHD3 domain in these proteins is absent. A MALDI-TOF MS assay was used to measure the activity of H3K4me3 demethylation in the presence of inhibitors (Table 2, Fig. 3C and D). KDOAM25, a KDM5-selective JmjC-domain targeting small molecule inhibitor,^29^ was used as a positive control. Interestingly, while AMI was not an inhibitor of KDM5A activity, WAG-005 and **2a** inhibited demethylation activity of both KDM5A_c1 and KDM5A_c2, at concentrations significantly below the AlphaScreen displacement IC_50_ values with isolated PHD3 (Table 2, Supplementary Fig. S6).

**Table 2:** Inhibition of H3K4me3 demethylation activity of KDM5A using MALDI-TOF MS assays. Dose-response inhibition assays were carried out with AMI derivatives or KDOAM25 using different constructs of KDM5A. Average ± StdDev of *n* = 2 independent assays.

Compounds	IC_50_ (µM)	
	KDM5A_c1	KDM5A_c2
**AMI**	>1000	>1000
**WAG-005**	82 ± 54	27 ± 2
**1a**	>1000	>1000
**2a**	80 ± 29	37 ± 14
**KDOAM25**	1.7 ± 0.6	3.2 ± 3.1

This suggests that both WAG-005 and **2a** can inhibit the catalytic activity of KDM5A in a manner independent of PHD-finger binding, presumably via binding to the JmjC domain. Further kinetic analysis of KDM5A_c2 using formaldehyde dehydrogenase (FDH) enzyme-coupled fluorescence assay indicated that **2a** binding is unlikely to be competitive with respect to H3K4me3 binding (α = 1.64 ± 0.85, *K_i_* = 74 ± 28 µM) (Supplementary Fig. S6A, B). No formaldehyde production was detected with **2a** in the absence of H3K4me3, suggesting that **2a** is not a substrate of KDM5A_c2, at least, under the assay conditions tested (Supplementary Fig. S6C).

### 2.4 Inhibition of H3K4me3-binding PHD-fingers of other JmjC-KDMs by AMI derivatives

In order to test the specificity of the AMI derivatives for KDM5A, cross-screens were performed using AlphaScreen binding assays against the H3K4me3-binding PHD-fingers associated with other JmjC-KDMs: KDM7A(KIAA1718), KDM7B(PHF8) and KDM7C(PHF2) (Fig. 1A, Supplementary Figs. S4, S5).^30^ The PHD-fingers of the KDM7 subfamily recognise H3K4me3.^30^, ^31^ In the case of KDM7B, the PHD-finger primes the JmjC domain to catalyse demethylation of nearby H3K9me2 (Fig. 1). Conversely, in KDM7A, binding of H3K4me3 to the PHD-finger negatively affects the demethylation activity of the JmjC domain at the H3K9me2 site, likely due to the spacing constrains between the two domains.^30^

**Fig. 4:**
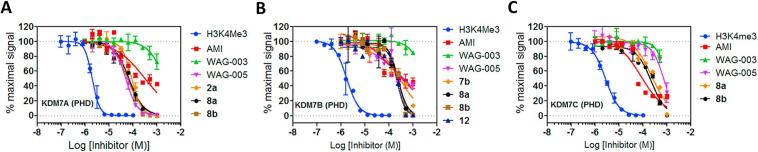
AlphaScreen binding assay for H3K4me3 and His-KDM7(PHD). Normalised dose–response inhibition curves for the displacement of H3K4me3-Bn from KDM7A/B/C by representative AMI derivatives.

The majority of AMI derivatives weakly inhibited histone peptide binding by the KDM7 PHD-fingers, albeit to varying degrees (Table 3, Fig. 4). As observed with KDM5A(PHD3), there was little consensus in terms of the preferred methylation and substitution state of the amine group, with the trimethylammonium group containing WAG-005 being most effective against KDM7A(PHD), the triethylamino group containing compound **12** being most effective against KDM7B(PHD), and the dimethylamino group containing **1a** being most effective against KDM7C(PHD). This was somewhat surprising, given the ∼88% sequence similarity between the KDM7 PHD-fingers. Additionally, there was a preference for the 3-(trimethylamine)-propoxy group in WAG-005 over the 2-(trimethylamino)-ethoxy group in WAG-003 for binding to KDM7A(PHD).

**Table 3:** AlphaScreen IC_50_ values for displacement of H3K4me3-Bn from His-KDM7(PHD) by AMI derivatives. Final concentrations of protein and H3K4me3-Bn were: 6.25 nM KDM7A(PHD) and 25 nM peptide, 12.5 nM each for KDM7B(PHD), and 25 nM KDM7C(PHD) and 6.25 nM peptide. Average ± StdDev (n ≥ 3 independent replicates) shown.

Compounds	IC_50_ (µM)		
	KDM7A(PHD)	KDM7B(PHD)	KDM7C(PHD)
**AMI**	156 ± 12	252 ± 53	84 ± 14
**WAG-003**	2232 ± 790	307 ± 76	976 ± 146
**WAG-005**	50 ± 5	390 ± 158	740 ± 149
**1a**	333 ± 39	397 ± 77	158 ± 30
**2a**	86 ± 9	355 ± 39	579 ± 87
**2b**	108 ± 15	310 ± 78	598 ± 119
**2c**	104 ± 8	377 ± 76	501 ± 75
**2d**	98 ± 10	697 ± 154	682 ± 97
**7a**	102 ± 15	496 ± 181	1294 ± 280
**7b**	326 ± 14	286 ± 84	533 ± 99
**8a**	76 ± 14	240 ± 58	228 ± 66
**8b**	68 ± 13	242 ± 41	220 ± 40
**10**	534 ± 150	386 ± 41	359 ± 89
**12**	151 ± 17	221 ± 57	334 ± 45
**H3(1–21)K4me3**	1.9 ± 0.1 μM	1.7 ± 0.2 μM	2.7 ± 0.2 μM

As observed for the KDM5A(PHD3), all AMI derivatives with *o*-substitutions (**7a**) were less potent than those with *p*-substitutions (**2a**); the 3,5-dimethoxy- and difluoro-containing compounds **8a** and **8b** were more potent than their unsubstituted analogue **2a**. There was less agreement in the effect of the removal of the 3,5-diiodo-groups, which reduced potency against KDM7A(PHD), and improved potency against KDM7B(PHD) and KDM7C(PHD) (Table 3, Fig. 4). Divergent trends are also observed in the heterocyclic region (**2b**, **2c**, and **2d)**; *N*-methylindole **2d** was most potent against KDM7A(PHD), benzofuran **2b** was most potent against the KDM7B(PHD), and the benzothiophene-containing **2c** was most potent against KDM7C(PHD). Overall, the results reveal that AMI derivatives inhibit the KDM7 PHD-fingers, indicating that they are likely not selective. WAG-003 and WAG-005 inhibited the PHD-fingers of KDM7s at different potencies, with WAG-005 being the most potent inhibitor of KDM7A(PHD).

### 2.5 Inhibition of binding and catalytic activity of KDM7A/B(PHD-JmjC) by AMI derivatives

We next explored the effect of AMI and select derivatives against the KDM7A/B JmjC domains. A dual-domain construct of KDM7A/B(PHD-JmjC), consisting of a JmjC domain in addition to the PHD-finger (Fig. 1B), was used to test for allosteric inhibition through binding at the PHD-finger. The displacement IC_50_ profiles for the dual domains were significantly different, and generally lower, compared to that of the PHD finger-only constructs (Table 4). KDM7A(PHD-JmjC) was particularly sensitive, with IC_50_ values of WAG-005 and **2b** at 16 µM and 5.6 µM, respectively.

**Table 4:** Alphascreen IC_50_ values for displacement of H3K4me3-Bn from His-KDM7(PHD-JmjC) by AMI derivatives, and their inhibition of demethylation activity assessed by MALDI-TOF MS. Displacement – AlphaScreen displacement data. Catalytic – H3K9me2 demethylation assay using MALDI-TOF MS. NI: No inhibition seen at 100 μM. Average ± StdDev (n = 3 for AlphaScreen).

Compounds	IC_50_ (µM)			
	KDM7A (PHD-JmjC) (Displacement)	KDM7A (PHD-JmjC) (Catalytic)	KDM7B (PHD-JmjC) (Displacement)	KDM7B (PHD-JmjC) (Catalytic)
**AMI**	80 ± 26	83% (100 µM)	–	22% (100 µM)
**WAG-003**	NI	17% (100 µM)	NI	22% (100 µM)
**WAG-005**	16 ± 4	100% (100 µM)	NI	33% (100 µM)
**1a**	295 ± 48	100% (100 µM)	NI	95% (100 µM)
**2a**	149 ± 32	100% (100 µM)	120 ± 32	89% (100 µM)
**2b**	5.6 ± 1.5	100% (100 µM)	126 ± 34	32% (100 µM)
**2d**	88 ± 17	100% (100 µM)	>300	24% (100 µM)

The AMI derivatives were then tested for inhibition of KDM7A/B-catalysed H3K9me2 demethylation using the MALDI-TOF MS assay. H3(1–15)K9me2 and H3(1–5)K4me3K9me2 were used as substrates for KDM7A(PHD-JmjC) and KDM7B(PHD-JmjC), respectively. For KDM7A(PHD-JmjC), complete inhibition of K9me2 demethylation activity was observed for all AMI derivatives tested (except WAG-003) at 100 µM. For KDM7B(PHD-JmjC), inhibition was most pronounced for **1a** (95%) and **2a** (89%) whilst others inhibited more weakly (Table 4).

## 3 Discussion

AMI and its derivatives (WAG-003, WAG-005) have been identified as KDM5A(PHD3) inhibitors;^26^ they remain the only reported inhibitors of PHD-fingers linked to JmjC-KDMs. To investigate the tractability and selectivity of PHD-finger inhibition by this scaffold, we generated a series of AMI derivatives and assessed their SAR against KDM5A(PHD3) using an AlphaScreen-based binding assay. While some of the AMI derivatives did indeed weakly displace the H3K4me3 binding to KDM5A(PHD3), our results suggest their potencies are an order of a magnitude lower than previously reported. Small improvements in potency were obtained by modifying the 3,5-substitutions on the aryl group. We hypothesised that the tertiary amine in WAG-005 may be binding at the *N*^ε^-methylated lysine binding pocket of KDM5A(PHD3). However, no clear differences in binding potency was observed for derivatives with differing amine alkylation states, in agreement with previous findings.^26^ This is in contrast to histone H3 peptides, where higher methylation states are clearly favoured (as shown for KDM5A in Supplementary Fig. S2).

Interestingly, some AMI derivatives inhibited the catalytic activity of the KDM5A and KDM7A/B JmjC domain, further indicating that they may act/bind via more than one mode. Kinetic analysis on **2a** suggests that the binding is not competitive with respect to histone H3K4me3 (Supplementary Fig. S6). However, further biophysical analyses are needed to elucidate the precise modes of action of the compounds.

The AMI derivatives were also found to be promiscuous binders of the KDM7 PHD-fingers, and there was no clear trend in their binding profiles across the PHD-fingers. It is important to note that IC_50_ values are dependent on assay conditions. The differences between the assay setup, such as the length of peptide and protein constructs, can affect the absolute IC_50_ values (H3(1–21)K4me3 and KDM5A_1542–1660_ (PHD3) were used in our study compared to H3(1–11 or 1–14)K4me3 and KDM5A_1601-1660_ (PHD3) used by Wagner et al.^26^). It is possible that the increased binding affinity of the longer peptide ligand to the longer reader domain scaffold in our study may contribute to the higher IC_50_ values obtained. Indeed, it is well reported that the binding and catalytic activities change with different protein construct length and domain architecture,^32^ as demonstrated for the KDM5A and the KDM7 subfamilies in this study. Thus, while isolated protein domains and peptide fragments provide useful information, more work with full-length proteins and nucleosomes are likely needed for robust evaluation of small-molecule interactions.

The results presented here imply that AMI derivatives need further optimisation to be useful as probes for the PHD-fingers. More generally, they highlight the need for good quality PHD-finger inhibitors. Despite the emerging importance of the PHD-fingers in driving epigenetic changes in diseases and compelling biology to support PHD-finger as attractive cancer targets, the PHD-fingers have been considered difficult targets for small molecule inhibition. Our results support this view, highlighting the challenges in developing selective and potent small molecule inhibitors against the PHD-fingers.
