# Supplementary material for: Investigations on small molecule inhibitors targeting the histone H3K4 tri-methyllysine binding PHD-finger of JmjC histone demethylases
Source: Bioorg Med Chem. 2018 Jul 15;26(11):2984–91. doi: 10.1016/j.bmc.2018.03.030 (PMC6380468; doi:10.1016/j.bmc.2018.03.030)
Supplement: Supplementary data 2 [file mmc2.docx]

Supplementary Information

**Investigations on Small Molecule Inhibitors Targeting the Histone H3K4 Tri-methyllysine Binding PHD-finger of JmjC Histone Demethylases**

Bhaskar Bhushan,^1,2#^ Alexandre Erdmann,^1#^ Yijia Zhang,^1^ Roman Belle,^1^ Catrine Johannson,^1^ Udo Oppermann,^3^ Richard J Hopkinson,^1+^ Christopher J Schofield^1^ and Akane Kawamura^1,2^*

^1^Department of Chemistry, University of Oxford, Mansfield Road, Oxford OX1 3TA, United Kingdom.

^2^Radcliffe Department of Medicine, Division of Cardiovascular Medicine, The Wellcome Trust Centre for Human Genetics, Roosevelt Drive, Oxford OX3 7BN, United Kingdom

^3^Botnar Research Centre, NIHR Oxford Biomedical Research Unit, University of Oxford, Oxford, United Kingdom.

^+^Current address: Leicester Institute of Structural and Chemical Biology and Department of Chemistry, University of Leicester, Leicester, LE1 7RH, United Kingdom.

^#^These authors contributed equally to this work.

*Corresponding: Dr Akane Kawamura, akane.kawamura@chem.ox.ac.uk

**A B**

**Supplementary Figure S1.** **AlphaScreen binding assay optimisation for KDM5A(PHD3).** (A) The linear range for the interaction of His-KDM5A(PHD3) with H3K4me3-Bn was determined. Data for the H3K4me3-Bn peptide gradient (0-400 nM) is shown. (B) In the final assay, 25 nM protein and 25 nM peptide were used, as indicated by a red star.

**Supplementary Figure S2. Dose-response inhibition curves for non-biotinylated H3K4me0/1/2/3 peptides.** The interaction of His-KDM5A(PHD3) with H3K4me3-Bn was competed by histone H3 peptides with different methylation states at the H3K4 site. Observed IC_50_ values are : 571 ± 36 µM (Me0), 12.6 ± 2.5 µM (Me1), 1577 ± 140 nM (Me2), 218 ± 16 nM (Me3)

**Supplementary Figure S3.** Dose-response inhibition curves for the displacement of H3K4me3-Bn from KDM5A(PHD3) by a panel of AMI derivatives. See **Table 1** for the IC_50_ values.

**A B**

**C D**

**E F**

**Supplementary Figure S4.** **AlphaScreen binding assay optimisation for KDM7(PHD).** The linear range for the interaction of **(A)**His-KDM7A(PHD); **(C)**His-KDM7B(PHD); **(E)** His-KDM7C(PHD) with H3K4me3-Bn were determined. Data for the H3K4me3-Bn peptide gradient (0-200 nM) is shown. The final assay conditions are indicated by an arrow.

**A B**

**C D**

**E F**

**Supplementary Figure S5.** **Dose-response inhibition curves for the displacement of H3K4me3-Bn from KDM7(PHD) by a panel of AMI derivatives.** See **Table 3** for the IC_50_ values. (A, B) KDM7A(PHD) ; (C, D) KDM7B(PHD) ; (E, F) KDM7C(PHD).

**A B**

**C**

**Supplementary Figure S6.** **Michaelis-Menten (A) and Lineweaver-Burke (B) plots for the inhibition of KDM5_c2 activity by different concentrations of compound 2a with respect to histone H3K4me3 substrate. (A)** Michaelis Menten kinetic plots of KDM5A_c2 in the presence of **2a (**0 – 80 µM) were globally fitted to a mixed inhibition model (*K_i_* = 74 ± 28 µM, α = 1.64 ± 0.85). (B) Corresponding Lineweaver-Burk plots suggest **2a** is non-competitive inhibitor of KDM5A-c2 with respect to H3K4me3. (C) Formaldehyde production is only observed in the presence of H3(1-21)K4me3 at different concentrations of **2a**, suggesting that demethylation of **2a** does not take place under the conditions tested. All data are average ± StdDev (N=2, independent assays).

**I. Biological methods:**

Histone H3(1-21)Kme3/2/1/0 peptides were from GL Biochem (Shanghai) Ltd. The *C*-terminally biotinylated histone H3K4me3-Bn peptide was from Anaspec. KDOAM25, β-NAD^+^ and FDH were supplied from Sigma-Aldrich.

**Recombinant protein production**

**Recombinant production of PHD finger proteins**: KDM5A(PHD3) (1542 – 1660), encoded by a pGTLV2 vector, was obtained from the Structural Genomics Consortium, University of Oxford. The plasmid was transformed into Rosetta^TM^ 2(DE3)pLysS competent cells, and plated out onto LB-agar plates supplemented with kanamycin and chloramphenicol. A single colony was used to inoculate 20 mL of Terrific Broth medium supplemented with kanamycin and chloramphenicol, and incubated at 37 °C for 16h with shaking. The starter culture was then used to inoculate 2L of TB medium supplemented with kanamycin, and incubated at 37 °C with shaking until optical density (OD_600_) of 0.7-0.8 was obtained. The culture was further incubated for 1 h at 18 °C and induced with 0.2 mM isopropyl-β-D-thiogalactopyranoside (IPTG) for 18 hrs. The cells were pelleted by centrifugation, resuspended in lysis buffer (50 mM HEPES pH 7.5, 0.5 M NaCl, 10 mM imidazole, 5% glycerol, 1mM tris(2-carboxyethyl)phosphine (TCEP), supplemented with DNaseI and EDTA-free Protease Inhibitor Cocktail (Roche) and lysed by sonication. The cell debris was removed by centrifugation (12,000 rpm, 15 min, 4°C), and the protein was purified by incubation of the cleared lysate with 2 mL of Ni^2+^ charged IMAC Sepharose 6 Flast Flow resin. The resin was packed into a glass column and washed with lysis buffer followed by the same buffer containing 40 mM imidazole. The bound protein was then eluted with elution buffer (50 mM HEPES pH 7.5, 0.5 M NaCl, 5% glycerol, 250 mM imidazole, 0.5mM TCEP, 2 x 5mL).

The protein was concentrated to < 2mL using an Amicon Ultra-15 centrifugal concentrator (3000 Da MWCO, Merck Millipore Ltd), filtered through a 0.2 μm membrane, and injected onto a HiLoad 16/600 Superdex 200 PG gel filtration column (GE Life Sciences) for size-exclusion chromatography. The eluted protein was analysed by SDS-PAGE (NuPAGE 4-12% Bis-Tris gel, Invitrogen, MES buffer, 200 V, 35 min), and monomer-containing fractions were concentrated, aliquoted, flash frozen in liquid nitrogen, and stored at -80 °C.

The KDM7B PHD-finger and JmjC- dual-domain protein was expressed and purified as described previously.^1^ The detailed expression conditions for the KDM7A/B/C PHD-fingers, as well as the KDM7A dual-domain constructs will be reported elsewhere. KDM5A_c1 (1-801) and KDM5-c2 (13-744, Δ(88-353) and replaced by GGGG) were expressed and purified as described.^2^

**AlphaScreen Assays**

His-tagged PHD finger constructs and H3K4me3-Bn peptides were diluted into AlphaScreen assay buffer (50mM HEPES, 150mM NaCl, 0.1% BSA, 0.01% Tween20, pH 7.5). An 8-point 1:2 dilution series was made for both protein and peptide. With a multichannel pipette (LTS P10), 5 µL protein was dispensed into 8 columns of a 384-well plate, followed by 5 µL of 4% DMSO in AlphaScreen assay buffer. The mixture was incubated at room temperature for 15 minutes, followed by dispensing 5 µL peptide across 8 rows of the 384-well plate, forming a cross-gradient of protein and peptide. The contents were incubated for 30 minutes at room temperature, followed by the addition of 5 µL of a mixture of AlphaScreen Streptavidin donor beads and Nickel Chelate acceptor beads in AlphaScreen assay buffer (20 µg/mL final). The samples were incubated for 60-120 minutes at room temperature in the dark, followed by measurement of luminescence on a BMG Labtech Pherastar plate reader (Software Ver. 5.30 R3, Firmware Ver. 1.21). The data was plotted in Prism Ver 7.01 (GraphPad Software, Inc.) and fitted to a linear model in order to determine the optimal concentration of protein and peptide within the linear signal range for each reader domain.

For peptide displacement assays, 5 µL of the inhibitor dilution series (at a concentration of 4% DMSO in AlphaScreen assay buffer) was pre-incubated with 5 µL of the protein at RT for 15 minutes. Peptide solution (5 µL) was added and incubated for 30 minutes at RT, followed by the addition of 5 µL of a mixture of AlphaScreen Streptavidin donor beads and Nickel Chelate acceptor beads in AlphaScreen assay buffer (20 µg/mL final). The contents were incubated for 60-120 minutes at RT in the dark, followed by measurement of luminescence on a BMG Labtech Pherastar plate reader (Software Ver. 5.30 R3, Firmware Ver. 1.21). The data was plotted in Prism Ver 7.01 (GraphPad Software, Inc.) and fitted to a Log(inhibitor) v/s response – variable slope model in order to obtain IC_50_ values.

**Matrix-assisted laser desorption/ionisation time-of-flight mass spectrometry (MALDI-TOF) MS conditions**

The KDM5A mixture (5 μL, KDM5A-c1: 1.0 μM or KDM5A-c2: 0.5 μM) was pre-incubated with 100 nL inhibitors (in DMSO) for 10 min. Peptide mixture (5 μL) containing H3_1-21_K4me_3_ peptide (8.0 μM) sodium ascorbate (2.0 mM), (NH_4_)_2_Fe(II)(SO_4_)_2_ (100 μM) and 2-oxoglutarate (2OG, 400 μM) in assay buffer (HEPES (50 mM), NaCl (50 mM) in MQ, pH 7.5) was added to start the reaction. The mixture was quenched using formic acid (5 μL, 1 v/v% in MQ) after 4 minutes (KDM5A-c1) or 3 minutes (KDM5A-c2). The stopped reaction (1 μL) was mixed with 4-cyano-4-hydroxycinnamic acid (CHCA) MALDI matrix (saturated solution in TFA (0.1%) in MilliQ water and trifluoroacetic acid (0.1%) acetonitrile (1:1)) at 1:1 ratio (2 μL), air dried and analysed using Bruker microflex MALDI-TOF controlled with FlexControl. The data were further processed using FlexAnalysis and excel.

For KDM7(PHD-JmjC) assays, 2.5 μM enzyme was used, with 5 μM H3(1-15)K9me2 peptide substrate for KDM7A or H3(1-15)K4me3K9me2 for KDM7B. Final concentrations were: 1mM ascorbate, 50 μM (NH_4_)_2_Fe(II)(SO_4_)_2_ and 200 μM 2OG. The mixture was quenched using formic acid after 10 minutes (KDM7A) or 5 minutes (KDM7B).

**Formaldehyde dehydrogenase coupled activity assay**^2^

Enzyme mixture (25 μL) containing KDM5A-c2 (0.5 μM) and FDH (2.0 μM) in FDH buffer [50mM HEPES (50 mM), Tween20 (0.1%), NaCl (50 mM), pH 7.5 in MQ)] was prepared and aliquoted in a 384-well flat-bottom microplate (µClear, Greiner). The peptide mixture (25 μL) [ascorbate (2 mM), (NH_4_)_2_Fe(II)(SO_4_)_2_ (100 μM), 2OG (400 μM), H3(1-21)K4me_3_ peptide (ranging from 0 to 100 μM), β-NAD (500 μM), **2a** (0 to 160 μM, in DMSO (2% final in this solution)) in FDH buffer] was added to initiate the reaction and the fluorescence was monitored (every 30 seconds over 25 min at 25.0 °C) using the PHERAstar (BMG LABTECH). The initial rates at each peptide concentrations were used to generate the Michaelis-Menten plots. Combined datasets from two independent experiments were globally fitted to a mixed inhibition model using Prism Ver 7.01, and *K*_i_ and α values were determined. Lineweaver-Burk plots were also generated using Prism Ver 7.01

**II. Synthesis**

**General methods**

All solvents and chemicals were purchased from Sigma unless otherwise stated, and were used as supplied (analytical or HPLC grade), without prior purification. Milli-Q ultrapure water was used for chemical reactions and protein manipulations.

Proton nuclear magnetic resonance (^1^H NMR) spectra were recorded using a Bruker AVIIIHD 400 (400 MHz) machine. Carbon nuclear magnetic resonance (13C NMR) spectra were recorded on a Bruker AVIIIHD 400 (100 MHz) spectrometer. J values are reported to the nearest 0.5 Hz. The centroids of multiplet (m) resonances are reported.

Accurate mass analyses were performed using a Thermo Exactive mass spectrometer equipped with Waters Acquity liquid chromatography system. Instrument control and data processing were performed using Thermo Xcalibur Software. The system was calibrated on the day of the analysis and its mass accuracy with external calibration (as used for these experiments) is better than 5ppm for 24 hours following calibration. The mass spectrometer was operated using the heated electrospray (HESI-II) probe and resolution was set to 50,000. Electrospray source conditions were adjusted to maximise sensitivity. A mixture of 10% water, 89.9% methanol and 0.1% formic acid was used to transport samples to the mass spectrometer at a flow rate of 0.2 mL/min.

Infrared (IR) spectra were recorded on a Bruker Tensor 27 Fourier Transform spectrophotometer. Absorption maxima (υ_max_) are reported in wavenumbers (cm^–1^)

**General synthetic procedure A:** To a solution of the phenol derivative in 4 mL of toluene and 2 mL of water was added K_2_CO_3_ and the mixture was heated at 60°C under stirring. The dimethylamine derivative was then added portionwise and the temperature was increased until 120°C for 2 hours. The consumption of the starting phenol was monitored by TLC and the reaction was quenched with 30 mL water. The product was extracted with 30 mL EtOAc. The organic phases were then combined and dried with Na_2_SO_4_. After filtration, the solvent was evaporated under vaccum and purified by flash chromatography.

**General synthetic procedure B:** The dimethylamine derivative was dissolved in CH_2_Cl_2_ (2 mL) and iodomethane was added. The mixture was stirred for 2 hours at room temperature. All liquids were evaporated to obtain the product.

**General synthetic procedure C:** The acid chloride derivative, the bicyclic derivative and AlCl_3_ were dissolved in 5 mL of CH_2_Cl_2_ at room temperature and stirred for 2 hours. The reaction was quenched with 20 mL of iced water and the product was extracted with 20 mL of EtOAc, washed with brine and dried with Na_2_SO_4_. After filtration, the solvent was evaporated and the product was purified by flash chromatography.

**General synthetic procedure D:** The methoxy derivative and AlCl_3_ were dissolved in 5 mL of toluene. The mixture was heated at 120 °C with stirring for 1 hour. The solution was then cooled to room temperature and quenched with 50 mL of iced water. The product was extracted with 50 mL of EtOAc, washed with brine and dried with Na_2_SO_4_. After filtration, the solvent was evaporated and the crude product was purified by flash chromatography.

**(2-Butylbenzofuran-3-yl)(4-(2-(dimethylamino)ethoxy)-3,5-diiodophenyl)methanone**

Compound **WAG-003-1** was prepared according to general procedure A using (2-butylbenzofuran-3-yl)(4-hydroxy-3,5-diiodophenyl)methanone (100 mg; 0.183 mmol), 2-chloro-N,N-dimethylethanamine hydrochloride (144 mg; 1 mmol) and potassium carbonate (173 mg; 1.25 mmol). Purification of the crude product by flash chromatography (0 to 10% of MeOH in CH_2_Cl_2_) gave a yellow oil (60 mg; 53%).

**^1^H NMR (400 MHz, CDCl_3_)** *δ*: 8.21 (2H; s; H11,H12), 7.49 (1H; m; H3), 7.42 (1H; m; H6), 7.28 (2H; m; H5,H4), 4.17 (2H; m; H17), 2.93 (2H; m; H16), 2.83 (2H; m; H20), 2.42 (6H; s; H18,H19), 1.76 (2H; m; H21), 1.35 (2H; m; H22), 0.91 (3H; t; *J* = 7.0 Hz; H23).

**^13^C NMR (100 MHz, CDCl_3_)** *δ*: 187.9 (C9), 166.3 (C1), 161.6 (C15), 153.8 (C2), 140.8 (C11,C12), 138.4 (C10), 126.5 (C4), 124.8 (C5), 124.0 (C7), 121.2 (C6), 116.0 (C8), 111.2 (C3), 91.0 (C13,C14), 71.1 (C16), 58.9 (C17), 46.1 (C18,C19), 30.2 (C20), 28.3 (C21), 22.7 (C22), 13.9 (C23).

**HRMS-ESI (m/z)**: exact mass calculated for C_23_H_26_O_3_NI_2_ [M+H]^+^: 617.99966, found: 619.99875.

**IR (Diamant ATR, cm^-1^):** 3339, 2956, 2928, 1647, 1475, 1201, 748.


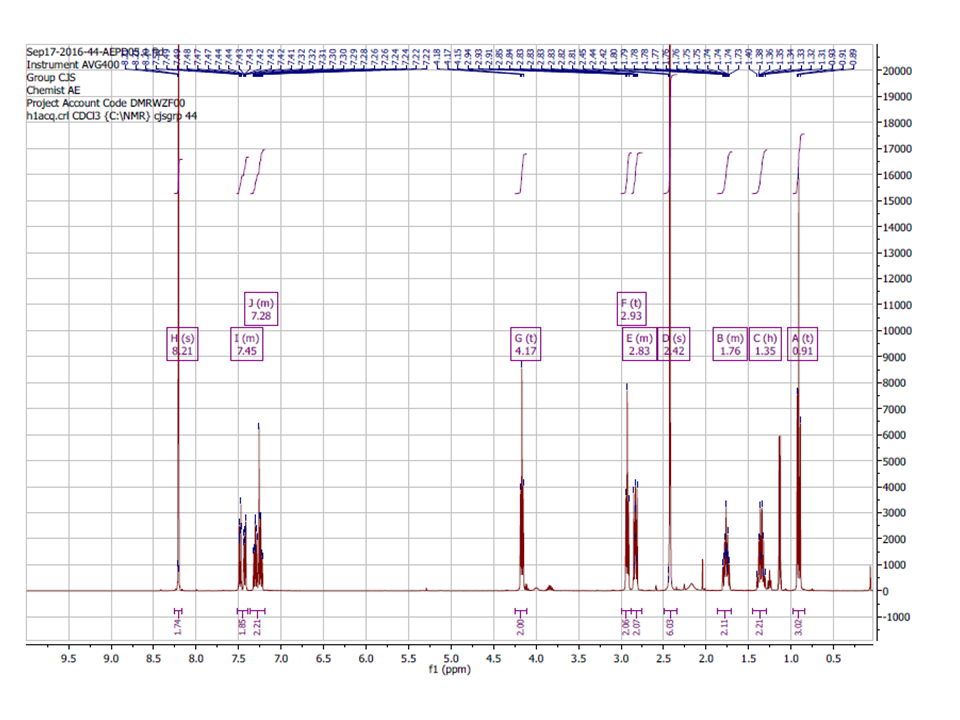


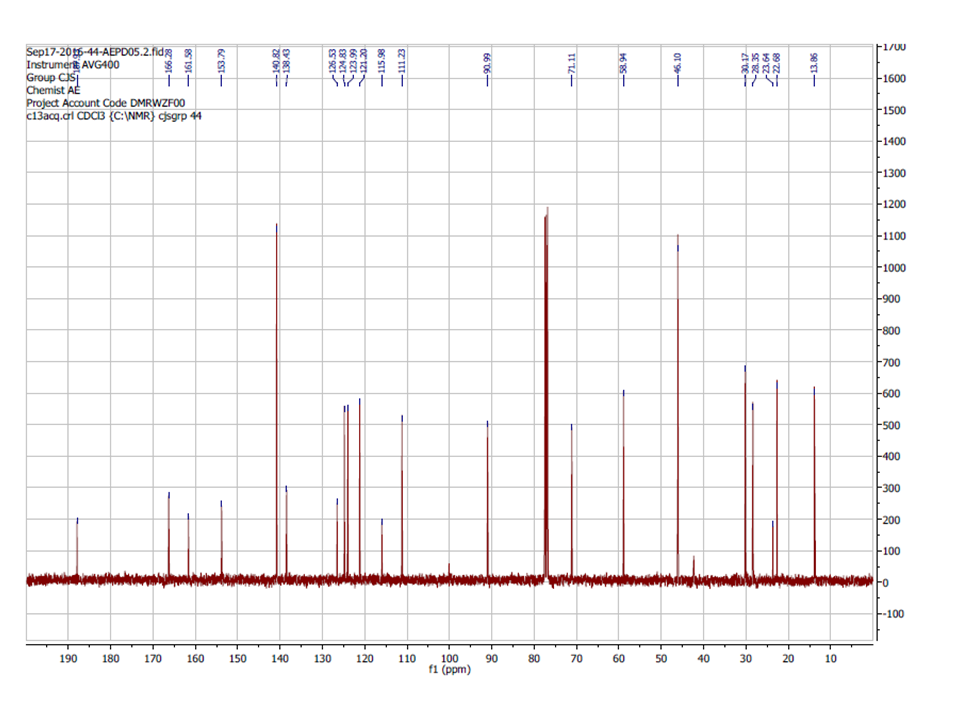


**2-(4-(2-Butylbenzofuran-3-carbonyl)-2,6-diiodophenoxy)-N,N,N-trimethylethanaminium iodide**

Compound **WAG-003** was prepared according to general procedure B using compound **1** (100 mg; 0.162 mmol) and iodomethane (70 µL; 0.485 mmol) to give a white powder (65 mg; 54%).

**^1^H NMR (400 MHz, DMSO)** *δ*: 8.21 (2H; s; H11,H12), 7.67 (1H; m; H3), 7.47 (1H; m; H6), 7.37 (1H; m; H4), 7.30 (1H; m; H5), 4.40 (2H; m; H17), 4.00 (2H; m; H16), 3.34 (9H; s; H18,H19,H20), 2.74 (2H; m; H21), 1.70 (2H; m; H22), 1.27 (2H; m; H23), 0.85 (3H; t; J = 7.5 Hz; H24).

**^13^C NMR (100 MHz, DMSO)** *δ*: 187.9 (C9), 166.2 (C1), 160.4 (C15), 153.6 (C2), 140.3 (C11,C12), 139.3 (C10), 126.6 (C4), 125.4 (C5), 124.5 (C7), 121.3 (C6), 116.1 (C8), 111.7 (C3), 92.9 (C13,C14), 67.2 (C16), 65.4 (C17), 53.9 (C18,C19,C20), 29.9 (C21), 28.1 (C22), 22.5 (C23), 13.9 (C24).

**HRMS-ESI (m/z)**: exact mass calculated for C_24_H_28_I_2_NO_3_ [M]^+^: 632.01531, found: 632.01440.

**IR (Diamant ATR, cm^-1^):** 3042, 2926, 2871, 1649, 1153, 950, 741.

**Mp :** 207 °C.


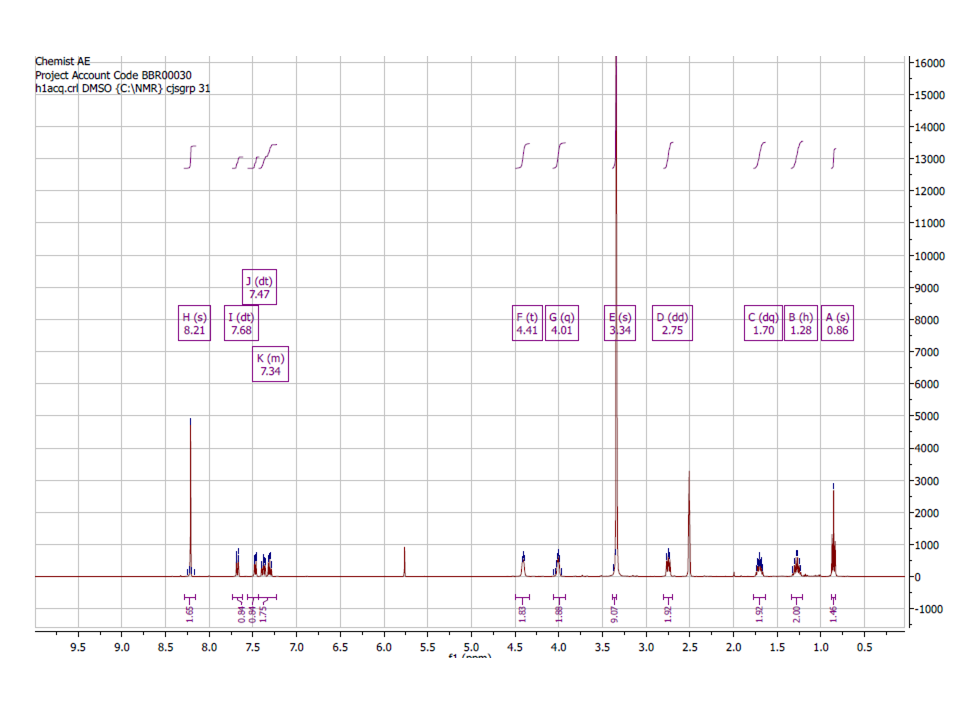


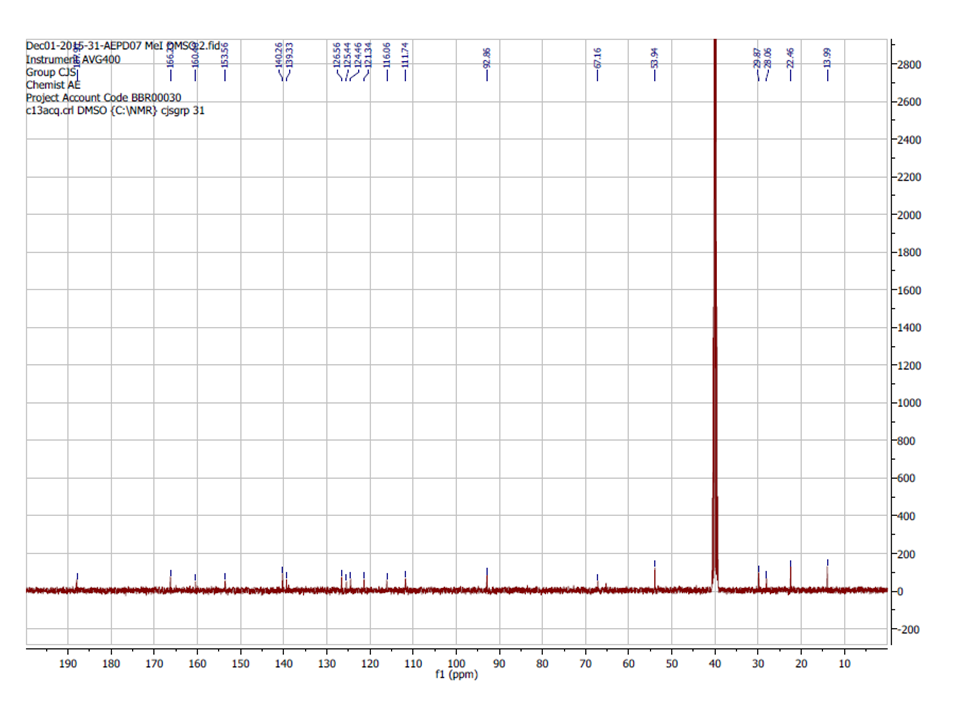


**(2-Butylbenzofuran-3-yl)(4-(3-(dimethylamino)propoxy)-3,5-diiodophenyl)methanone**

Compound **WAG-005-1** was prepared according to general procedure A using (2-butylbenzofuran-3-yl)(4-hydroxy-3,5-diiodophenyl)methanone (100mg; 0.183 mmol), 3-chloro-*N*,*N*-dimethylpropan-1-amine hydrochloride (158 mg; 1 mmol) and potassium carbonate (173 mg; 1.25 mmol). Purification of the crude product by flash chromatography (0 to 10% of MeOH in CH_2_Cl_2_) gave a yellow oil (110 mg; 95%).

**^1^H NMR (400 MHz, CDCl_3_)** *δ*: 8.20 (2H; s; H11,H12), 7.48 (1H; m; H3), 7.41 (1H; m; H6), 7.27 (2H; m; H4,H5), 4.11 (2H; m; H17), 2.84 (2H; m; H16), 2.62 (2H; m; H21), 2.32 (6H; s; H19,H20), 2.16 (2H; m; H17), 1.77 (2H; m; H22), 1.35 (2H; m; H23), 0.91 (3H; t; *J* = 7.0 Hz; H24).

**^13^C NMR (100 MHz, CDCl_3_)** *δ*: 187.9 (C9), 166.3 (C1), 161.4 (C15), 153.8 (C2), 140.8 (C11,C12), 138.4 (C10), 126.5 (C4), 124.8 (C5), 123.9 (C7), 121.2 (C6), 115.9 (C8), 111.2 (C3), 91.0 (C13,C14), 72.2 (C16), 56.4 (C18), 45.6 (C19,C20), 30.2 (C21), 28.3 (C17), 28.2 (C22), 22.6 (C23), 13.9 (C24).

**HRMS-ESI (m/z)**: exact mass calculated for C_24_H_28_O_3_NI_2_ [M+H]^+^: 632.01531, found: 632.01465.

**IR (Diamant ATR, cm^-1^):** 2960, 1641, 1280, 926, 752.


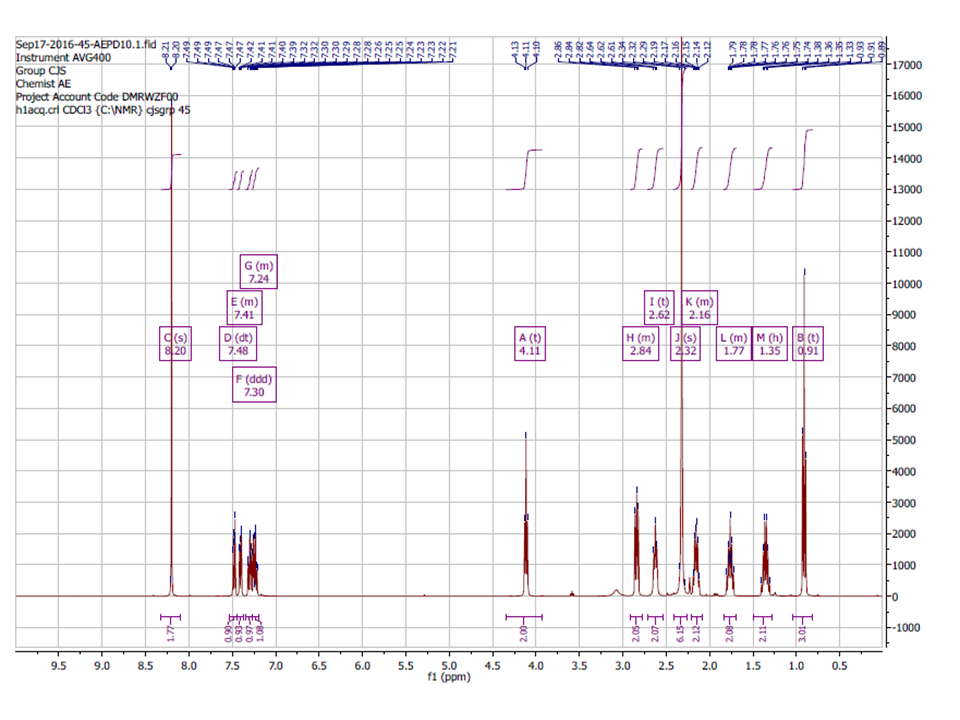


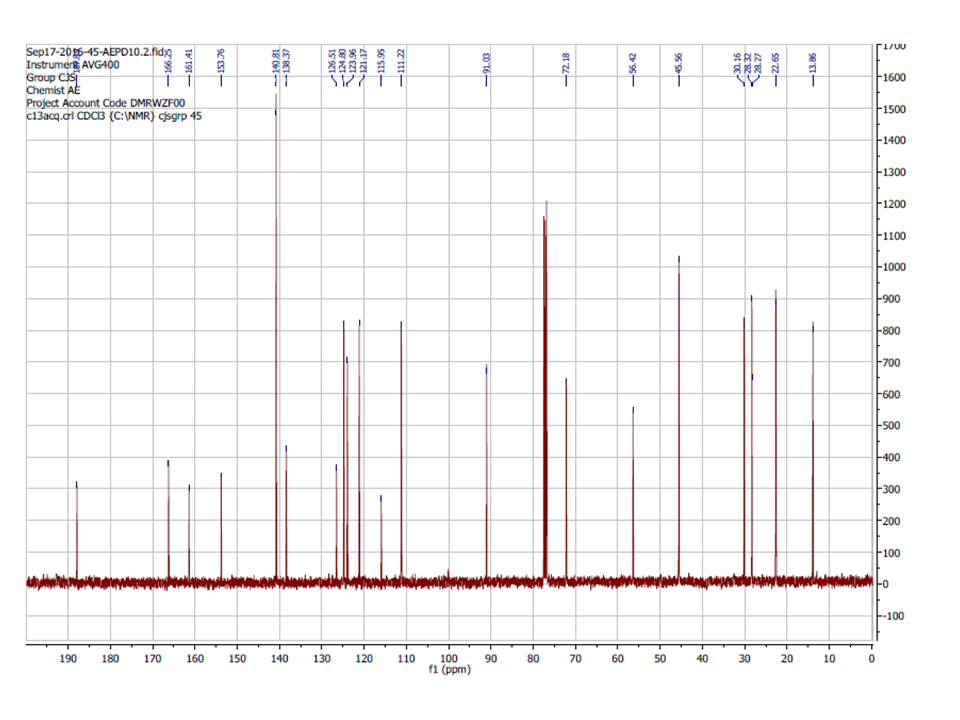


**3-(4-(2-Butylbenzofuran-3-carbonyl)-2,6-diiodophenoxy)-N,N,N-trimethylpropan-1-aminium iodide**

Compound **WAG-005** was prepared according to general procedure B using compound **3** (110 mg; 0.18 mmol) and iodomethane (80 µL; 0.54 mmol) to give a white powder (98 mg; 87%).

**^1^H NMR (400 MHz, DMSO)** *δ*: 8.18 (2H; s; H11,H12), 7.66 (1H; m; H3), 7.50 (1H; m; H6), 7.38 (1H; m; H5), 7.31 (1H; m; H4), 4.09 (2H; m; H18), 3.70 (2H; m; H16), 3.15 (9H; s; H19,H20,H21), 2.71 (2H; m; H22), 2.34 (2H; m; H17), 1.70 (2H; m; H23), 1.27 (2H; m; H24), 0.84 (3H; t; J = 7.5 Hz; H25).

**^13^C NMR (100 MHz, DMSO)** *δ*: 187.5 (C9), 165.6 (C1), 160.3 (C15), 153.1 (C2), 139.8 (C11,C12), 138.6 (C10), 126.1 (C4), 125.0 (C5), 124.0 (C7), 120.9 (C6), 115.6 (C8), 111.8 (C3), 92.3 (C13,C14), 70.0 (C16), 63.2 (C18), 52.3 (C19,C20,C21), 29.4 (C22), 27.6 (C17), 23.7 (C23), 22.0 (C24), 13.5 (C25).

**HRMS-ESI (m/z)**: exact mass calculated for C_25_H_30_I_2_NO_3_ [M]^+^: 646.03096, found: 646.03008.

**IR (Diamant ATR, cm^-1^):** 3007, 2944, 2860, 1647, 1243, 746.

**Mp :** 211 °C.


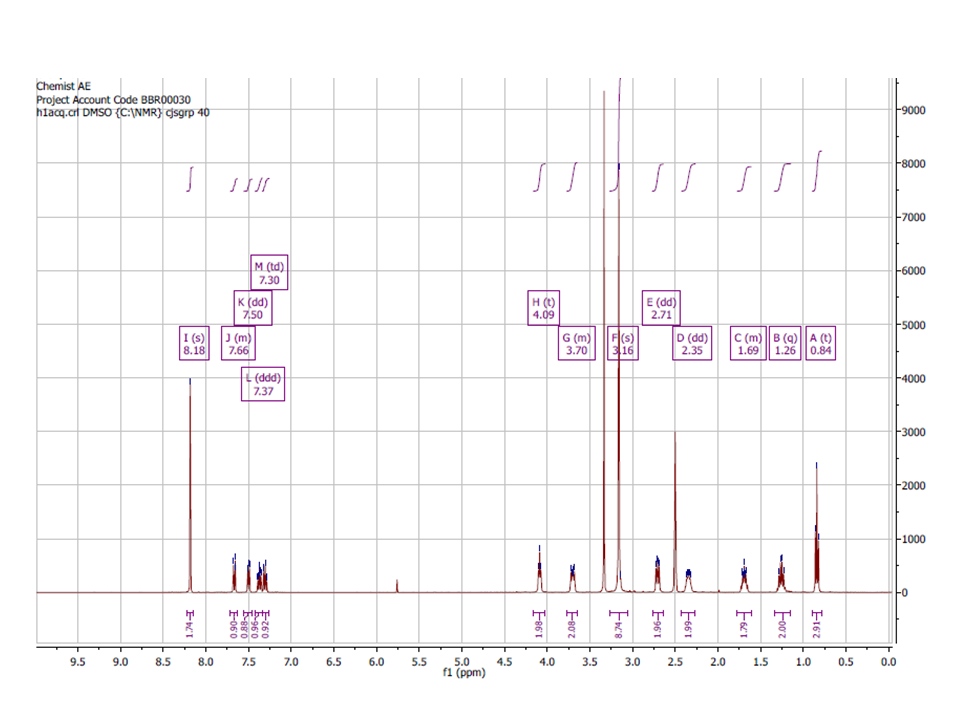


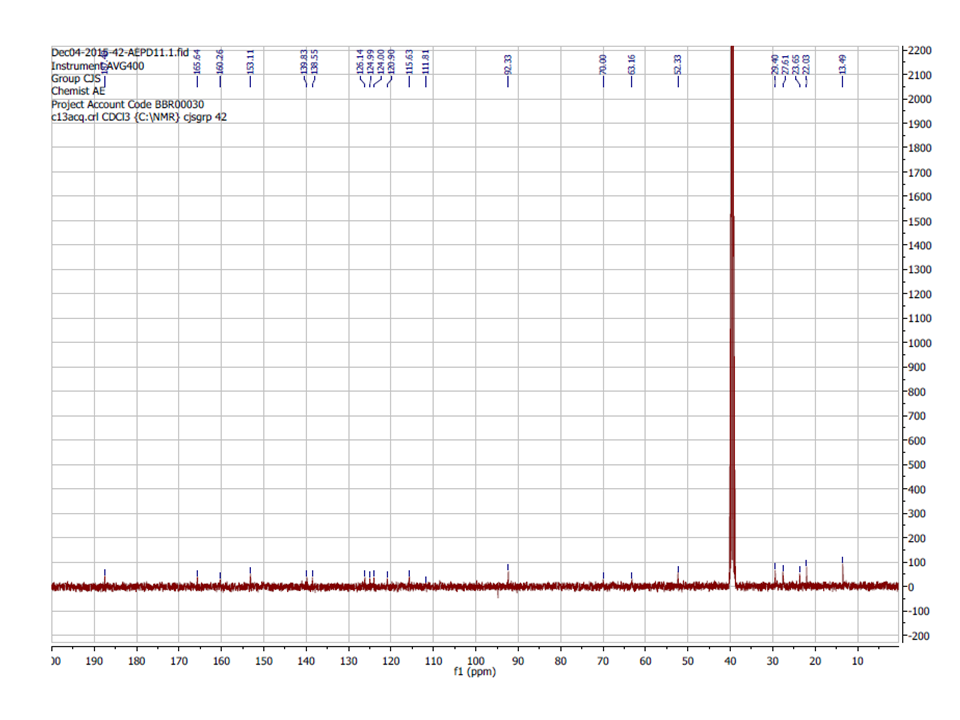


**(2-Butylbenzofuran-3-yl)(4-(3-(dimethylamino)propoxy)phenyl)methanone**

Compound **1a** was prepared according to general procedure A using (2-butylbenzofuran-3-yl)(4-hydroxyphenyl)methanone (200 mg; 0.68 mmol), 3-chloro-*N*,*N*-dimethylpropan-1-amine hydrochloride (591 mg; 3.74 mmol) and potassium carbonate (638 mg; 4.6 mmol). Purification of the crude product by flash chromatography (0 to 10% of MeOH in CH_2_Cl_2_) gave a colourless oil (205 mg; 80%).

**^1^H NMR (400 MHz, CDCl_3_)** *δ*: 7.82 (2H; d; *J* = 9.0 Hz; H11,H12), 7.46 (1H; m; H6), 7.35 (1H; m; H3), 7.25 (1H; m; H4), 7.17 (1H; m; H5), 6.94 (2H; d; *J* = 9.0 Hz; H13,H14), 4.10 (2H; t; *J* = 6.5 Hz; H16), 2.91 (2H; m; H18), 2.46 (2H; m; H21), 2.25 (6H; s; H19,H20), 1.99 (2H; m; H17), 1.74 (2H; m; H22), 1.35 (2H; m; H23), 0.88 (3H; t; *J* = 7.5 Hz; H24).

**^13^C NMR (100 MHz, CDCl_3_)** *δ*: 190.6 (C9), 164.7 (C1), 163.0 (C15), 153.7 (C2), 131.8 (C10,C11,C12), 127.3 (C4), 124.2 (C5), 123.4 (C7), 121.3 (C6), 116.9 (C8), 114.2 (C13,C14), 111.0 (C3), 66.5 (C16), 56.3 (C18), 45.6 (C19,C20), 30.3 (C21), 27.9 (C17), 27.5 (C22), 22.5 (C23), 13.8 (C24).

**HRMS-ESI (m/z)**: exact mass calculated for C_24_H_30_NO_3_ [M+H]^+^: 380.22202, found:380.22161.

**IR (Diamant ATR, cm^-1^):** 2956, 2873, 2764, 1572, 1242, 951, 748.


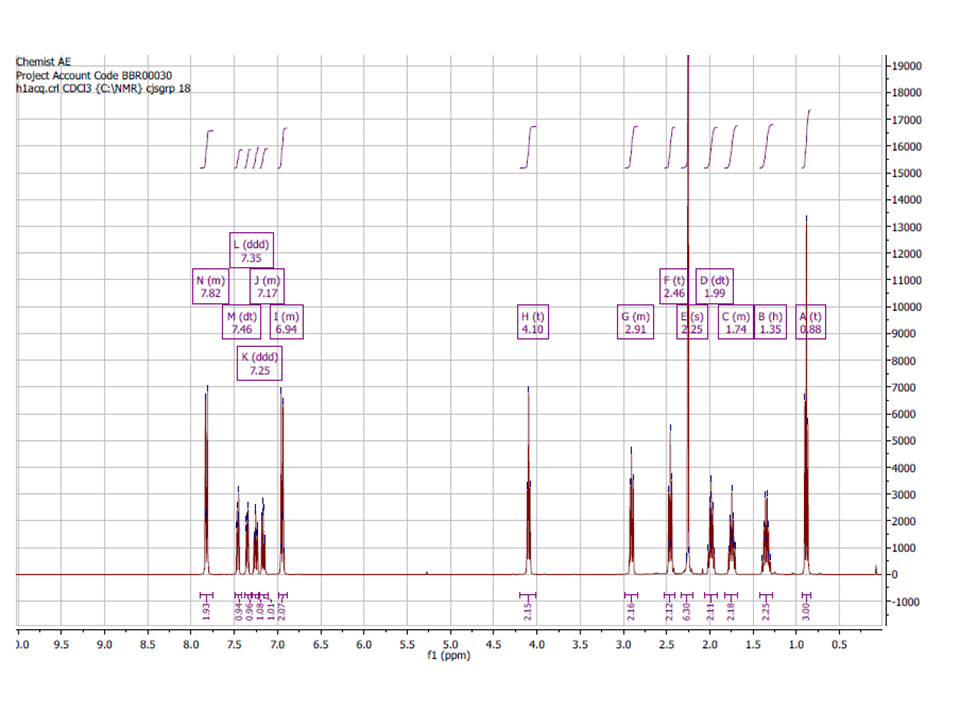


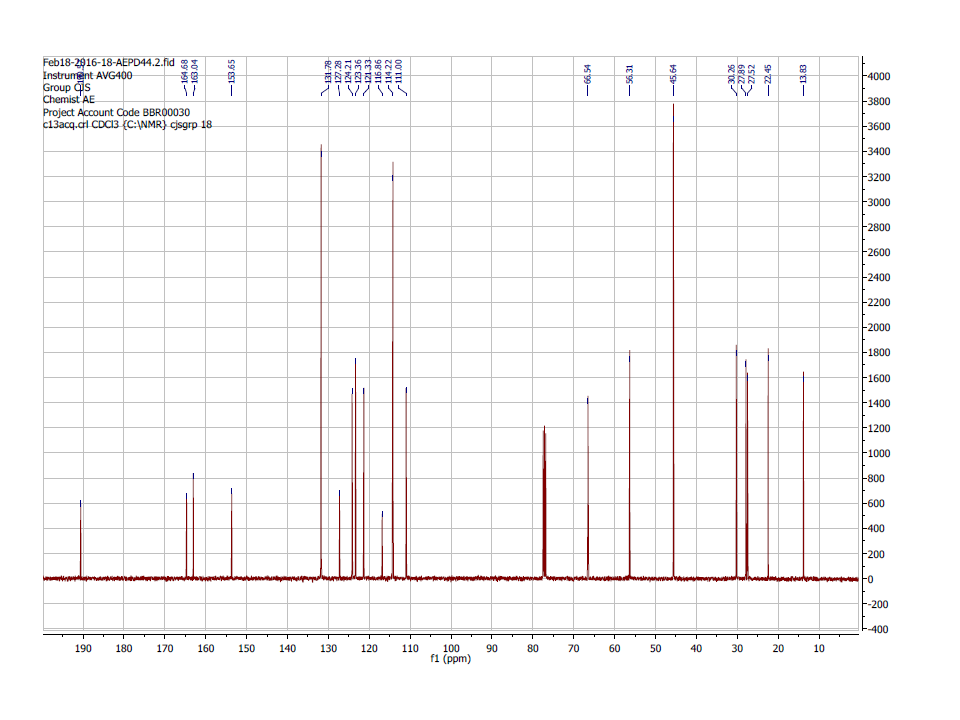


**3-(4-(2-Butylbenzofuran-3-carbonyl)phenoxy)-N,N,N-trimethylpropan-1-aminium iodide**

Compound **2a** was prepared according to general procedure B using compound **1a** (200 mg; 0.53 mmol) and iodomethane (0.1 mL; 1.6 mmol) to give a white powder (100 mg; 37 %).

**^1^H NMR (400 MHz, CDCl_3_)** *δ*: 7.76 (2H; m; H11,H12), 7.43 (1H; m; H6), 7.23 (1H; m; H3), 7.18 (1H; m; H4), 7.14 (1H; m; H5), 6.95 (2H; m; H13,H14), 4.20 (2H; m; H18), 3.91 (2H; m; H16), 3.48 (9H; s; H19,H20,H21), 2.86 (2H; m; H22), 2.36 (2H; m; H17), 1.70 (2H; m; H23), 1.31 (2H; m; H24), 0.85 (3H; t; J = 7.5 Hz; H25).

**^13^C NMR (100 MHz, CDCl_3_)** *δ*: 190.6 (C9), 165.0 (C1), 161.8 (C15), 153.6 (C2), 132.6 (C10), 131.7 (C11,C12), 127.1 (C4), 124.3 (C5), 123.5 (C7), 121.2 (C6), 116.6 (C8), 114.4 (C13,C14), 111.1 (C3), 64.6 (C16), 64.3 (C18), 54.1 (C19,C20,C21), 30.2 (C22), 27.9 (C23), 23.7 (C17), 22.4 (C24), 13.8 (C25).

**HRMS-ESI (m/z)**: exact mass calculated for C_25_H_32_O_3_N [M]^+^: 394.23767, found: 394.23721.

**IR (Diamant ATR, cm^-1^):** 2960, 2926, 1472, 1102, 905, 777.

**Mp :** 120 °C.


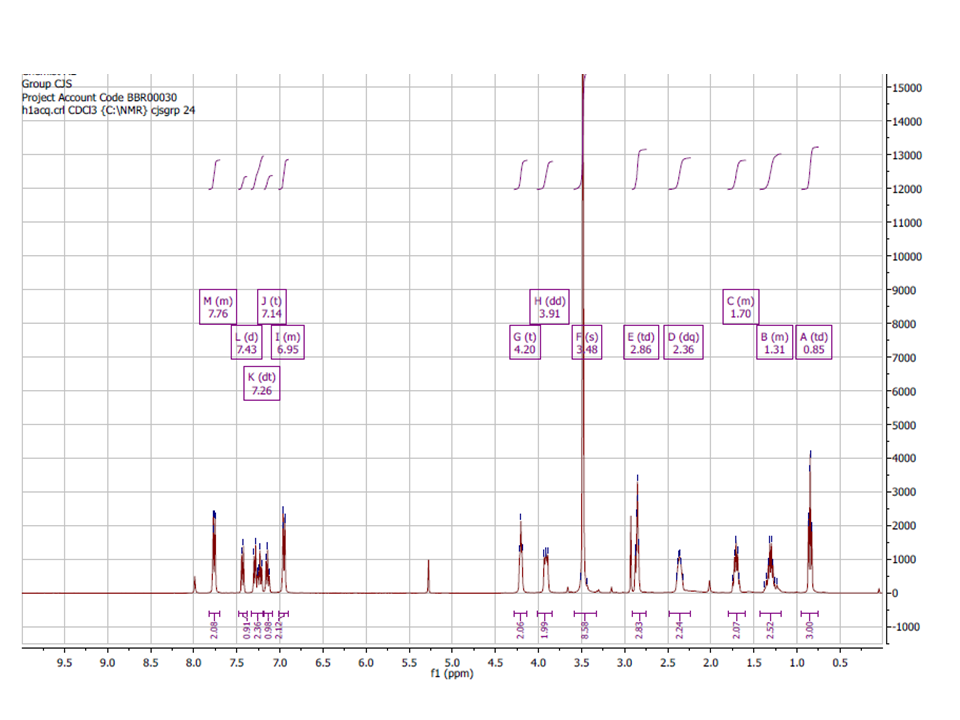


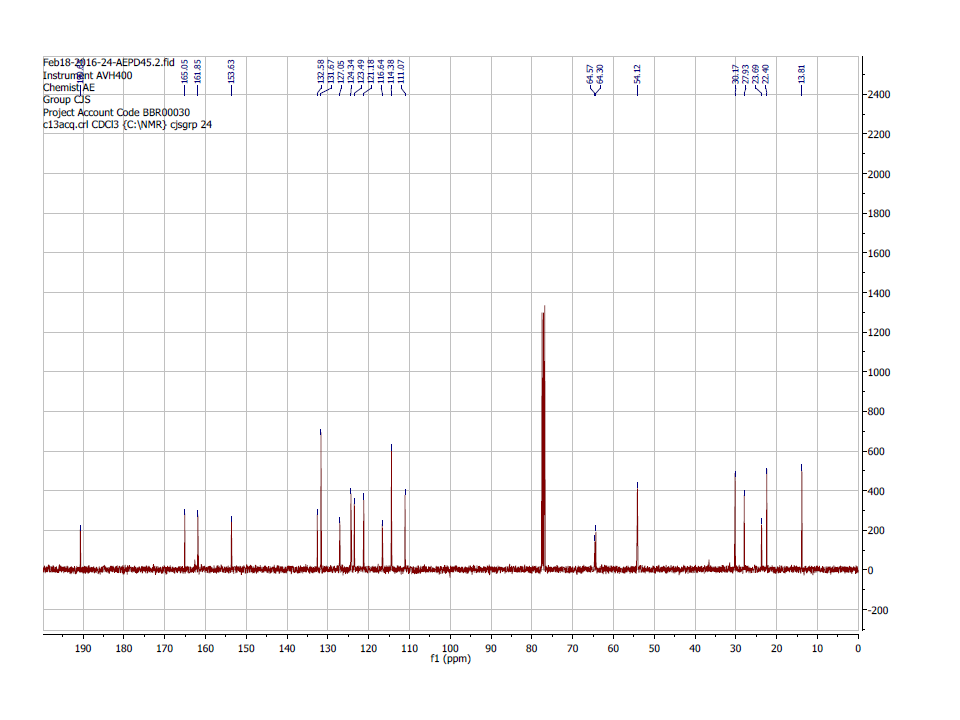


**(4-Methoxyphenyl)(2-methylbenzo[b]thiophen-3-yl)methanone**

Compound **5c** was prepared according to general procedure C using 2-methylbenzothiophene (300 mg; 2 mmol), 4-methoxybenzoyl chloride (0.27 mL; 2 mmol) and aluminium trichloride (524 mg; 4 mmol). Purification of the crude product by flash chromatography (0 to 10% of EtOAc in cyclohexane) gave a yellow oil (100 mg; 18%).

**^1^H NMR (400 MHz, CDCl_3_)** *δ*: 7.82 (2H; d; *J* = 8.0 Hz; H11,H12), 7.76 (1H; m; H6), 7.49 (1H; m; H3), 7.26 (2H; m; H4,H5), 6.92 (2H; d; *J* = 8.0 Hz; H13,H14), 3.86 (3H; s; H16), 2.49 (3H; s; H17).

**^13^C NMR (100 MHz, CDCl_3_)** *δ*: 192.3 (C9), 164.0 (C15), 144.3 (C1), 139.3 (C2), 138.2 (C8), 132.8 (C10), 132.3 (C11,C12), 131.4 (C4), 124.7 (C5), 124.3 (C6), 123.3 (C3), 121.9 (C7), 114.0 (C13,C14), 55.6 (C16), 15.7 (C17).

**HRMS-ESI (m/z)**: exact mass calculated for C_17_H_15_O_2_S [M+H]^+^: 283.07873, found: 283.07874.

**IR (Diamant ATR, cm^-1^):** 2951, 2821, 1565, 1176, 1135, 752.


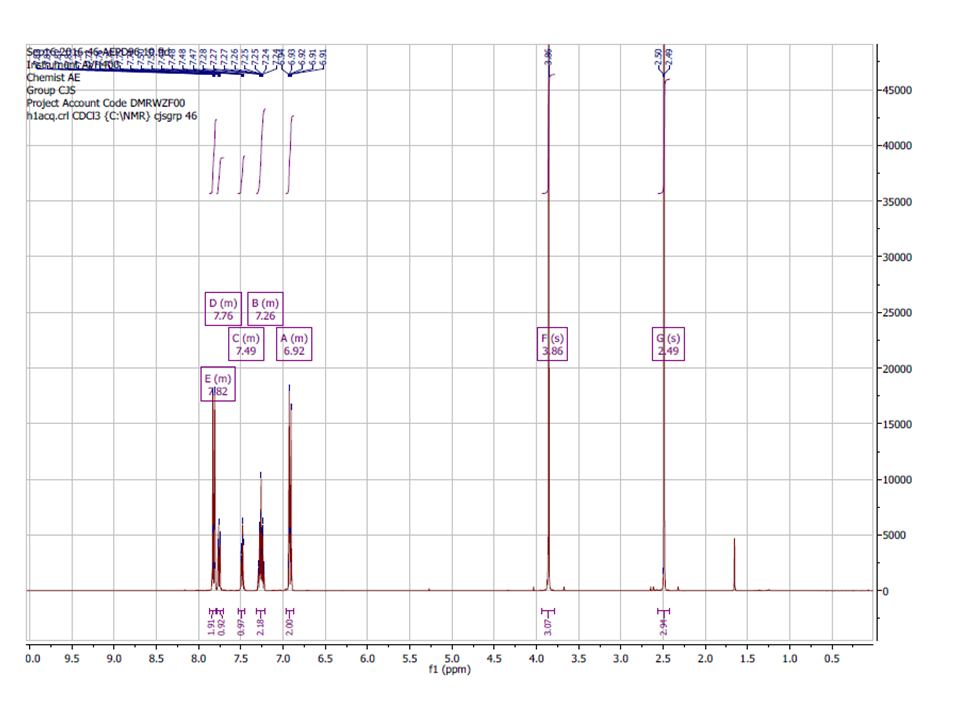


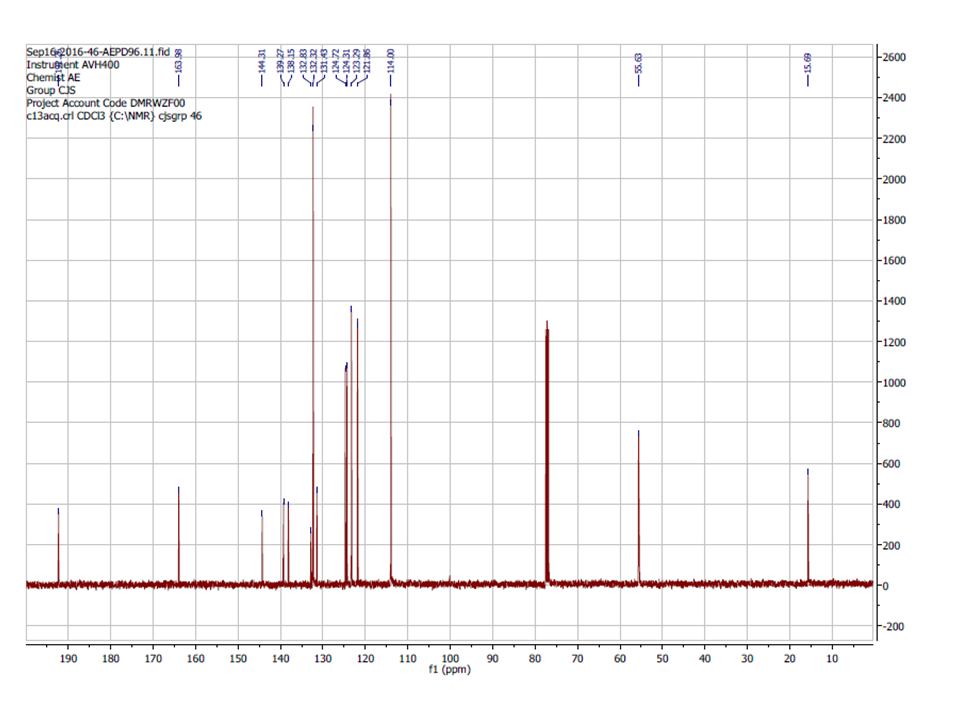


**(4-Hydroxyphenyl)(2-methylbenzo[b]thiophen-3-yl)methanone**

Compound **6c** was prepared according to general procedure D using compound **5c** (300 mg; 1.1 mmol) and aluminium trichloride (293 mg; 2.2 mmol). Purification of the crude product by flash chromatography (0 to 20% of EtOAc in cyclohexane) gave a yellow oil (132 mg; 24%).

**^1^H NMR (400 MHz, CDCl_3_)** *δ*: 7.72 (2H; d; *J* = 8.0 Hz; H11, H12), 7.44 (1H; m; H6), 7.23 (3H; m; H3, H4, H5), 6.83 (2H; d; *J* = 8.0 Hz; H13,H14), 2.45 (3H; s; H16).

**^13^C NMR (100 MHz, CDCl_3_)** *δ*: 193.3 (C9), 161.5 (C15), 144.9 (C1), 139.2 (C2), 138.1 (C8), 132.9 (C11,C12), 132.6 (C10), 130.9 (C4), 124.8 (C5), 124.4 (C6), 123.2 (C3), 121.9 (C7), 115.8 (C13,C14), 15.7 (C16).

**HRMS-ESI (m/z)**: exact mass calculated for C_16_H_13_O_2_S [M+H]^+^: 269.06308, found: 269.06301.

**IR (Diamant ATR, cm^-1^):** 2952, 2832, 1566, 1178, 1124, 756.


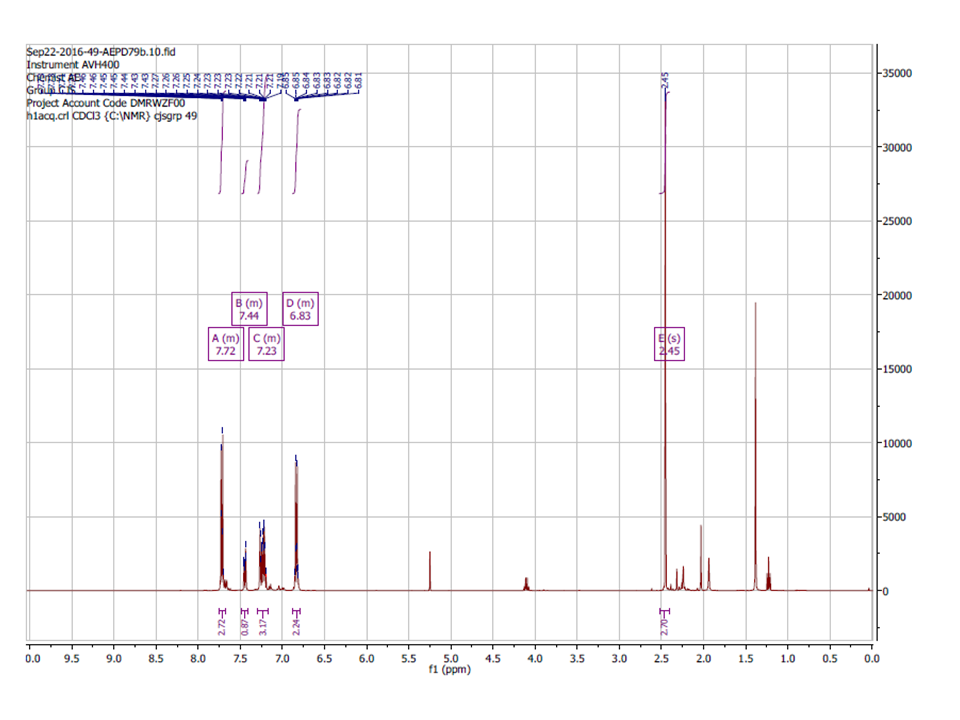


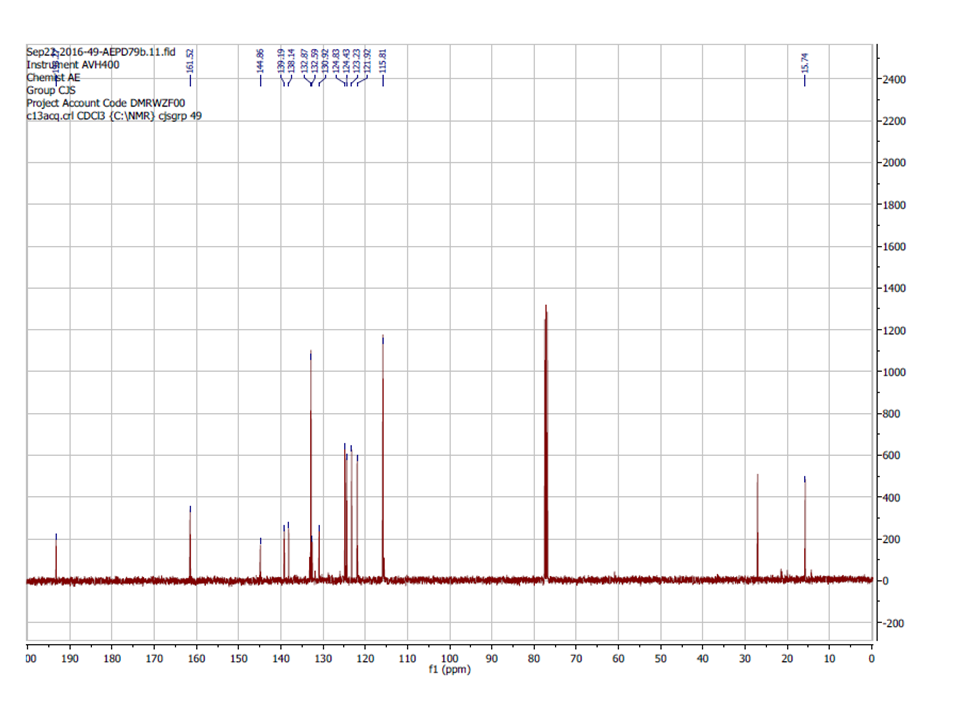


**(4-(3-(Dimethylamino)propoxy)phenyl)(2-methylbenzo[b]thiophen-3-yl)methanone**

Compound **1c** was prepared according to general procedure A using compound **6c** (50 mg; 0.19 mmol), 3-chloro-*N*,*N*-dimethylpropan-1-amine hydrochloride (165 mg; 1.045 mmol) and potassium carbonate (178 mg; 1.29 mmol). Purification of the crude product by flash chromatography (0 to 10% of MeOH in CH_2_Cl_2_) gave a yellow oil (50 mg; 66%).

**^1^H NMR (400 MHz, CDCl_3_)** *δ*: 7.73 (2H; d; *J* = 8.0 Hz; H11,H12), 7.71 (1H; m; H6), 7.42 (1H; m; H3), 7.20 (2H; m; H4,H5), 6.85 (2H; d; *J* = 8.0 Hz; H13,H14), 4.02 (2H; m; H16), 2.42 (3H + 2H; m; H18,H21), 2.21 (6H; s; H19,H20), 1.93 (2H; m; H17).

**^13^C NMR (100 MHz, CDCl_3_)** *δ*: 192.3 (C9), 163.5 (C15), 144.3 (C1), 139.3 (C2), 138.1 (C8), 132.8 (C10), 132.3 (C11,C12), 131.3 (C4), 124.7 (C5), 124.3 (C6), 123.3 (C3), 121.9 (C7), 114.5 (C13,C14), 66.5 (C16), 56.3 (C18), 45.5 (C19,C20), 27.4 (C17), 15.7 (C21).

**HRMS-ESI (m/z)**: exact mass calculated for C_21_H_24_NO_2_S [M+H]^+^: 354.15223, found: 354.15246.

**IR (Diamant ATR, cm^-1^):** 2943, 2816, 1571, 1176, 1164, 754.


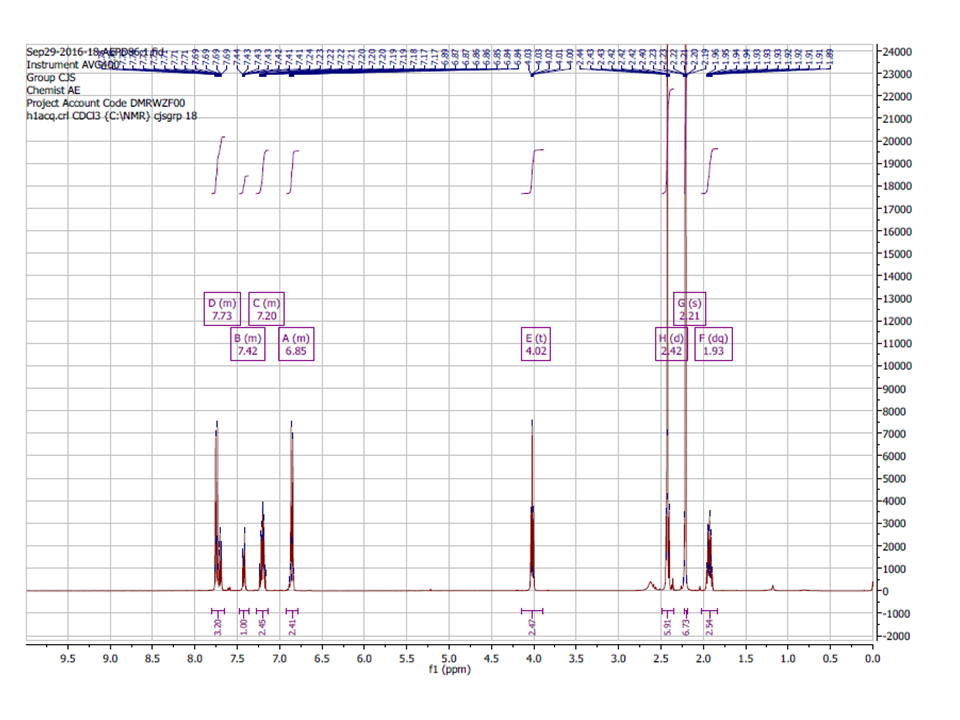


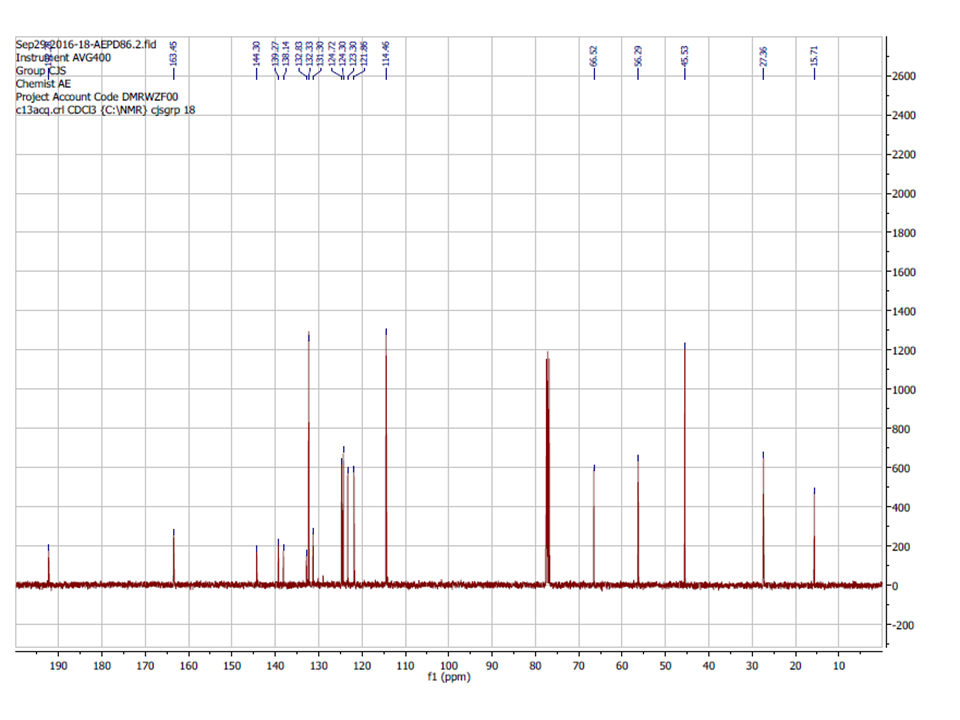


***N*,*N*,*N*-Trimethyl-3-(4-(2-methylbenzo[b]thiophene-3-carbonyl)phenoxy)propan-1-aminium iodide**

Compound **2c** was prepared according to general procedure B using compound **1c** (50 mg; 0.14 mmol) and iodomethane (0.1 mL; 0.42 mmol) to give a yellow powder (60 mg; 84%).

**^1^H NMR (400 MHz, DMSO)** *δ*: 7.99 (1H; m; H6), 7.76 (2H; m; H11,H12), 7.36 (3H; m; H3,H4,H5), 7.09 (2H; m; H13,H14), 4.17 (2H; m; H16), 3.50 (2H; m; H18), 3.11 (9H; s; H19,H20,H21), 2.45 (3H; s; H22), 2.22 (2H; m; H17).

**^13^C NMR (100 MHz, DMSO)** *δ*: 191.1 (C9), 162.5 (C15), 143.7 (C1), 138.5 (C2), 137.4 (C8), 132.1 (C10), 131.8 (C11,C12), 130.8 (C4), 124.8 (C5), 124.5 (C6) 122.5 (C3), 122.3 (C7), 114.7 (C13,C14), 65.2 (C16), 62.8 (C18), 52.4 (C19,C20,C21), 22.5 (C17), 15.3 (C22).

**HRMS-ESI (m/z)**: exact mass calculated for C_22_H_26_O_2_NS [M]^+^: 368.16788, found: 368.16771.

**IR (Diamant ATR, cm^-1^):** 2998, 2958, 1481, 1086, 831.

**Mp :** 211 °C.


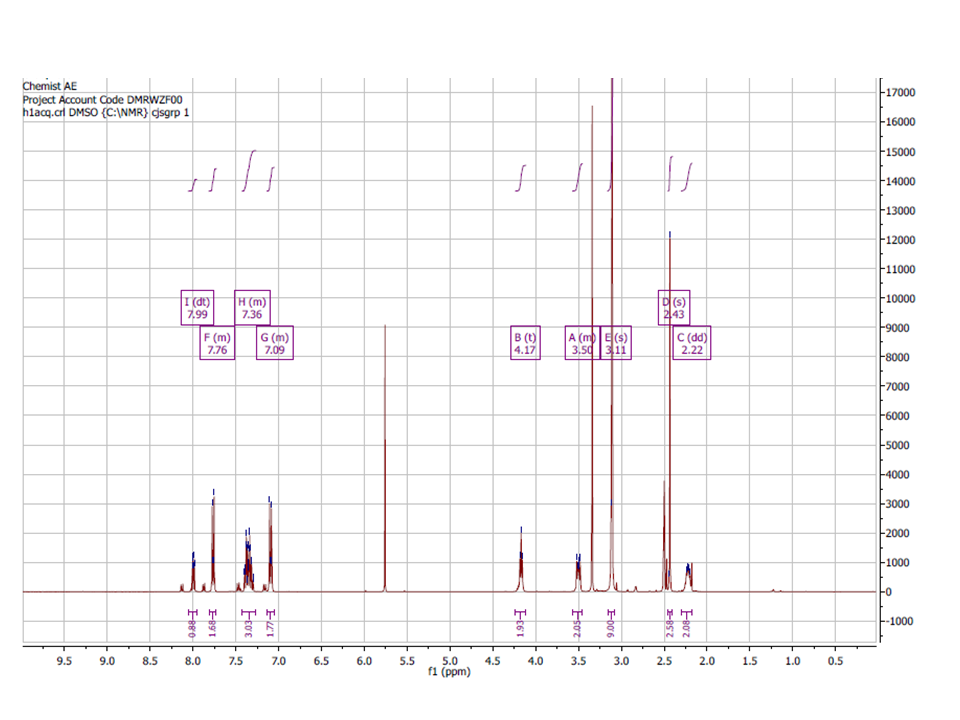


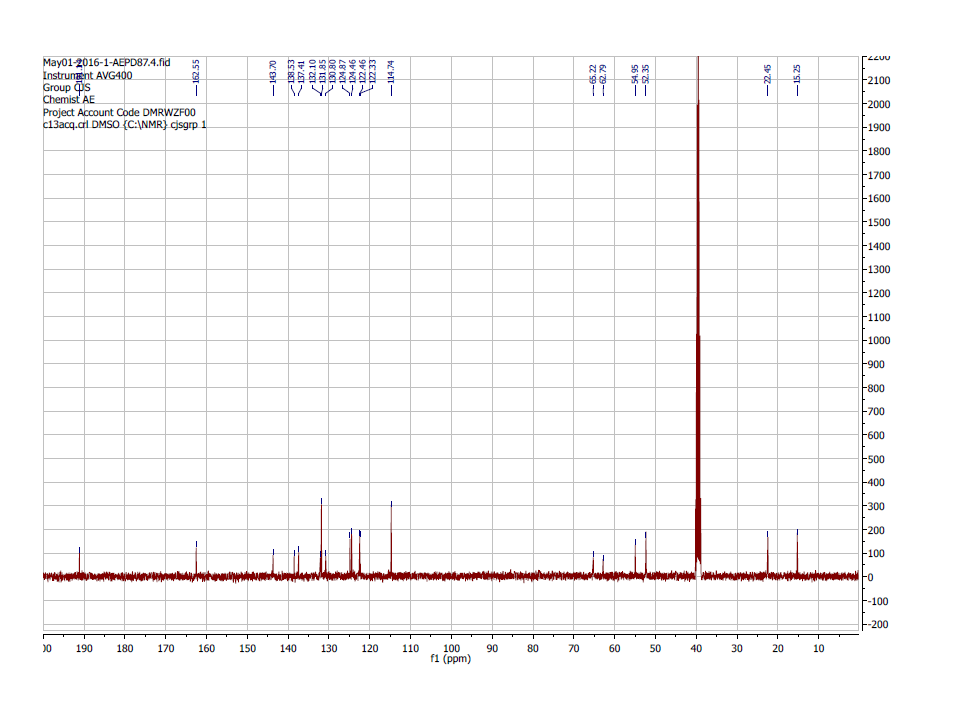


**(4-Methoxyphenyl)(2-methylbenzofuran-3-yl)methanone**

Compound **5b** was prepared according to general procedure C using 2-methylbenzofuran (0.37 mL; 2.94 mmol), 4-methoxybenzoyl chloride (0.4 mL; 2.94 mmol) and aluminium trichloride (770 mg; 5.88 mmol). Purification of the crude product by flash chromatography (0 to 10% of EtOAc in cyclohexane) gave a yellow oil (666 mg; 85%).

**^1^H NMR (400 MHz, CDCl_3_)** *δ*: 7.84 (2H; d; *J* = 8.0 Hz; H11,H12), 7.44 (2H; m; H3,H6), 7.27 (1H; m; H5), 7.19 (1H; m; H4), 6.96 (2H; d; *J* = 8.0 Hz; H13,H14), 3.88 (3H; s; H16), 2.56 (3H; s; H17).

**^13^C NMR (100 MHz, CDCl_3_)** *δ*: 190.4 (C9), 163.4 (C15), 160.8 (C2), 153.6 (C1), 131.8 (C7), 131.7 (C11,C12), 127.2 (C10), 124.2 (C4), 123.4 (C5), 121.2 (C6), 117.1 (C8), 113.7 (C13,C14), 110.8 (C3), 55.5 (C16), 14.5 (C17).

**HRMS-ESI (m/z)**: exact mass calculated for C_17_H_15_O_3_ [M+H]^+^: 267.10157, found: 267.10161.

**IR (Diamant ATR, cm^-1^):** 3008, 2936, 1629, 1256, 758.


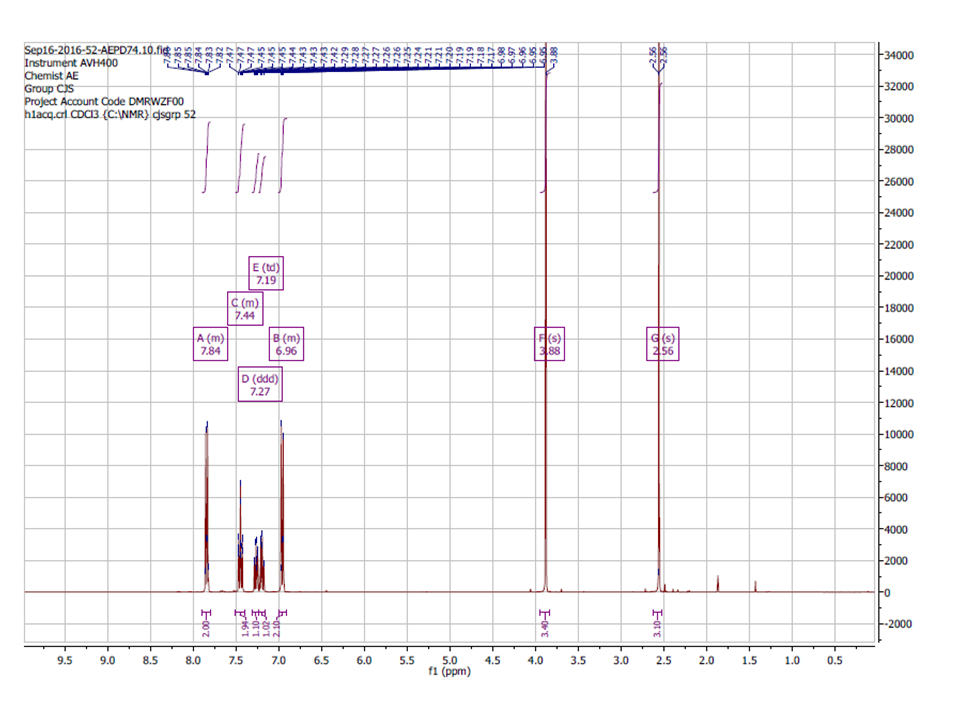


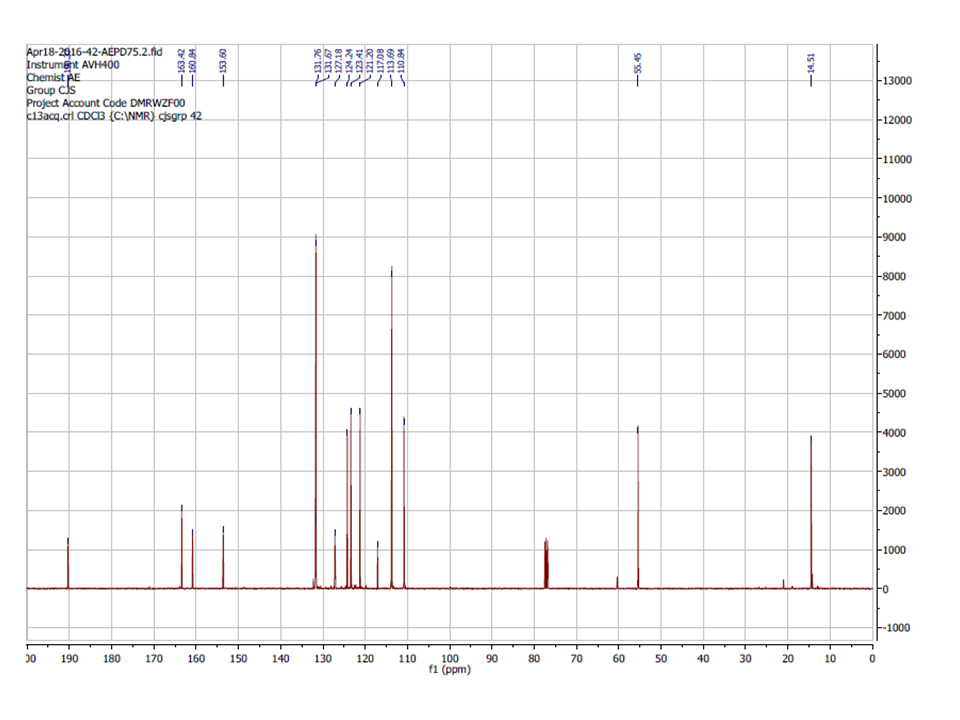


**(4-Hydroxyphenyl)(2-methylbenzofuran-3-yl)methanone**

Compound **6b** was prepared according to general procedure D using compound **5b** (400 mg; 1.5 mmol) and aluminium trichloride (400 mg; 3 mmol). Purification of the crude product by flash chromatography (0 to 20% of EtOAc in cyclohexane) gave a yellow oil (360 mg; 95%).

**^1^H NMR (400 MHz, CDCl_3_)** *δ*: 7.79 (2H; d; *J* = 8.0 Hz; H11,H12), 7.45 (2H; m; H3,H6), 7.28 (1H; m; H5), 7.21 (1H; m; H4), 6.97 (2H; d; *J* = 8.0 Hz; H13,H14), 2.56 (3H; s; H16).

**^13^C NMR (100 MHz, CDCl_3_)** *δ*: 192.1 (C9), 161.6 (C15), 161.4 (C2), 153.8 (C1), 132.4 (C11,C12), 131.1 (C7), 127.1 (C10), 124.6 (C4), 123.7 (C5), 121.3 (C6), 117.2 (C8), 115.7 (C13,C14), 111.0 (C3), 14.8 (C16).

**HRMS-ESI (m/z)**: exact mass calculated for C_16_H_13_N_3_ [M+H]^+^: 253.08592, found: 253.08597.

**IR (Diamant ATR, cm^-1^):** 3005, 2912, 1638, 1245, 748.


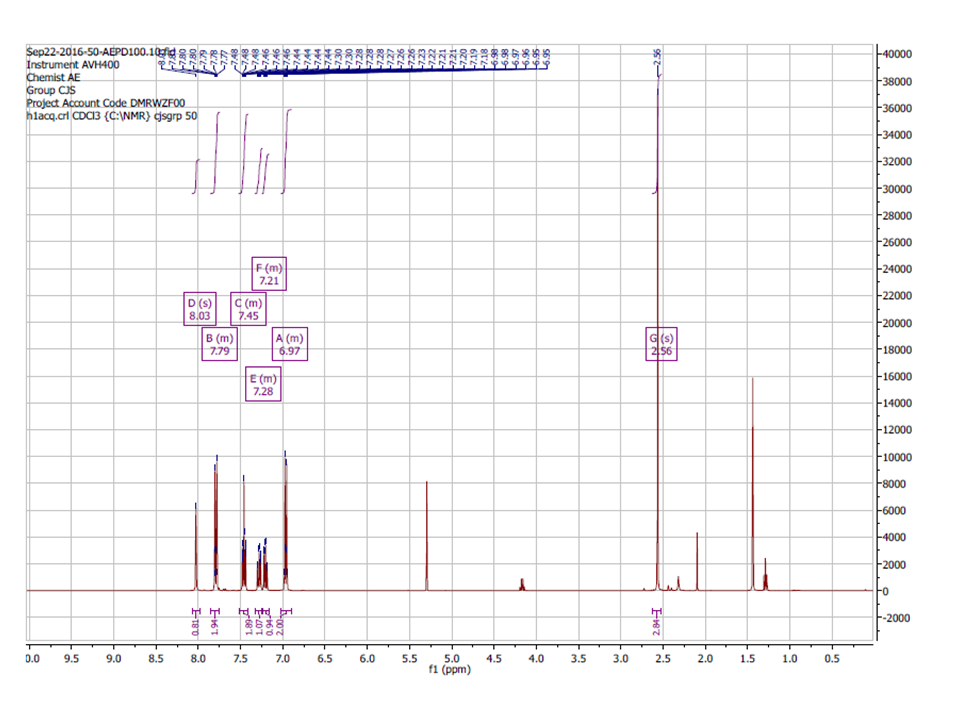


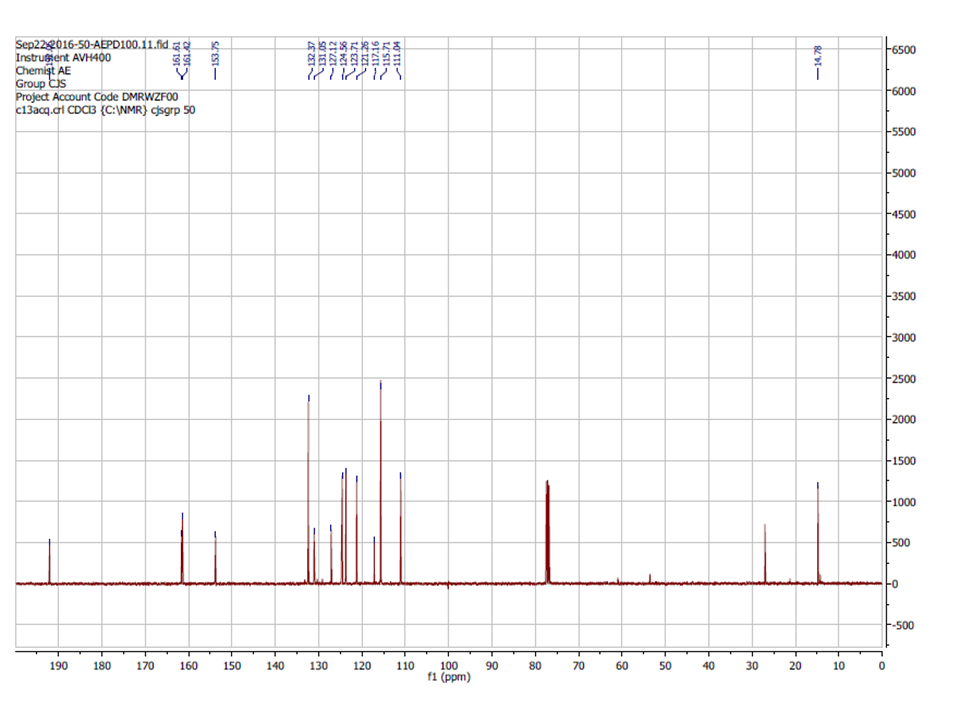


**(4-(3-(Dimethylamino)propoxy)phenyl)(2-methylbenzofuran-3-yl)methanone**

Compound **1b** was prepared according to general procedure A using compound **6b** (340 mg; 1.35 mmol), 3-chloro-*N*,*N*-dimethylpropan-1-amine hydrochloride (1.2 g; 7.43 mmol) and potassium carbonate (1.3 g; 9.18 mmol). Purification of the crude product by flash chromatography (0 to 5% of MeOH in CH_2_Cl_2_) gave a yellow oil (204 mg; 96%).

**^1^H NMR (400 MHz, CDCl_3_)** *δ*: 7.79 (2H; d; *J* = 8.0 Hz; H11,H12), 7.40 (2H; m; H3,H6), 7.23 (1H; m; H5), 7.16 (1H; m; H4), 6.93 (2H; d; *J* = 8.0 Hz; H13,H14), 4.07 (2H; m; H16), 2.52 (3H; s; H21), 2.45 (2H; m; H18), 2.24 (6H; s; H19,H20), 1.97 (2H; m; H17).

**^13^C NMR (100 MHz, CDCl_3_)** *δ*: 190.5 (C9), 162.9 (C15), 160.8 (C2), 153.6 (C1), 131.7 (C7), 131.6 (C11,C12), 127.2 (C10), 124.2 (C4), 123.4 (C5), 121.2 (C6), 117.1 (C8), 114.2 (C13,C14), 110.8 (C3), 66.4 (C16), 56.2 (C18), 45.5 (C19,C20), 27.4 (C17), 14.5 (C21).

**HRMS-ESI (m/z)**: exact mass calculated for C_21_H_24_O_3_N [M+H]^+^: 338.17507, found: 338.17596.

**IR (Diamant ATR, cm^-1^):** 3004, 2929, 1642, 1254, 751.


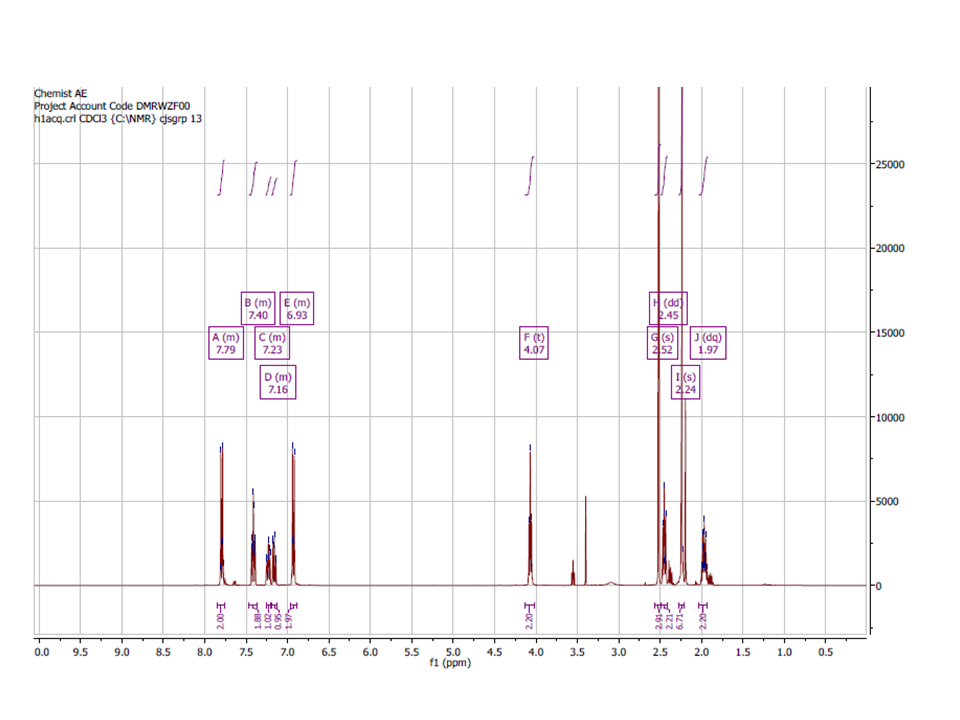


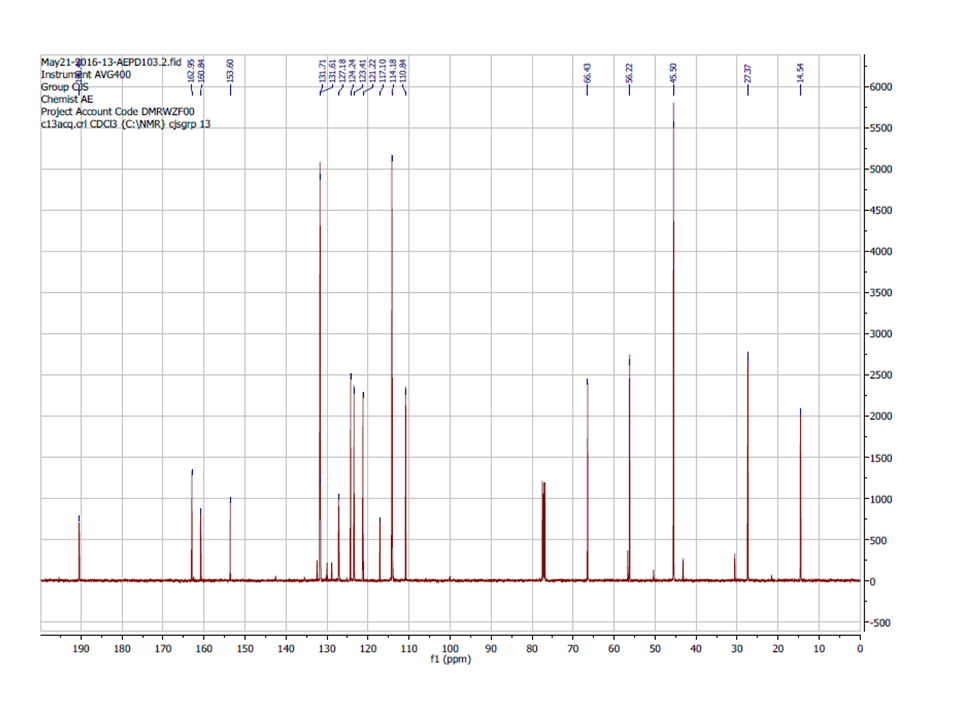


***N,N,N*-trimethyl-3-(4-(2-methylbenzofuran-3-carbonyl)phenoxy)propan-1-aminium iodide**

Compound **2b** was prepared according to general procedure B using compound **1b** (200 mg; 0.6 mmol) and iodomethane (110 µL; 1.8 mmol) to give a yellow powder (203 mg; 72%).

**^1^H NMR (400 MHz, DMSO)** *δ*: 7.79 (2H; m; H11,H12), 7.62 (1H; m; H6), 7.41 (1H; m; H3), 7.33 (1H; m; H5), 7.25 (1H; m; H4), 7.11 (2H; m; H13,H14), 4.18 (2H; m; H16), 3.55 (2H; m; H18), 3.15 (9H; s; H19,H20,H21), 2.46 (3H; s; H22), 2.24 (2H; m; H17).

**^13^C NMR (100 MHz, DMSO)** *δ*: 189.5 (C9), 162.0 (C15), 160.6 (C2), 152.9 (C1), 131.7 (C7), 131.4 (C11,C12), 126.7 (C10), 124.6 (C4), 123.7 (C5), 120.6 (C6), 116.3 (C8), 114.5 (C13,C14), 111.0 (C3), 65.1 (C16), 62.8 (C18), 52.3 (C19,C20,C21), 22.5 (C17), 14.5 (C22).

**HRMS-ESI (m/z)**: exact mass calculated for C_22_H_26_O_3_N [M]^+^: 352.19072, found: 352.19061.

**IR (Diamant ATR, cm^-1^):** 3009, 2923, 1630, 1242, 750.

**Mp :** 119 °C.


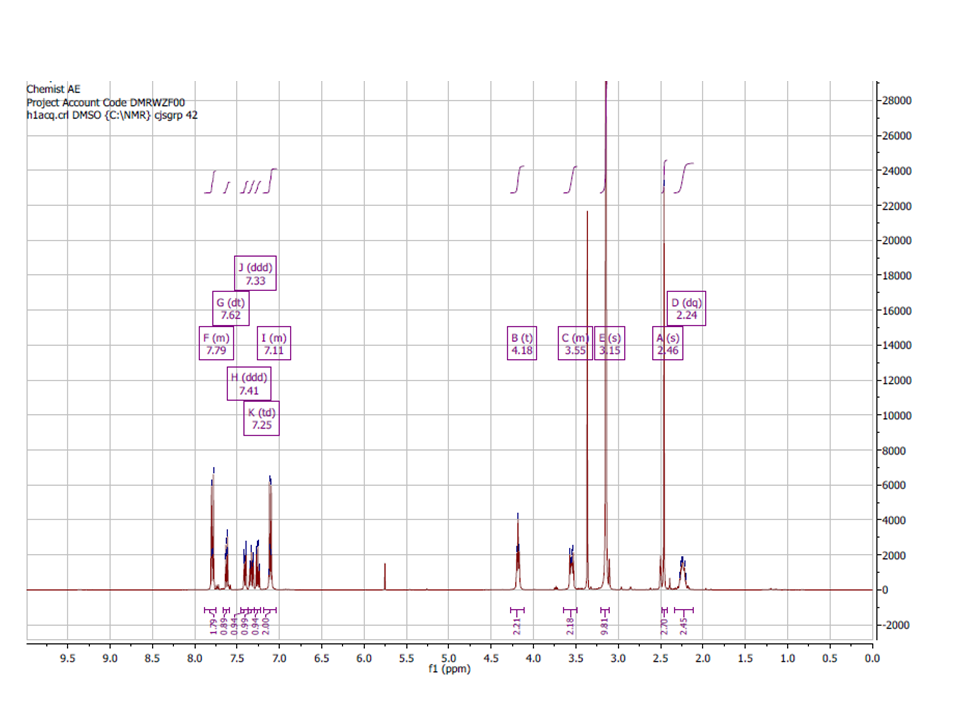


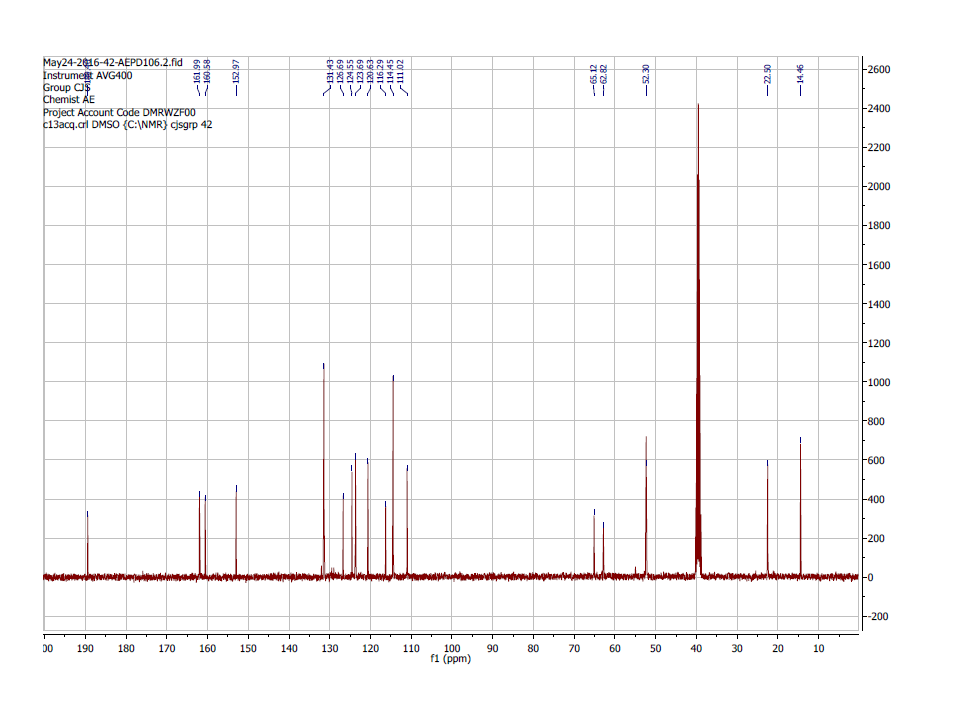


**(4-Methoxyphenyl)(1-methyl-1H-indol-3-yl)methanone**

Compound **5d** was prepared according to general procedure C using *N*-methylindole (0.1 mL; 1.5 mmol), 4-methoxybenzoyl chloride (255 mg; 1.5 mmol) and aluminium trichloride (400 mg; 3 mmol). Purification of the crude product by flash chromatography (0 to 10% of EtOAc in cyclohexane) gave a yellow oil (173 mg; 79%).

**^1^H NMR (400 MHz, CDCl_3_)** *δ*: 8.38 (1H; m; H6), 7.83 (2H; d; *J* = 8.0 Hz; H11,H12), 7.53 (1H; s; H1), 7.34 (3H; m; H3,H4,H5), 6.97 (2H; d; *J* = 8.0 Hz; H13,H14), 3.88 (3H; s; H16), 3.83 (3H; s; H17).

**^13^C NMR (100 MHz, CDCl_3_)** *δ*: 189.8 (C9), 162.3 (C15), 137.6 (C2), 137.2 (C1), 133.6 (C7), 131.0 (C11,C12), 127.4 (C10), 123.6 (C4), 122.7 (C5), 122.6 (C6), 115.7 (C8), 113.6 (C13,C14), 109.7 (C3), 55.5 (C16), 33.6 (C17).

**HRMS-ESI (m/z)**: exact mass calculated for C_17_H_15_O_2_NNa [M+Na]^+^: 288.09950, found: 288.09932.

**IR (Diamant ATR, cm^-1^):** 2949, 2822, 1763, 1596, 1242, 758.


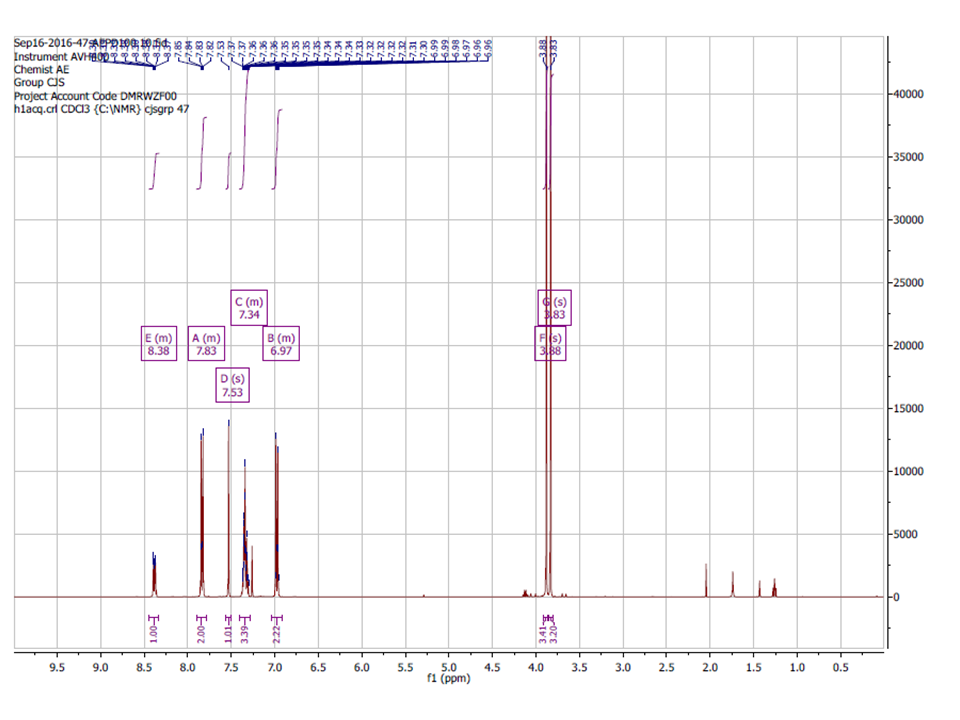


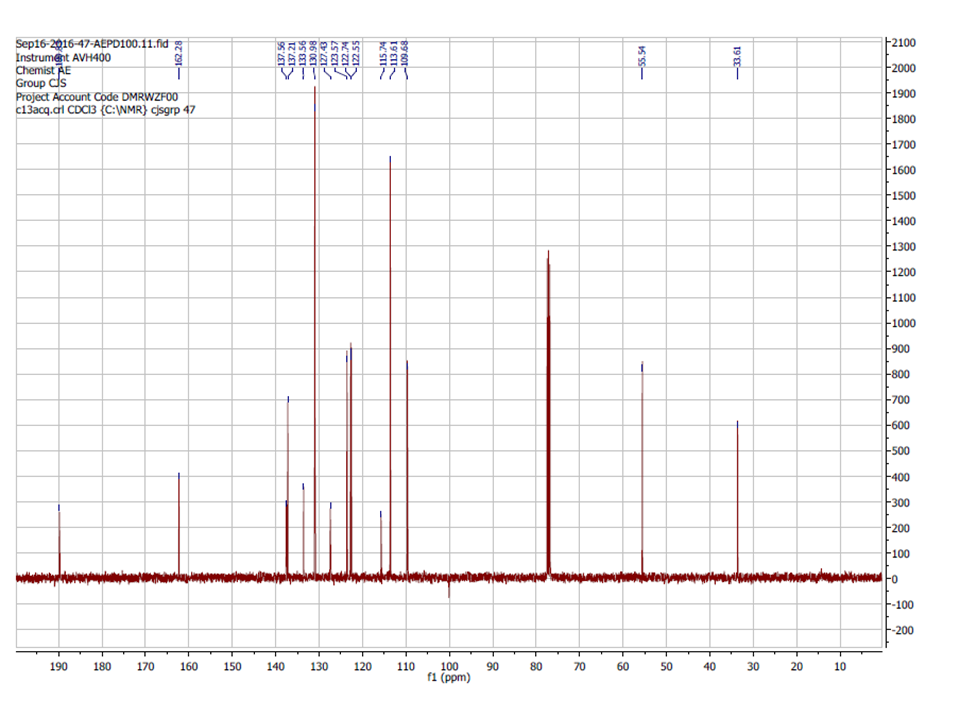


**(4-Hydroxyphenyl)(1-methyl-1H-indol-3-yl)methanone**

Compound **6d** was prepared according to general procedure D using compound **5d** (300 mg; 1.1 mmol) and aluminium trichloride (293 mg; 2.2 mmol). Purification of the crude product by flash chromatography (0 to 20% of EtOAc in cyclohexane) gave a yellow oil (121 mg; 43%).

**^1^H NMR (400 MHz, MeOD)** *δ*: 8.23 (1H, m; H6), 7.78 (1H; s; H1), 7.72 (2H; d; *J* = 8.0 Hz; H11,H12), 7.47 (1H; m; H3), 7.29 (2H; m; H4,H5), 6.91 (2H; d; *J* = 8.0 Hz; H13,H14), 3.86 (3H; s; H16).

**^13^C NMR (100 MHz, MeOD)** *δ*: 192.6 (C9), 162.3 (C15), 140.1 (C2), 139.2 (C1), 133.1 (C7), 132.3 (C11,C12), 128.6 (C10), 124.5 (C4), 123.4 (C5), 123.1 (C6), 116.1 (C8), 116.0 (C13,C14), 111.1 (C3), 33.7 (C16).

**HRMS-ESI (m/z)**: exact mass calculated for C_16_H_14_O_2_N [M+H]^+^: 252.10191, found: 252.10220.

**IR (Diamant ATR, cm^-1^):** 2938, 2805, 1757, 1586, 1232, 748.


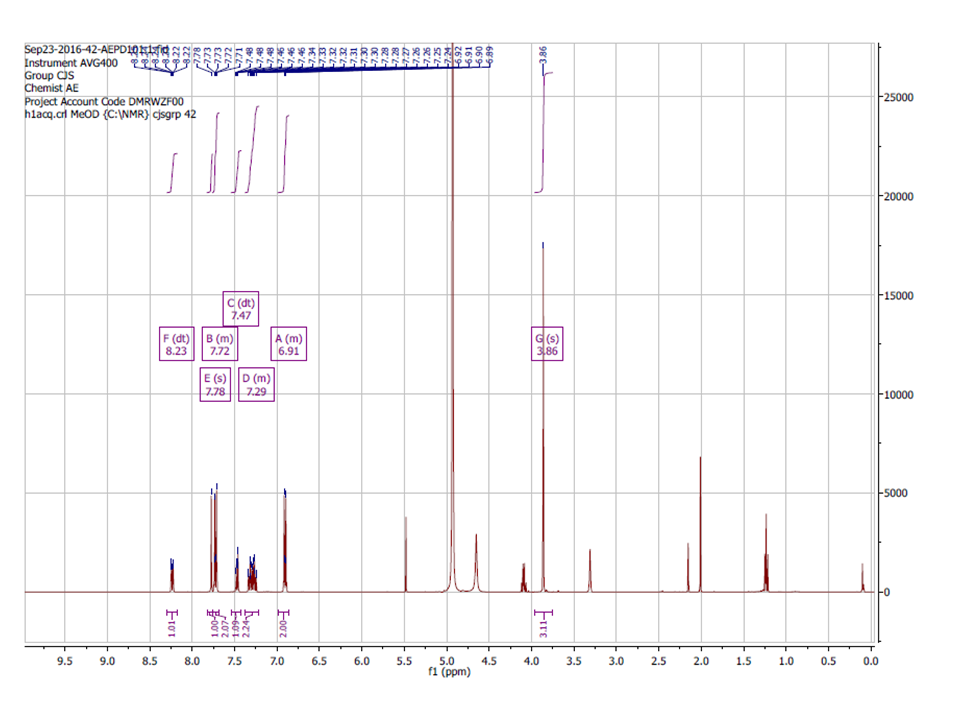


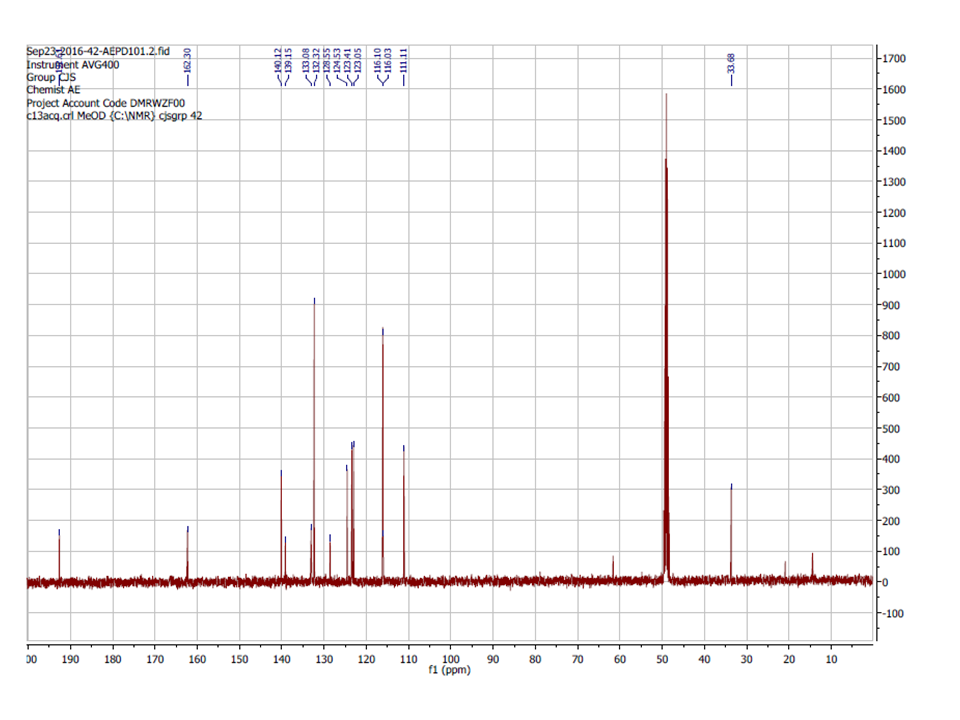


**(4-(3-(Dimethylamino)propoxy)phenyl)(1-methyl-1H-indol-3-yl)methanone**

Compound **1d** was prepared according to general procedure A using compound **6d** (121 mg; 0.48 mmol), 3-chloro-*N*,*N*-dimethylpropan-1-amine hydrochloride (439 mg; 2.78 mmol) and potassium carbonate (450 mg; 3.26 mmol). Purification of the crude product by flash chromatography (0 to 5% of MeOH in CH_2_Cl_2_) gave a yellow oil (204 mg; 96%).

**^1^H NMR (400 MHz, CDCl_3_)** *δ*: 8.36 (1H; m; H6), 7.82 (2H; d; *J* = 8.0 Hz; H11,H12), 7.55 (1H; s; H1), 7.34 (3H; m; H3,H4,H5), 6.97 (2H; d; *J* = 8.0 Hz; H13,H14), 4.11 (2H; m; H16), 3.85 (3H; s; H21), 2.56 (2H; m; H18), 2.33 (6H; s; H19,H20), 2.04 (2H; m; H17).

**^13^C NMR (100 MHz, CDCl_3_)** *δ*: 189.9 (C9), 161.7 (C15), 137.6 (C2), 137.3 (C1), 133.5 (C7), 131.0 (C11,C12), 127.4 (C10), 123.6 (C4), 122.8 (C5), 122.6 (C6), 115.8 (C8), 114.1 (C13,C14), 109.7 (C3), 66.3 (C16), 56.4 (C18), 45.4 (C19,C20), 33.7 (C21), 27.3 (C17).

**HRMS-ESI (m/z)**: exact mass calculated for C_21_H_25_N_2_O_2_ [M+H]^+^: 337.19105, found: 337.19056.

**IR (Diamant ATR, cm^-1^):** 2933, 2813, 1751, 1598, 1231, 742.


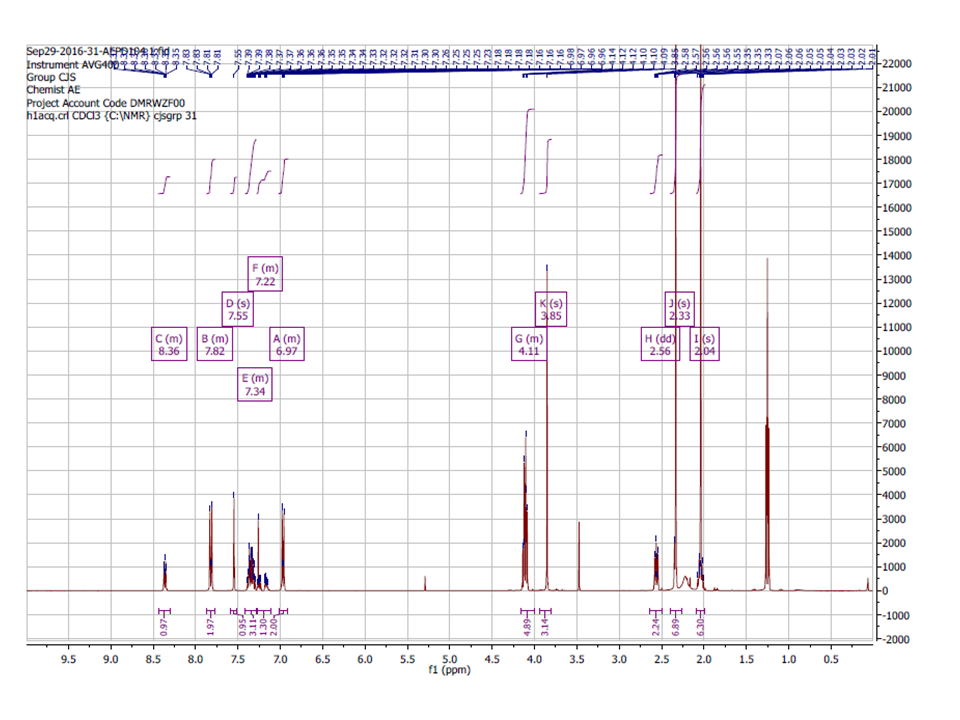


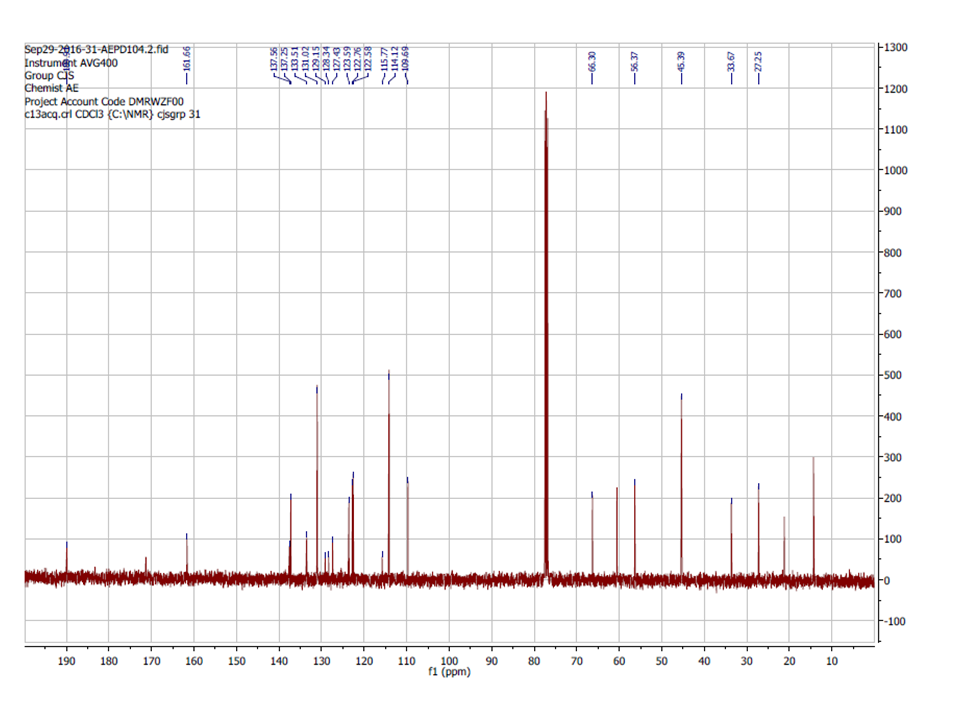


***N,N,N*-trimethyl-3-(4-(1-methyl-1H-indole-3-carbonyl)phenoxy)propan-1-aminium iodide**

Compound **2d** was prepared according to general procedure B using compound **1d** (200mg; 0.6 mmol) and iodomethane (110 µL; 1.8 mmol) to give a yellow powder (83 mg; 29%).

**^1^H NMR (400 MHz, DMSO)** *δ*: 8.24 (1H; m; H6), 8.02 (1H; s; H1), 7.82 (2H; m; H11,H12), 7.57 (1H; m; H3), 7.29 (2H; m; H4,H5), 7.10 (2H; m; H13,H14), 4.17 (2H; m; H16), 3.89 (3H; s; H22), 3.55 (2H; m; H18), 3.14 (9H; s; H19,H20,H21), 2.24 (2H; m; H17).

**^13^C NMR (100 MHz, DMSO)** *δ*: 188.2 (C9), 160.6 (C15), 138.6 (C2), 137.2 (C1), 133.2 (C7), 130.6 (C11,C12), 126.8 (C10), 123.1 (C4), 122.1 (C5), 121.6 (C6), 114.2 (C13,C14), 113.8 (C8), 110.6 (C3), 65.0 (C16), 62.9 (C18), 52.4 (C19,C20,C21), 33.2 (C22), 22.6 (C17).

**HRMS-ESI (m/z)**: exact mass calculated for C_22_H_27_O_2_N_2_ [M]^+^: 351.20670, found: 351.20667.

**IR (Diamant ATR, cm^-1^):** 3012, 2940, 1613, 1297, 774.

**Mp :** 183 °C.


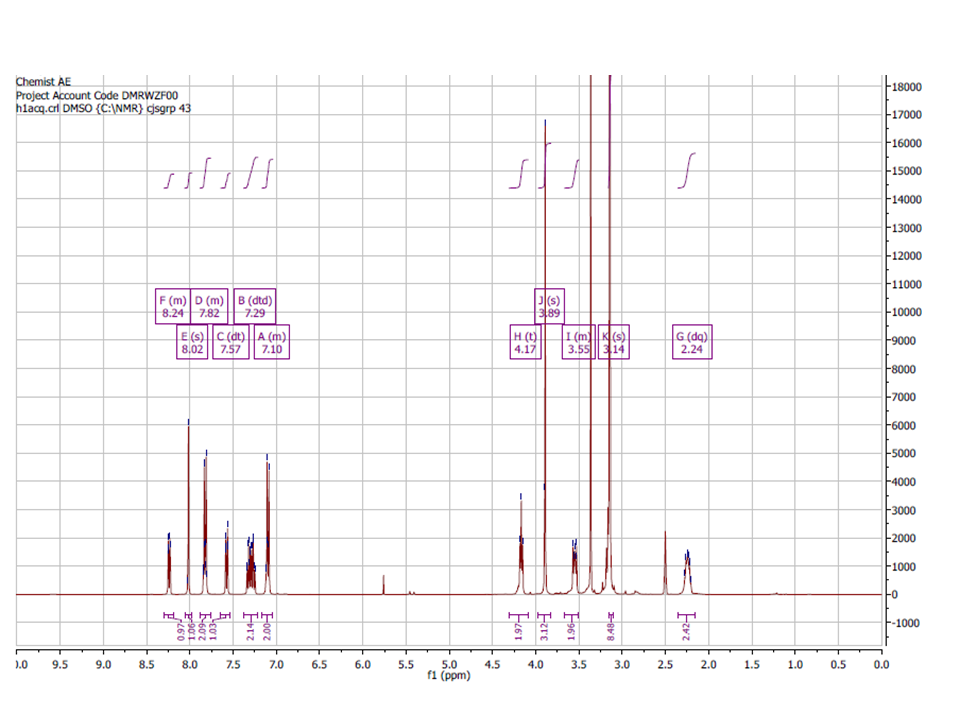


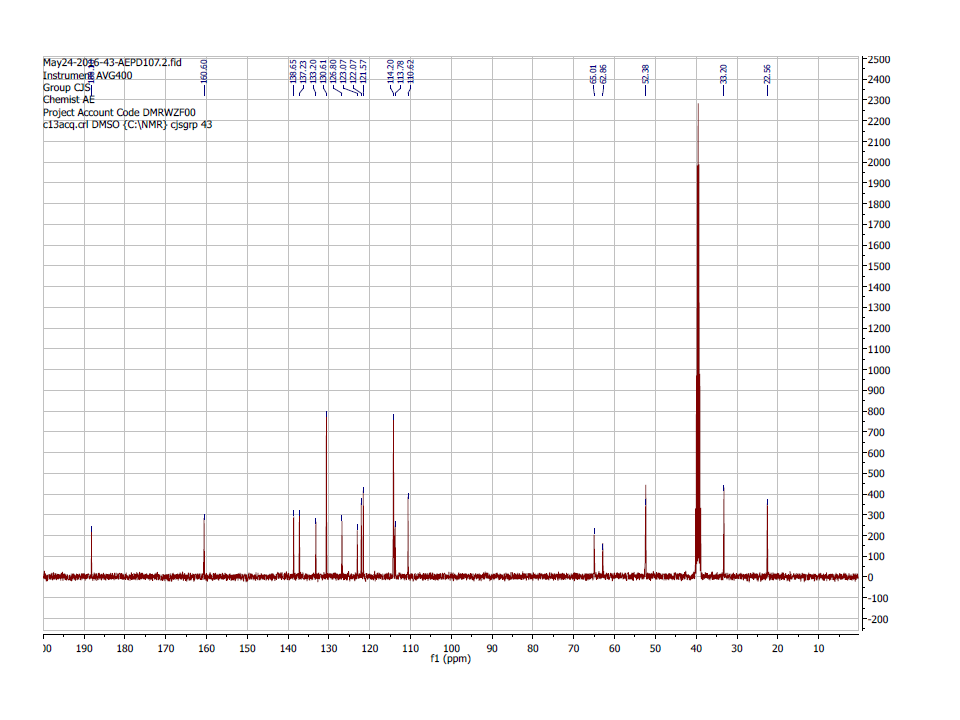


**(2-Butylbenzofuran-3-yl)(3,4,5-trimethoxyphenyl)methanone**

Compound **5-OMe** was prepared according to general procedure C using 2-butylbenzofuran (0.3 mL; 1.7 mmol), 3,,4,5-trimethoxybenzoyl chloride (391 mg; 1.7 mmol) and aluminium trichloride (445mg; 3.4 mmol). Purification of the crude product by flash chromatography (0 to 10% of EtOAc in cyclohexane) gave a colourless oil (52 mg; 8%).

**^1^H NMR (400 MHz, CDCl_3_)** *δ*: 7.72 (2H; m; H3,H6), 7.53 (1H; m; H5), 7.46 (1H; m; H4), 7.39 (2H; s; H11,H12), 4.21 (3H; s; H17), 4.09 (6H; s; H16,H18), 3.18 (2H; m; H19), 2.03 (2H; m; H20), 1.64 (2H; m; H21), 1.16 (3H; t; *J* = 7.0 Hz; H22).

**^13^C NMR (100 MHz, CDCl_3_)** *δ*: 190.7 (C9), 165.5 (C1), 153.6 (C2), 153.1 (C13,C14), 142.2 (C15), 134.3 (C10), 126.9 (C7), 124.4 (C4), 123.4 (C5), 121.4 (C6), 116.5 (C8), 111.1 (C3), 106.8 (C11,C12), 61.0 (C17), 56.3 (C16,C18), 30.3 (C19), 28.0 (C20), 22.5 (C21), 13.8 (C22).

**HRMS-ESI (m/z)**: exact mass calculated for C_22_H_25_O_5_ [M+H]^+^: 369.16965, found: 369.16930.

**IR (Diamant ATR, cm^-1^):** 2958, 2872, 1581, 1101, 736.


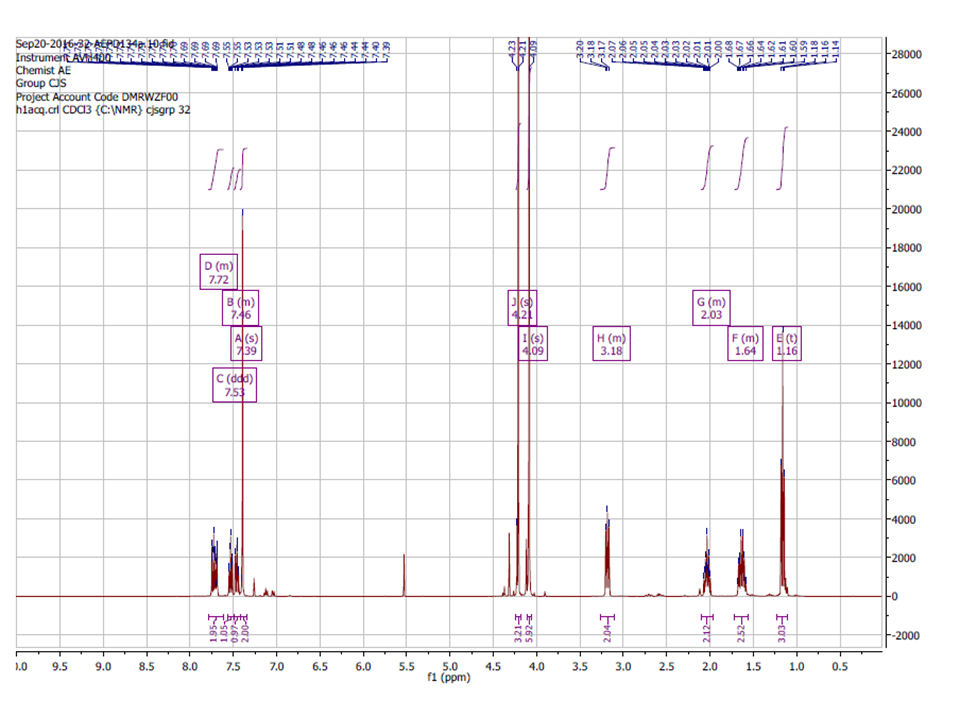


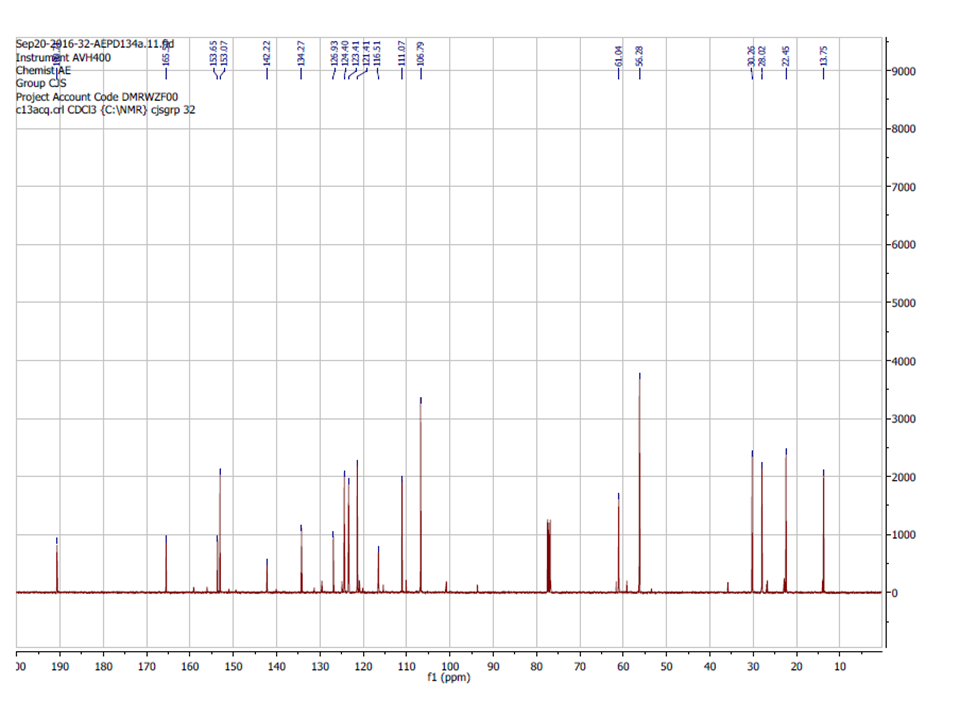


**(2-Butylbenzofuran-3-yl)(4-hydroxy-3,5-dimethoxyphenyl)methanone**

Compound **6-OMe** was prepared according to general procedure D using compound **5-OMe** (610 mg; 1 mmol) and aluminium trichloride (278 mg; 2 mmol). Purification of the crude product by flash chromatography (0 to 20% of EtOAc in cyclohexane) gave a yellow powder (401 mg; 67%).

**^1^H NMR (400 MHz, CDCl_3_)** *δ*: 7.47 (1H; m; H6), 7.41 (1H; m; H3), 7.26 (1H; m; H5), 7.19 (2H + 1H; m; H4,H11,H12), 3.85 (6H; s; H16,H17), 2.92 (2H; m; H18), 1.76 (2H; m; H19), 1.36 (2H; m; H20), 0.89 (3H; t; *J* = 7.0 Hz; H21).

**^13^C NMR (100 MHz, CDCl_3_)** *δ*: 190.5 (C9), 165.0 (C2), 153.7 (C1), 146.8 (C13,C14), 139.6 (C15), 130.3 (C10), 127.1 (C7), 124.4 (C4), 123.4 (C5), 121.4 (C6), 116.6 (C8), 111.1 (C3), 106.9 (C11,C12), 56.5 (C16,C17), 30.3 (C18), 28.0 (C19), 22.5 (C20), 13.8 (C21).

**HRMS-ESI (m/z)**: exact mass calculated for C_21_H_23_O_5_ [M+H]^+^: 355.15400, found: 355.15411.

**IR (Diamant ATR, cm^-1^):** 3542, 2960, 2933, 2872, 1639, 1148, 750.

**Mp :** 78 °C.


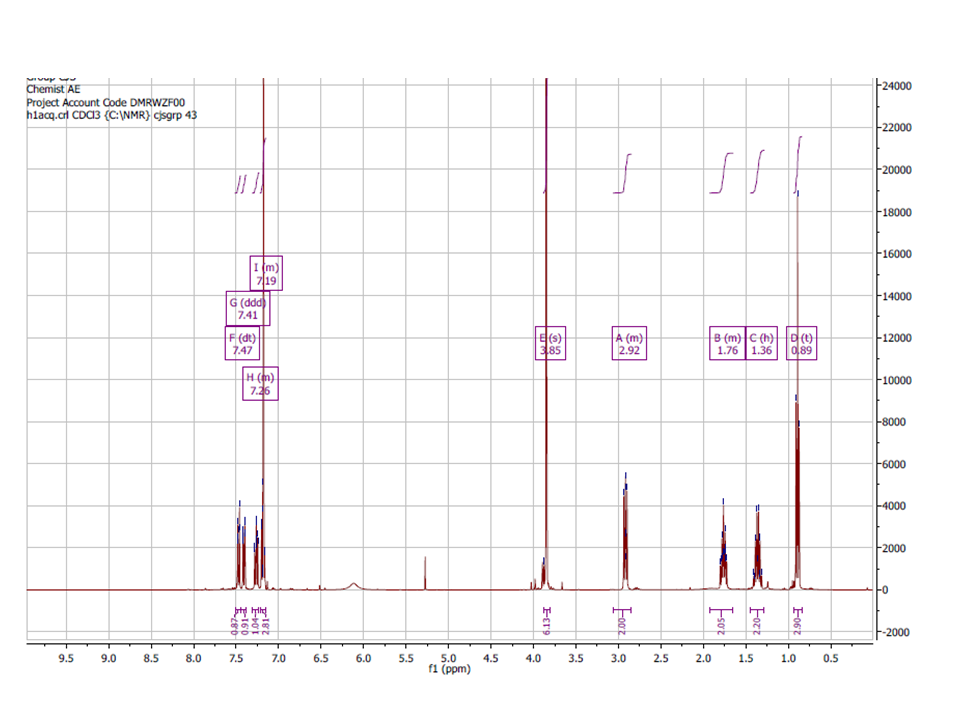


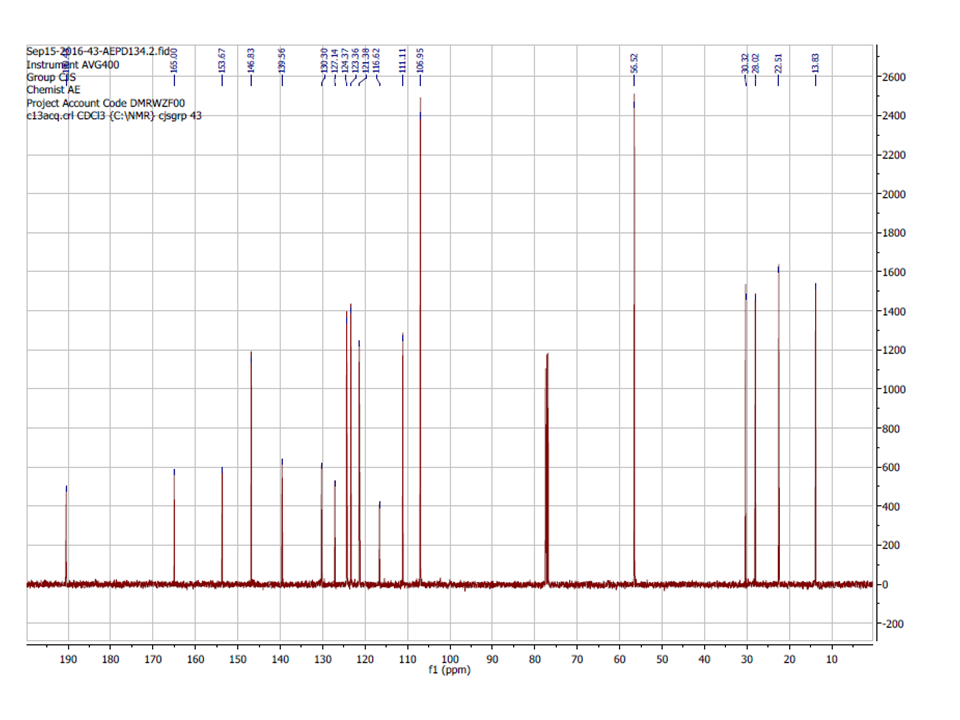


**(2-Butylbenzofuran-3-yl)(4-(3-(dimethylamino)propoxy)-3,5-dimethoxyphenyl)methanone**

Compound **1-OMe** was prepared according to general procedure A using compound **6-OMe** (400 mg; 1.13 mmol), 3-chloro-*N*,*N*-dimethylpropan-1-amine hydrochloride (982 mg; 6.2 mmol) and potassium carbonate (1.1 g; 7.68 mmol). Purified by flash chromatography (0 to 15 % of MeOH in CH_2_Cl_2_) gave a yellow oil (123 mg; 25%).

**^1^H NMR (400 MHz, CDCl_3_)** *δ*: 7.47 (1H; m; H6), 7.41 (1H; m; H3), 7.26 (1H; m; H5), 7.18 (1H; m; H4), 7.10 (2H; s; H11,H12), 4.13 (2H; m; H16), 3.80 (6H; s; H21,H22), 2.90 (2H; m; H18), 2.54 (2H; m; H17), 2.27 (6H; s; H19,H20), 1.97 (2H; m; H23), 1.75 (2H; m; H24), 1.36 (2H; m; H25), 0.89 (3H; t; *J* = 7.0 Hz; H26).

**^13^C NMR (100 MHz, CDCl_3_)** *δ*: 190.8 (C9), 165.5 (C2), 153.7 (C1), 153.3 (C13,C14), 141.4 (C15), 134.3 (C10), 127.0 (C7), 124.4 (C4), 123.4 (C5), 121.4 (C6), 116.6 (C8), 111.1 (C3), 106.8 (C11,C12), 72.0 (C16), 56.5 (C18,C21,C22), 45.5 (C19,C20), 30.3 (C23), 28.3 (C17), 28.1 (C24), 22.5 (C25), 13.8 (C26).

**HRMS-ESI (m/z)**: exact mass calculated for C_26_H_34_O_5_N [M+H]^+^: 440.24315, found: 440.24133.

**IR (Diamant ATR, cm^-1^):** 3544, 2972, 2942, 2869, 1645, 1136, 778.


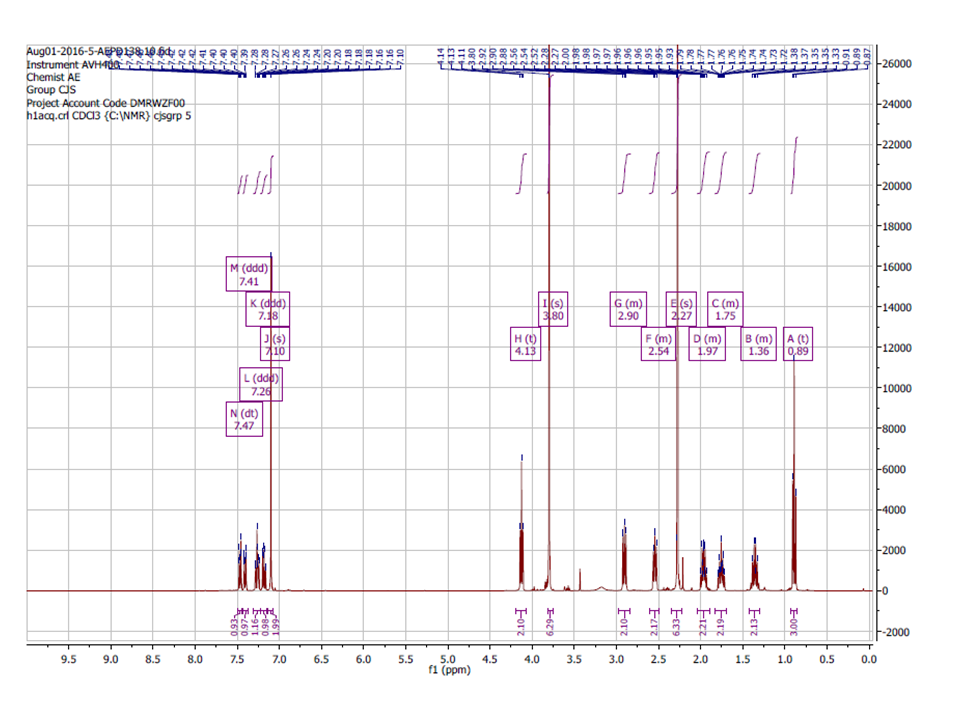


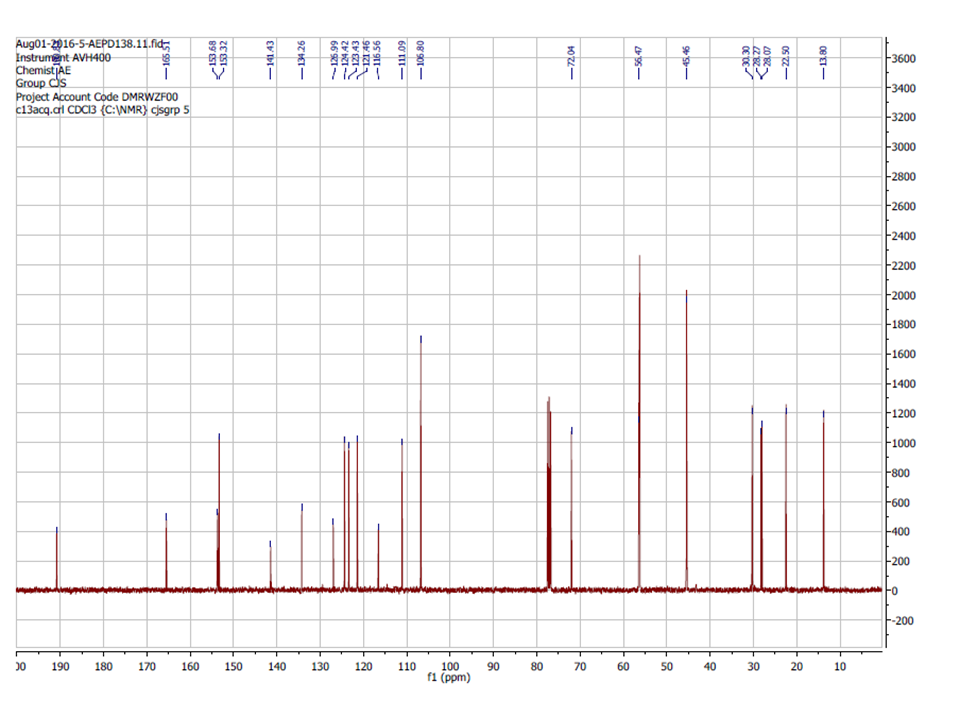


**3-(4-(2-Butylbenzofuran-3-carbonyl)-2,6-dimethoxyphenoxy)-N,N,N-trimethylpropan-1-aminium iodide**

Compound **8a** was prepared according to general procedure B using compound **1-OMe** (123 mg; 0.28 mmol) and iodomethane (60 µL; 0.84 mmol) to give a yellow powder (160 mg; 98%).

**^1^H NMR (400 MHz, CDCl_3_)** *δ*: 7.46 (1H; m; H6), 7.36 (1H; m; H3), 7.25 (1H; m; H5), 7.18 (1H; m; H4), 7.07 (2H; s; H11,H12), 4.16 (2H; m; H16), 3.92 (2H; m; H18), 3.80 (6H; s; H22,H23), 3.51 (9H; s; H19,H20,H21), 2.90 (2H; m; H24), 2.28 (2H; m; H17), 1.74 (2H; m; H25), 1.34 (2H; m; H26), 0.88 (3H; t; *J* = 7.5 Hz; H27).

**^13^C NMR (100 MHz, CDCl_3_)** *δ*: 190.5 (C9), 165.8 (C2), 153.6 (C1), 152.9 (C13,C14), 140.1 (C15), 134.9 (C10), 126.8 (C7), 124.5 (C4), 123.5 (C5), 121.3 (C6), 116.4 (C8), 111.2 (C3), 106.5 (C11,C12), 69.2 (C16), 65.2 (C18), 56.4 (C22,C23), 54.1 (C19,C20,C21), 30.3 (C24), 28.1 (C17), 24.6 (C25), 22.5 (C26), 13.8 (C27).

**HRMS-ESI (m/z)**: exact mass calculated for C_27_H_36_O_5_N [M]^+^: 454.25880, found: 454.25833.

**IR (Diamant ATR, cm^-1^):** 2926, 2864, 1643, 1101, 715.

**Mp :** 145 °C.


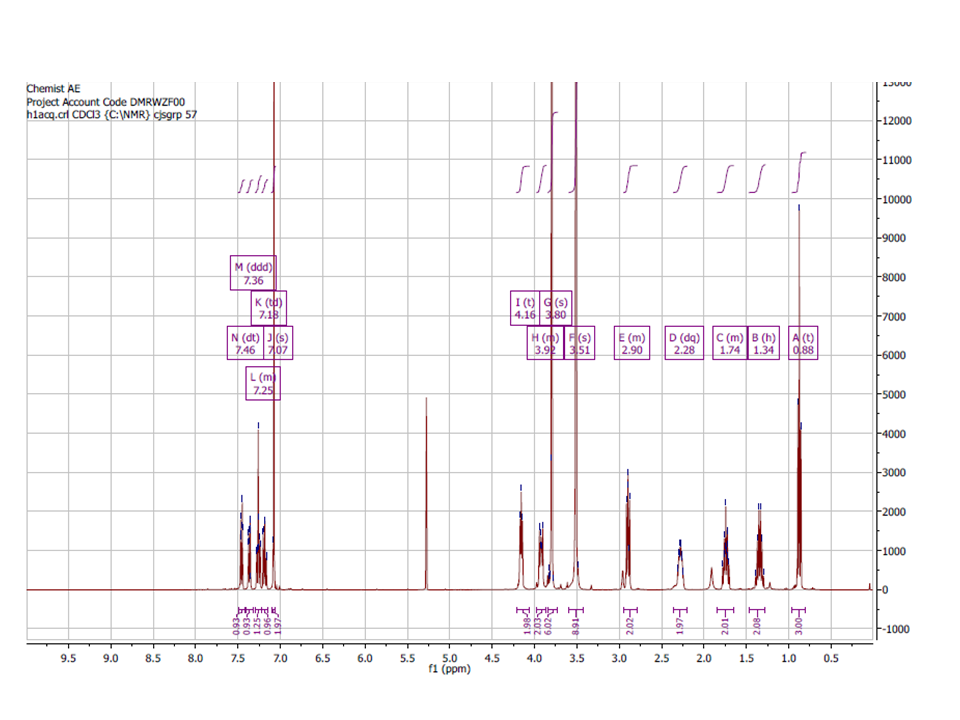


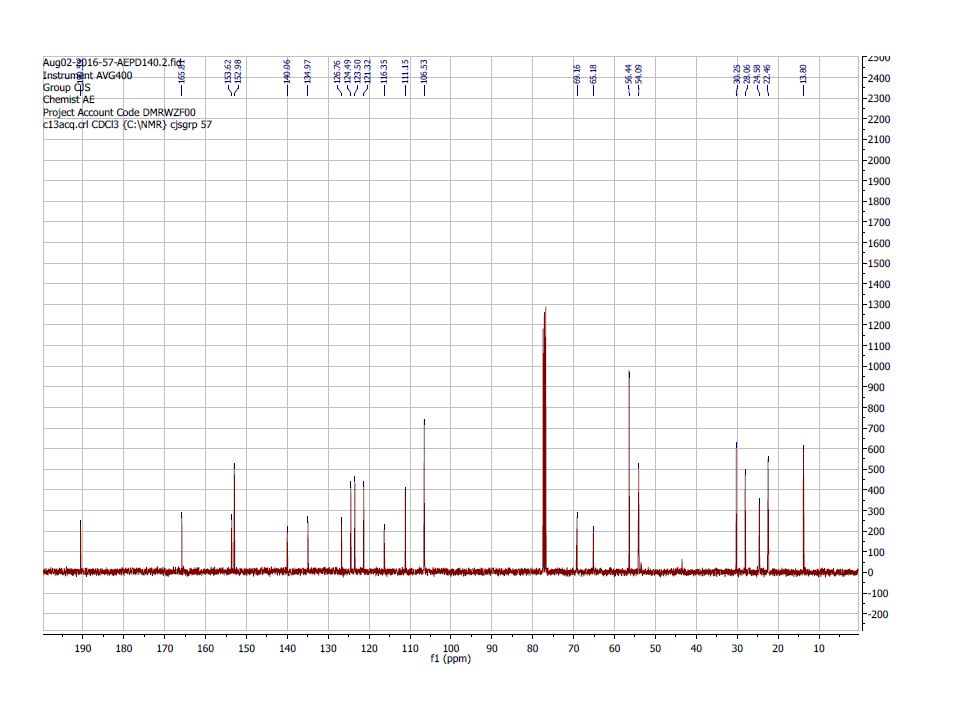


**Tert-butyl (3-(4-(2-butylbenzofuran-3-carbonyl)phenoxy)propyl)carbamate**

Potassium carbonate (328 mg; 2.38 mmol) was added to a solution of the (2-butylbenzofuran-3-yl)(4-hydroxyphenyl)methanone (100 mg; 0.34 mmol) in 3 mL of DMF and the mixture was heated until 60°C under stirring. Then, *N*-boc-3-bromopropylamine (170 mg; 0.68 mmol) was added and the temperature was increased to 120 °C. The consumption of the (2-butylbenzofuran-3-yl)(4-hydroxyphenyl)methanone was monitored by TLC and the reaction was quenched with 90 mL of water. The product was extracted with 90 mL of EtOAc, washed with brine and dried with Na_2_SO_4_. After filtration, the solvent was evaporated and the purification of the crude product by flash chromatography (0 to 20 % of EtOAc in cyclohexane) gave compound **9** as a colourless oil (145 mg; 94%).

**^1^H NMR (400 MHz, CDCl_3_)** *δ*: 7.82 (2H; m; H11,H12), 7.46 (1H; m; H6), 7.34 (1H; m; H3), 7.25 (1H; m; H4), 7.16 (1H; m; H5), 6.93 (2H; m; H13,H14), 4.85 (1H; br s; NH), 4.09 (2H; m; H17), 3.34 (2H; m; H16), 2.90 (2H; m; H24), 2.01 (2H; m; H18), 1.74 (2H; m; H25), 1.43 (9H; s; H21,H22,H23), 1.34 (2H; m; H26), 0.88 (3H; t; *J* = 7.5 Hz; H27).

**^13^C NMR (100 MHz, CDCl_3_)** *δ*: 190.5 (C9), 164.7 (C2), 162.7 (C15), 156.1 (C1), 153.6 (C19), 131.9 (C10), 131.8 (C11,C12), 127.2 (C7), 124.2 (C4), 123.4 (C5), 121.3 (C6), 116.8 (C8), 114.2 (C13,C14), 111.0 (C3), 79.4 (C20), 66.1 (C16), 37.9 (C18), 30.2 (C24), 29.6 (C17), 28.5 (C21,C22,C23), 27.9 (C25), 22.4 (C26), 13.8 (C27).

**HRMS-ESI (m/z)**: exact mass calculated for C_27_H_34_O_5_N [M+H]^+^: 452.24315, found: 352.24259.

**IR (Diamant ATR, cm^-1^):** 3430, 2977, 2958, 2873, 1600, 1241, 1126, 749.


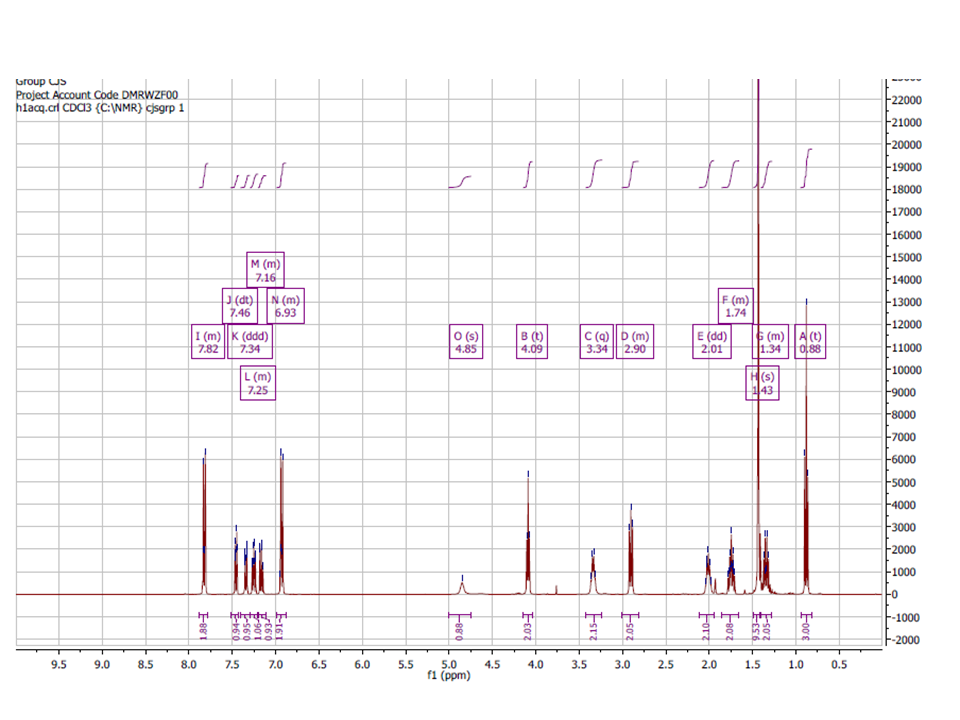


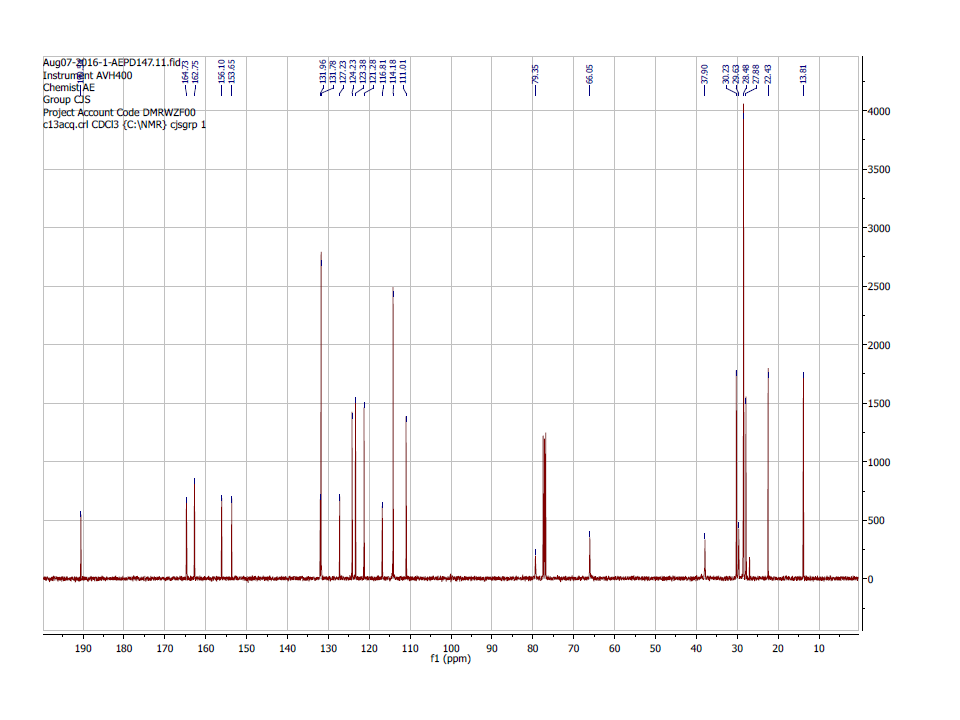


**(4-(3-Aminopropoxy)phenyl)(2-butylbenzofuran-3-yl)methanone**

Compound **9** (145 mg; 0.32 mmol) was dissolved in 4 mL of CH_2_Cl_2_ and 1 mL of TFA was added at room temperature under stirring for 1 hour. The solvent was evaporated and the purification of the crude product by flash chromatography (0 to 5 % of MeOH in dichloromethane) gave compound **10** as a yellow oil (102 mg; 91%).

**^1^H NMR (400 MHz, CDCl_3_)** *δ*: 7.73 (2H; m; H11,H12), 7.43 (1H; m; H6), 7.26 (2H; m; H3,H5), 7.12 (1H; m; H4), 6.88 (2H; m; H13,H14), 6.77 (2H; br s; NH2), 4.09 (2H; m; H17), 3.19 (2H; m; H16), 2.85 (2H; m; H19), 2.16 (2H; m; H18), 1.70 (2H; m; H20), 1.28 (2H; m; H21), 0.84 (3H; t; *J* = 7.5 Hz; H22).

**^13^C NMR (100 MHz, CDCl_3_)** *δ*: 191.1 (C9), 165.3 (C1), 162.2 (C15), 153.7 (C2), 132.4 (C10), 131.6 (C11,C12), 127.1 (C7), 124.4 (C4), 123.5 (C5), 121.3 (C6), 116.7 (C8), 114.3 (C13,C14), 111.1 (C3), 65.5 (C16), 38.2 (C18), 30.2 (C19), 27.9 (C20), 27.3 (C17), 22.5 (C21), 13.8 (C22).

**HRMS-ESI (m/z)**: exact mass calculated for C_22_H_26_O_3_N [M+H]^+^: 352.19072, found: 352.19067.

**IR (Diamant ATR, cm^-1^):** 2959, 2930, 1670, 1640, 1205, 722.


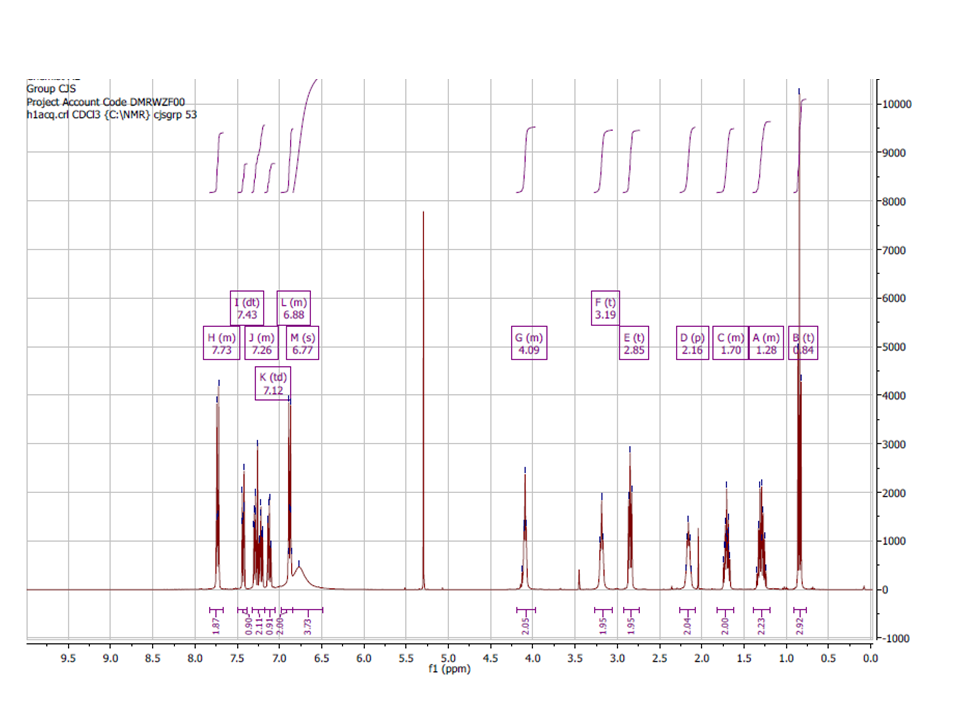


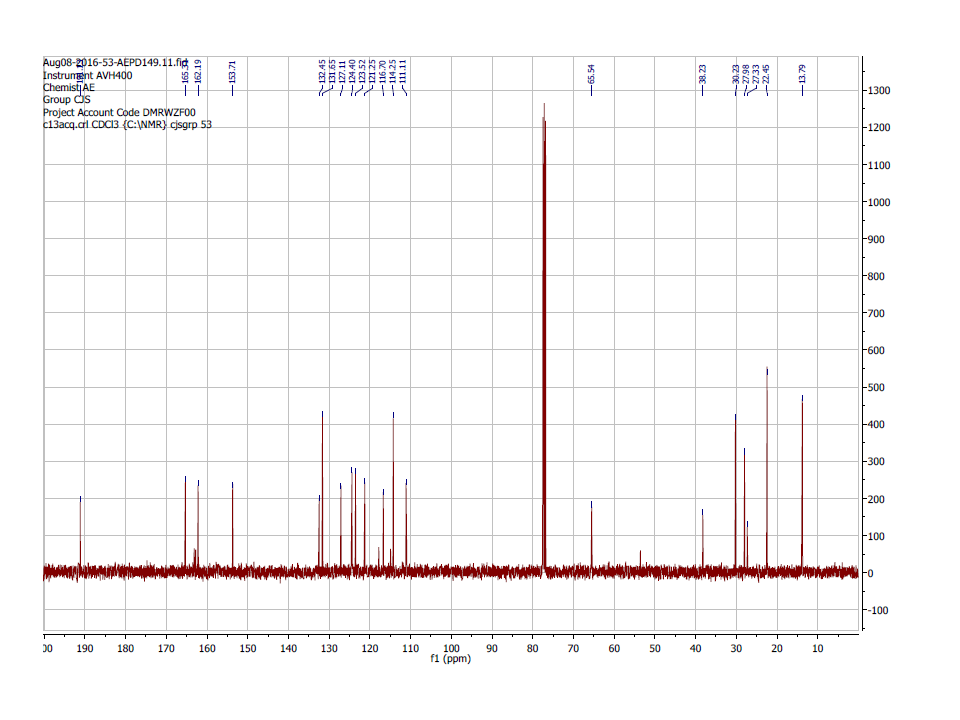


**(2-Butylbenzofuran-3-yl)(3,5-difluoro-4-methoxyphenyl)methanone**

Compound **5-F** was prepared according to general procedure C using 2-butylbenzofuran (0.3 mL; 1.7 mmol), 3,5-difluoro-4-methoxybenzoyl chloride (0.35 mL; 1.7 mmol) and aluminium trichloride (445 mg; 3.4 mmol). Purification of the crude product by flash chromatography (0 to 10% of EtOAc in cyclohexane) gave a colourless oil (360 mg; 61%).

**^1^H NMR (400 MHz, CDCl_3_)** *δ*: 7.41 (3H; m; H6,H11,H12), 7.31 (1H; m; H3), 7.22 (2H; m; H4,H5), 4.09 (3H; s; H16), 2.87 (2H; m; H17), 1.72 (2H; m; H18), 1.33 (2H; m; H19), 0.87 (3H; t; *J* = 7.0 Hz; H20).

**^13^C NMR (100 MHz, CDCl_3_)** *δ*: 188.6 (C9), 165.9 (C1), 156.2 (C2), 153.8 (C10), 140.6 (C7), 133.1 (C4), 126.6 (C5), 124.7 (C3,C6), 122.0 (J = 240 Hz; C13,C14), 116.0 (C8), 113.7 (J = 7 Hz; C15), 111.3 (C11,C12), 61.9 (C16), 30.2 (C17), 28.1 (C18), 22.5 (C19), 13.8 (C20).

**HRMS-ESI (m/z)**: exact mass calculated for C_20_H_19_O_3_F_2_ [M+H]^+^: 345.12968, found: 345.12979.

**IR (Diamant ATR, cm^-1^):** 3010, 2985, 1696, 1202, 972, 732.


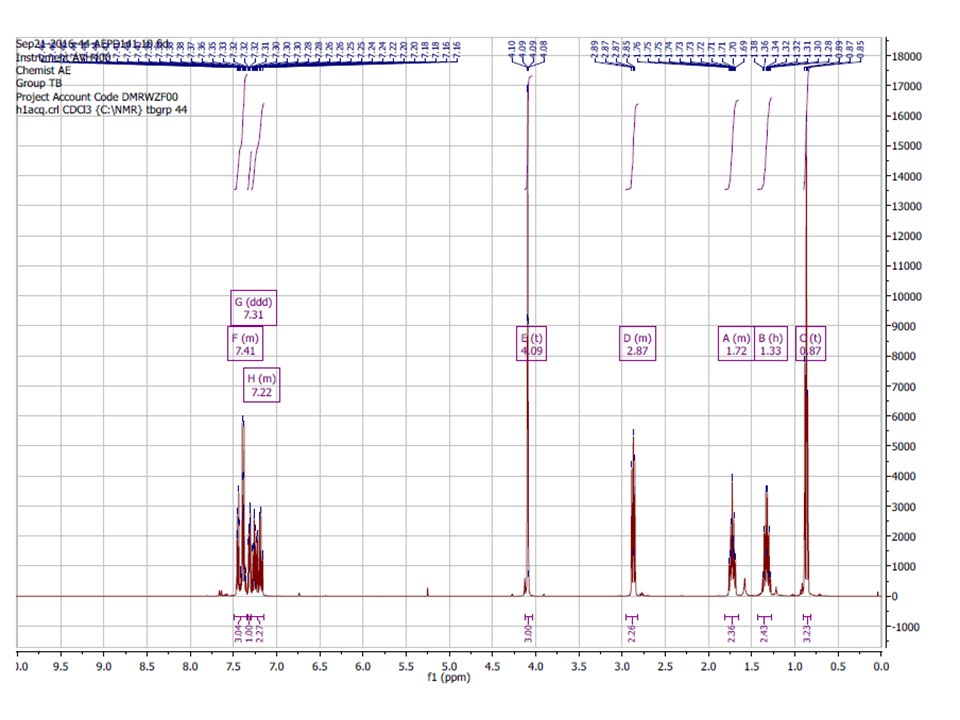


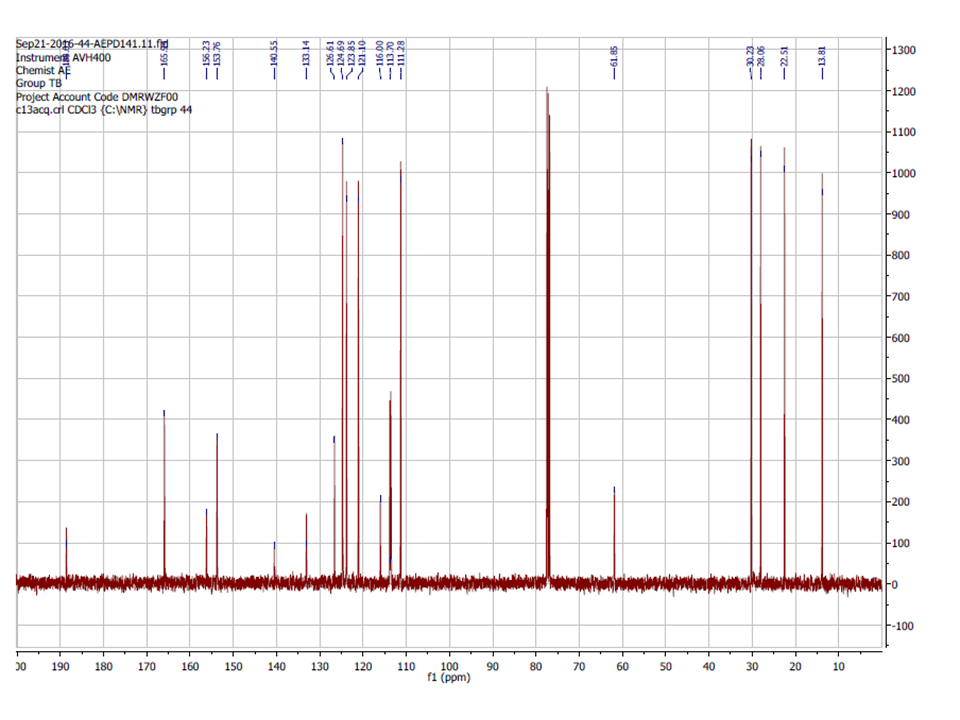


**(2-Butylbenzofuran-3-yl)(3,5-difluoro-4-hydroxyphenyl)methanone**

Compound **6-F** was prepared according to general procedure D using compound **5-F** (360 mg; 1.05 mmol) and aluminium trichloride (274 mg; 2.1 mmol). Purification of the crude product by flash chromatography (0 to 20% of EtOAc in cyclohexane) gave a yellow oil (340 mg; 98%).

**^1^H NMR (400 MHz, CDCl_3_)** *δ*: 7.47 (3H; m; H6,H11,H12), 7.35 (1H; m; H3), 7.29 (1H; m; H4), 7.21 (1H; m; H5), 2.91 (2H; m; H16), 1.76 (2H; m; H17), 1.36 (2H; m; H18), 0.90 (3H; t; *J* = 7.0 Hz; H19).

**^13^C NMR (100 MHz, CDCl_3_)** *δ*: 189.2 (C9), 165.8 (C1), 153.7 (C2), 150.4 (C10), 138.1 (C7), 130.1 (C4), 126.6 (C5), 124.7 (C3,C6), 122.0 (J = 240 Hz; C13,C14), 116.0 (C8), 113.3 (J = 7 Hz; C15), 111.3 (C11,C12), 30.2 (C16), 28.0 (C17), 22.5 (C18), 13.8 (C19).

**HRMS-ESI (m/z)**: exact mass calculated for C_19_H_17_O_3_F_2_ [M+H]^+^: 331.11403, found: 331.11400.

**IR (Diamant ATR, cm^-1^):** 3002, 2956, 1669, 1226, 952, 742.


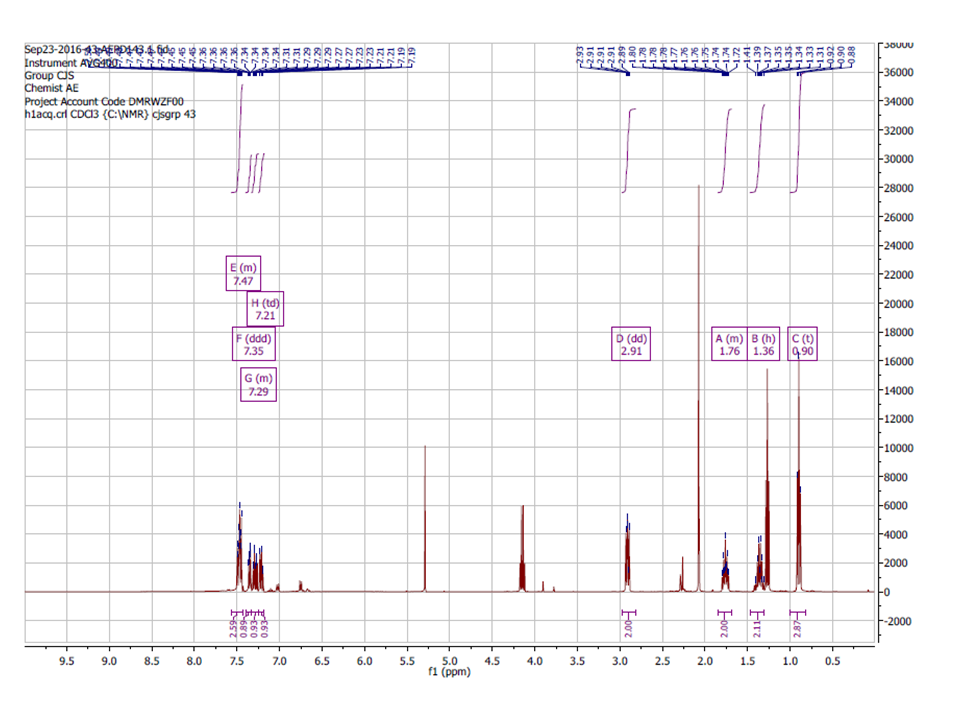


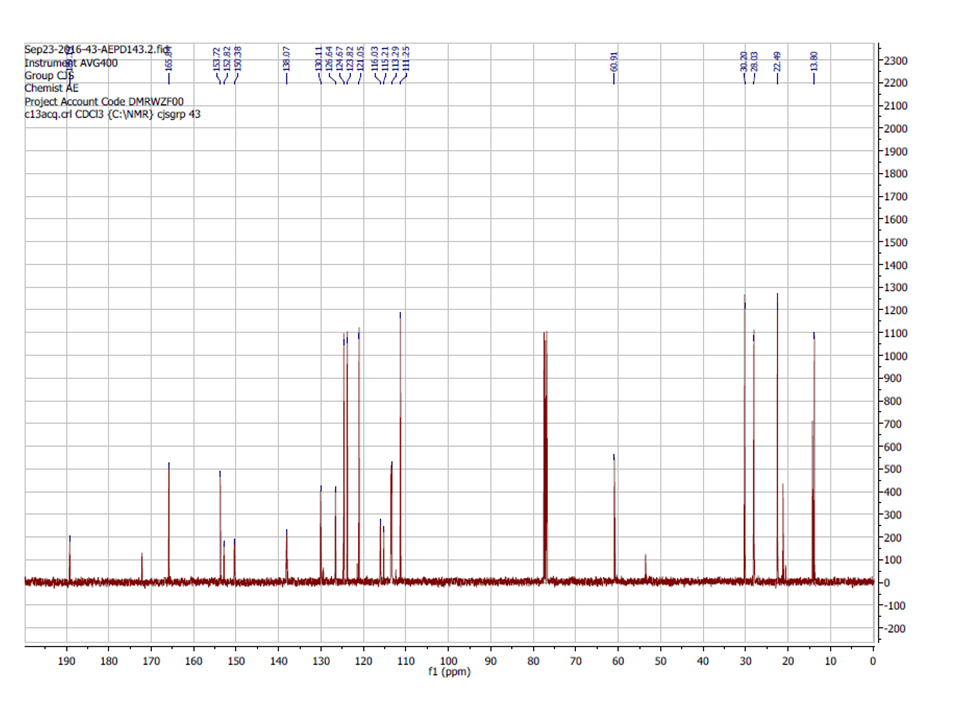


**(2-Butylbenzofuran-3-yl)(4-(3-(dimethylamino)propoxy)-3,5-difluorophenyl)methanone**

Compound **1-F** was prepared according to general procedure A using compound **6-F** (340 mg; 1.03 mmol), 3-chloro-*N*,*N*-dimethylpropan-1-amine hydrochloride (895 mg; 5.66 mmol) and potassium carbonate (967 mg; 7 mmol). Purification of the crude product by flash chromatography (0 to 15 % of MeOH in CH_2_Cl_2_) gave a yellow oil (302 mg; 71%).

**^1^H NMR (400 MHz, CDCl_3_)** *δ*: 7.45 (1H; m; H5), 7.40 (2H; m; H11,H12), 7.33 (1H; m; H4), 7.26 (1H; m; H6), 7.18 (1H; m; H3), 4.33 (2H; m; H17), 2.89 (2H; m; H18), 2.48 (2H; m; H21), 2.23 (6H; s; H19,H20), 1.96 (2H; m; H16), 1.74 (2H; m; H22), 1.34 (2H; m; H23), 0.88 (3H; t; *J* = 7.0 Hz; H24).

**^13^C NMR (100 MHz, CDCl_3_)** *δ*: 188.5 (C9), 165.8 (C1), 156.6 (C2), 153.7 (C10), 139.9 (C7), 133.1 (C4), 126.5 (C5), 124.6 (C3,C6), 122.0 (J = 240 Hz; C13,C14), 115.9 (C8), 113.5 (J = 7 Hz; C15), 111.2 (C11,C12), 73.0 (C16), 55.9 (C18), 45.5 (C19,C20), 30.2 (C21), 28.3 (C22), 28.0 (C17), 22.4 (C23), 13.7 (C24).

**HRMS-ESI (m/z)**: exact mass calculated for C_24_H_28_O_3_NF_2_ [M+H]^+^: 416.20318, found: 416.20165.

**IR (Diamant ATR, cm^-1^):** 3009, 2945, 1678, 1232, 945, 772.


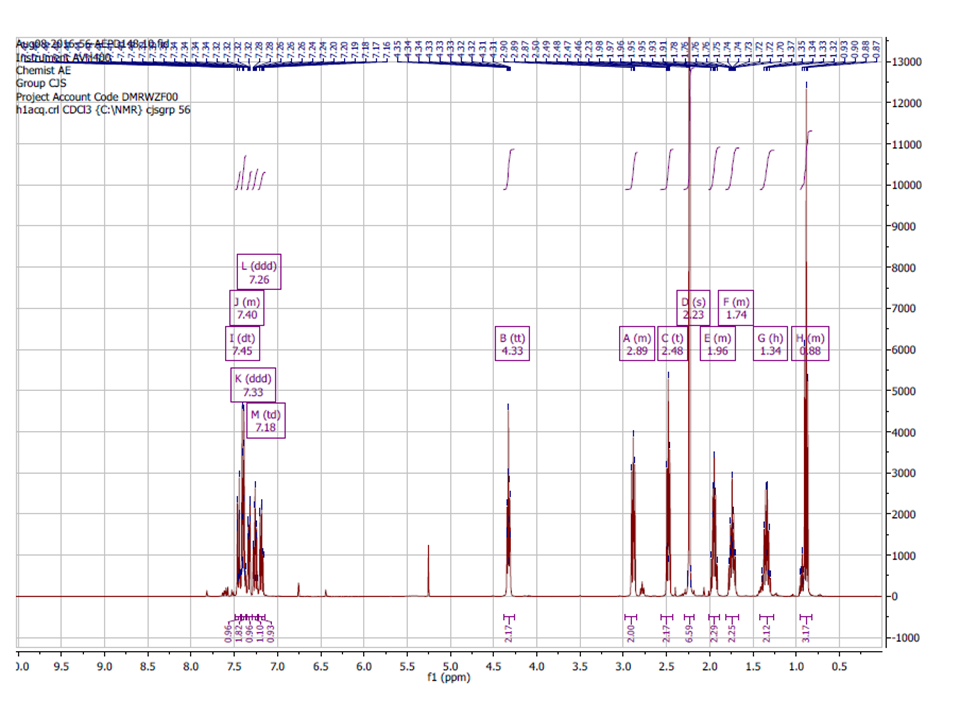


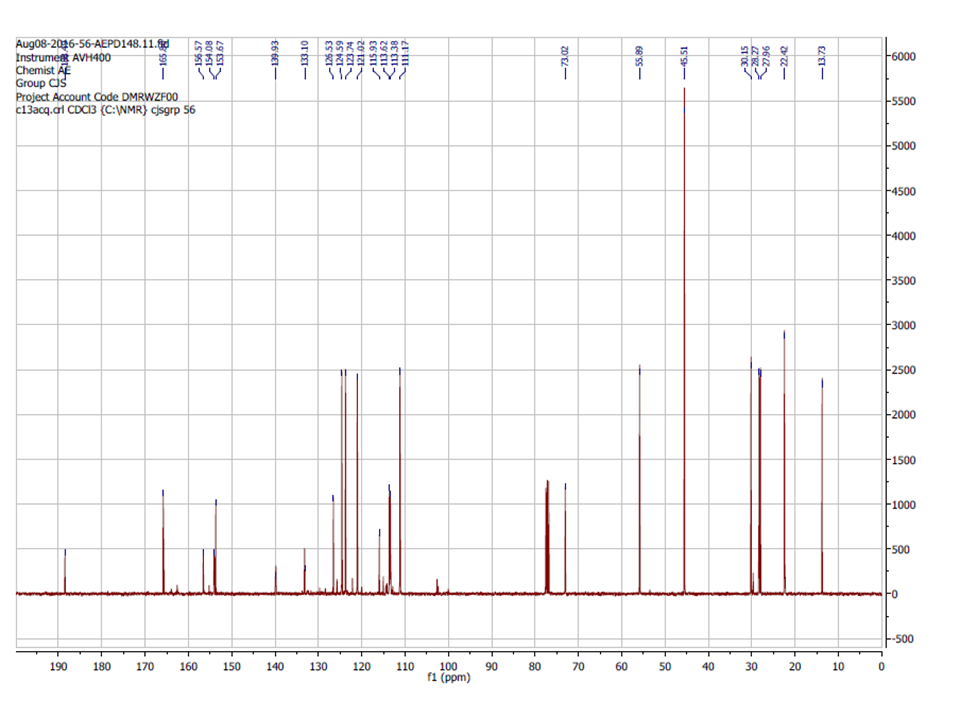


**3-(4-(2-Butylbenzofuran-3-carbonyl)-2,6-difluorophenoxy)-N,N,N-trimethylpropan-1-aminium iodide**

Compound **8b** was prepared according to general procedure B using compound **1-F** (300 mg; 0.72 mmol) and iodomethane (0.14 mL; 2.16 mmol) to give a yellow powder (360 mg; 89%).

**^1^H NMR (400 MHz, CDCl_3_)** *δ*: 7.32 (1H; m; H4), 7.26 (2H; m; H11,H12), 7.15 (3H; m; H5,H3,H6), 4.32 (2H; m; H17), 3.83 (2H; m; H18), 3.44 (9H; s; H19,H20,H21), 2.77 (2H; m; H22), 2.31 (2H; m; H16), 1.63 (2H; m; H23), 1.23 (2H; m; H24), 0.77 (3H; t; *J* = 7.5 Hz; H25).

**^13^C NMR (100 MHz, CDCl_3_)** *δ*: 187.9 (C9), 165.8 (C1), 155.9 (C2), 153.2 (C10), 138.3 (C7), 133.8 (C4), 125.9 (C5), 124.3 (C3,C6), 121.5 (J = 240 Hz; C13,C14), 115.3 (C8), 113.2 (J = 7 Hz; C15), 110.8 (C11,C12), 70.4 (C16), 63.9 (C18), 29.7 (C22), 29.5 (C17), 27.6 (C19,C20,C21), 24.2 (C23), 21.9 (C24), 13.4 (C25).

**HRMS-ESI (m/z)**: exact mass calculated for C_25_H_30_O_3_NF_2_ [M]^+^: 430.21883, found: 430.21836.

**IR (Diamant ATR, cm^-1^):** 3007, 2957, 1662, 1218, 961, 748.

**Mp :** 133 °C.


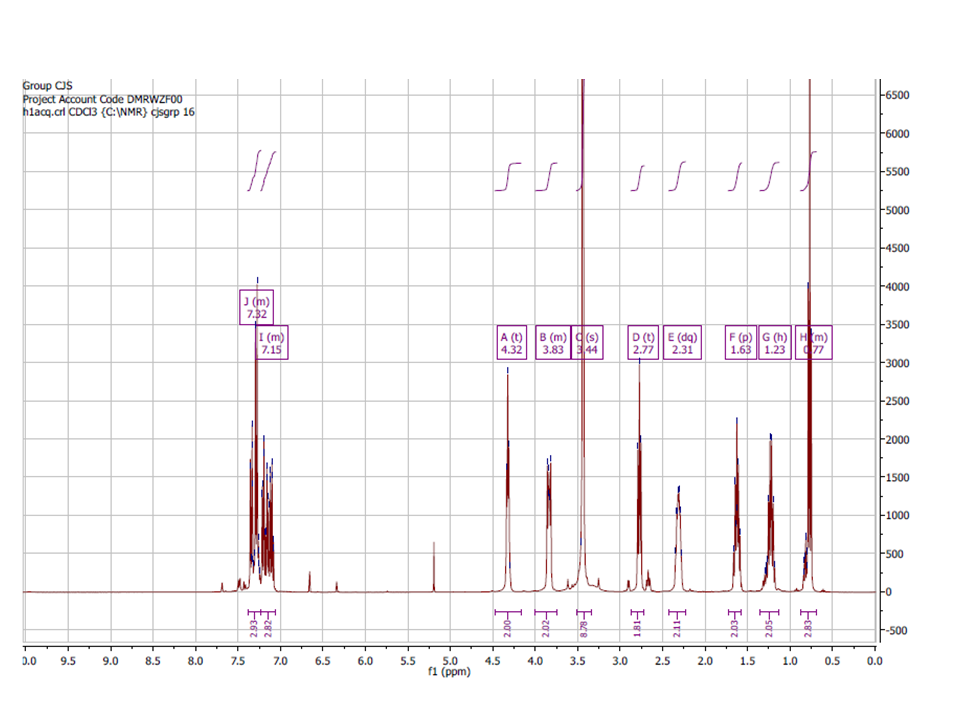


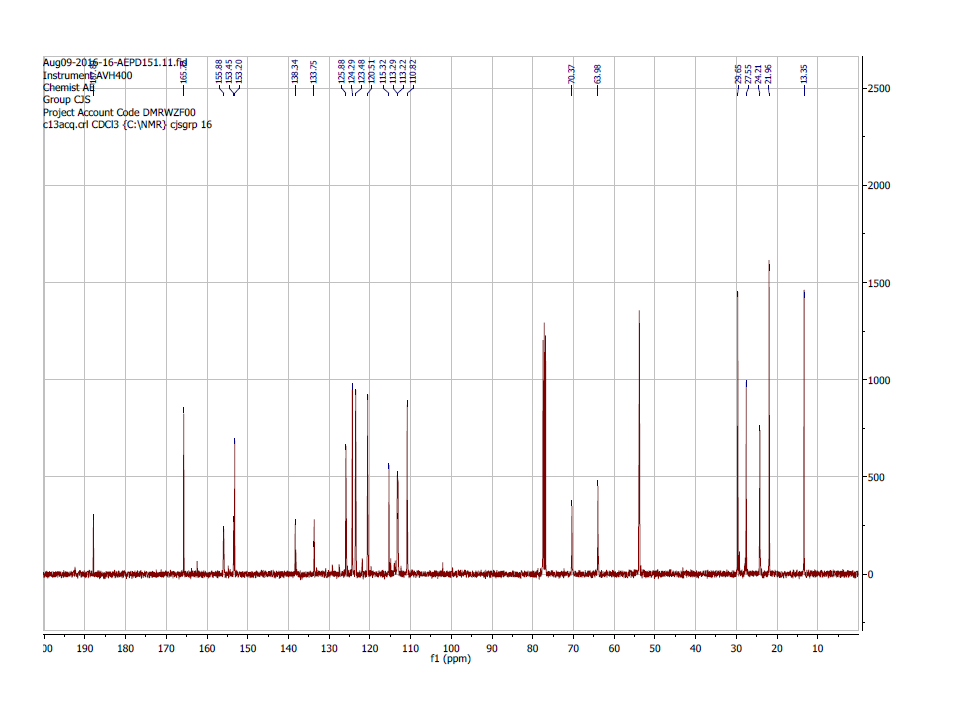


**(2-Butylbenzofuran-3-yl)(4-(3-(diethylamino)propoxy)phenyl)methanone**

At 0°C, (2-butylbenzofuran-3-yl)(4-hydroxyphenyl)methanone (200 mg; 0.68 mmol), 3-(diethylamino)propanol (0.11 mL; 0.72 mmol) and PPh_3_ (189 mg; 0.72 mmol) were dissolved in 5 mL of THF and DIAD (146 mg; 0.72 mmol) was added. The mixture was stirred at room temperature overnight. The consumption of the phenol was monitored by TLC and the reaction was diluted with 30 mL of EtOAc. The solution was washed with 30 mL of water, then brine and dried with Na_2_SO_4_. After filtration, the solvent was evaporated and purification of the crude product by flash chromatography (0 to 10 % of EtOAc in cyclohexane) gave compound **11** as a colourless oil (110 mg; 40%).

**^1^H NMR (400 MHz, CDCl_3_)** *δ*: 7.81 (2H; d; *J* = 8.0 Hz; H11,H12), 7.43 (1H; m; H4), 7.34 (1H; m; H5), 7.23 (1H; m; H3), 7.14 (1H; m; H6), 6.93 (2H; d; *J* = 8 Hz; H13,H14), 4.07 (2H; m; H17), 2.89 (2H; m; H23), 2.60 (2H; m; H18), 2.52 (4H; m; H19,H20), 1.94 (2H; m; H16), 1.73 (2H; m; H24), 1.33 (2H; m; H25), 1.01 (6H; m; H21,H22), 0.86 (3H; t; *J* = 7.0 Hz; H26).

**^13^C NMR (100 MHz, CDCl_3_)** *δ*: 190.4 (C9), 164.5 (C1), 163.0 (C15), 153.6 (C2), 131.6 (C11,C12), 128.4 (C10), 127.2 (C7), 124.1 (C4), 123.3 (C5), 121.2 (C6), 116.8 (C8), 114.1 (C13,C14), 110.9 (C3), 66.6 (C16), 49.2 (C18), 47.0 (C19,C20), 30.2 (C23), 27.8 (C24), 26.9 (C17), 22.4 (C25), 13.7 (C26), 11.8 (C21,C22).

**HRMS-ESI (m/z)**: exact mass calculated for C_26_H_34_O_3_N [M+H]^+^: 408.25332, found: 408.25297.

**IR (Diamant ATR, cm^-1^):** 2964, 2930, 2872, 2798, 1600, 1200, 1167, 881, 749.


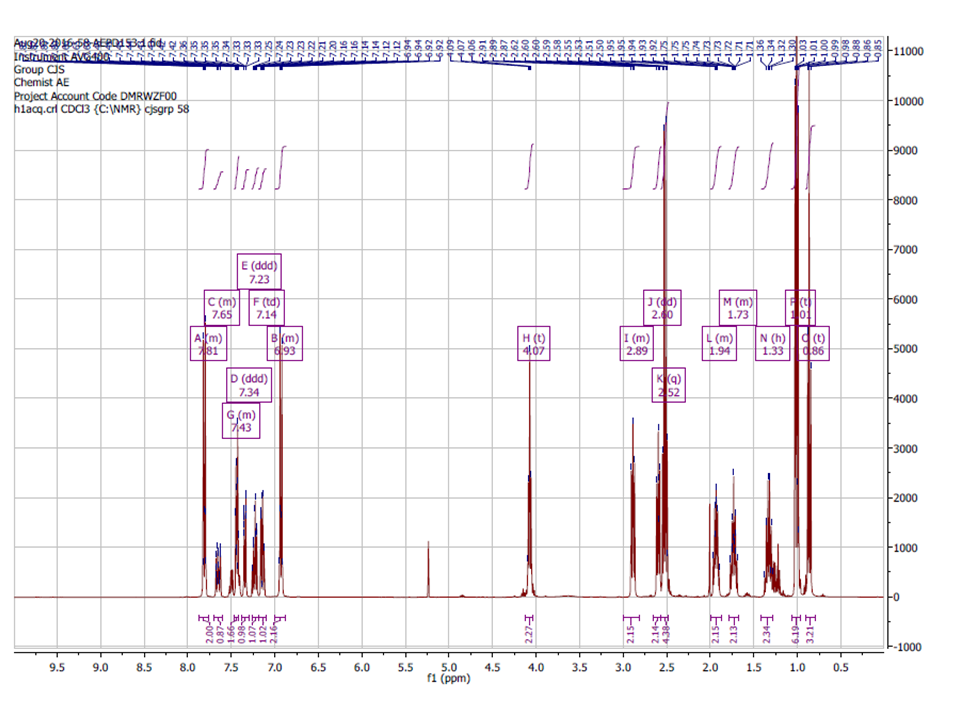


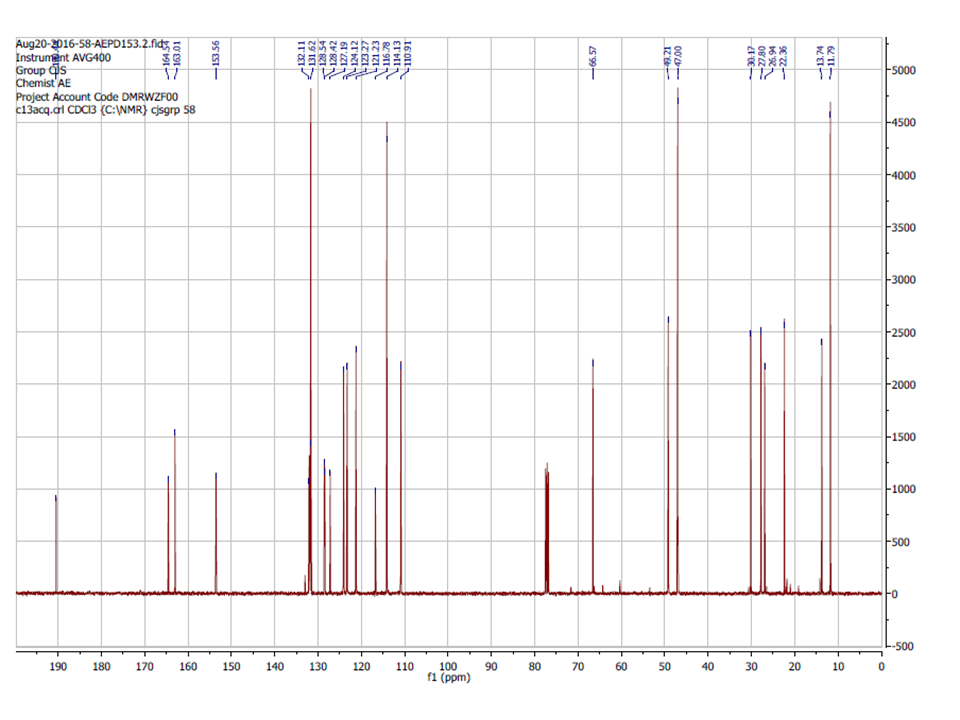


3**-(4-(2-Butylbenzofuran-3-carbonyl)phenoxy)-N,N,N-triethylpropan-1-aminium iodide**

Compound **11** (90 mg; 0.22 mmol) was dissolved in 2 mL of CH_2_Cl_2_ and iodoethane (20 µL; 0.24 mmol) and potassium carbonate (33 mg; 0.24 mmol) were added. The mixture was stirred for 2 hours at room temperature and all organic liquids were evaporated to isolate compound **12** as a white powder (93 mg; 97%).

**^1^H NMR (400 MHz, CDCl_3_)** *δ*: 7.72 (2H; m; *J* = 8.0 Hz; H11,H12), 7.40 (1H; m; H4), 7.26 (1H; m; H5), 7.19 (1H; m; H3), 7.11 (1H; m; H6), 6.89 (2H; m; *J* = 8.0 Hz; H13,H14), 4.20 (2H; m; H17), 3.55 (2H; m; H18), 3.45 (6H; q; *J* = 7.0 Hz; H19,H20,H21), 2.81 (2H; m; H25), 2.28 (2H; m; H16), 1.66 (2H; m; H26), 1.36 (9H; t; *J* = 7.0 Hz; H22,H23,H24), 1.26 (2H; m; H27), 0.81 (3H; t; *J* = 7.5 Hz; H28).

**^13^C NMR (100 MHz, CDCl_3_)** *δ*: 190.5 (C9) 164.8 (C1), 161.8 (C15), 153.4 (C2), 132.3 (C10), 131.5 (C11,C12), 126.9 (C7), 124.2 (C4), 123.3 (C5), 121.0 (C6), 116.5 (C8), 114.2 (C13,C14), 110.9 (C3), 64.5 (C16), 54.0 (C18), 53.8 (C19,C20,C21), 30.0 (C25), 27.7 (C17), 22.6 (C26), 22.2 (C27), 13.7 (C28), 8.2 (C22,C23,C24).

**HRMS-ESI (m/z)**: exact mass calculated for C_28_H_38_O_3_N [M]^+^: 436.28462, found: 436.28436.

**IR (Diamant ATR, cm^-1^):** 2959, 2930, 1641, 1203, 729.


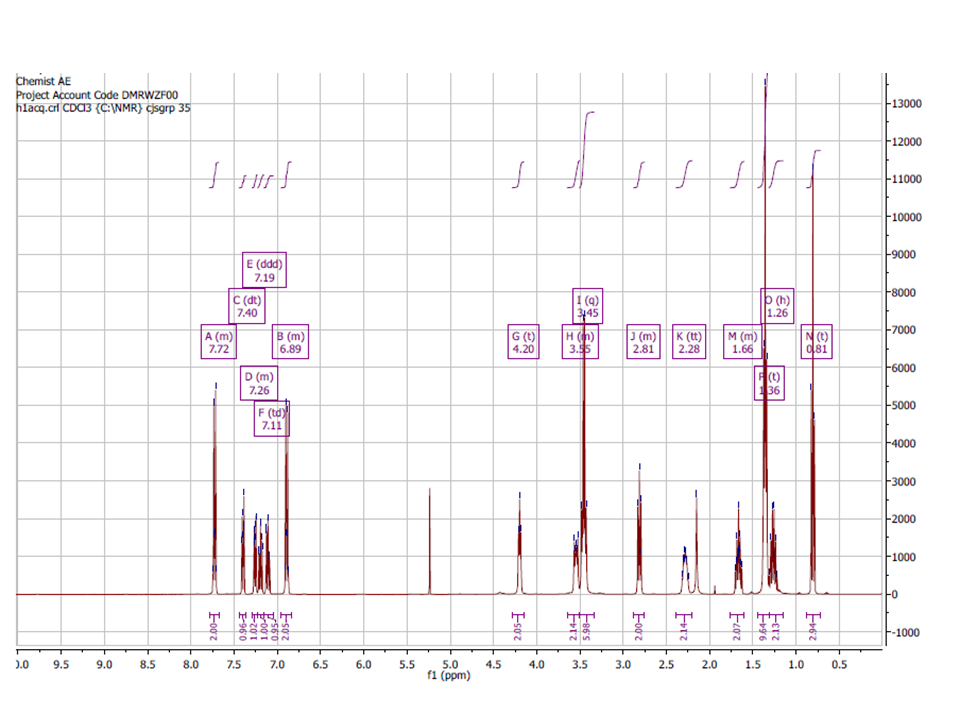


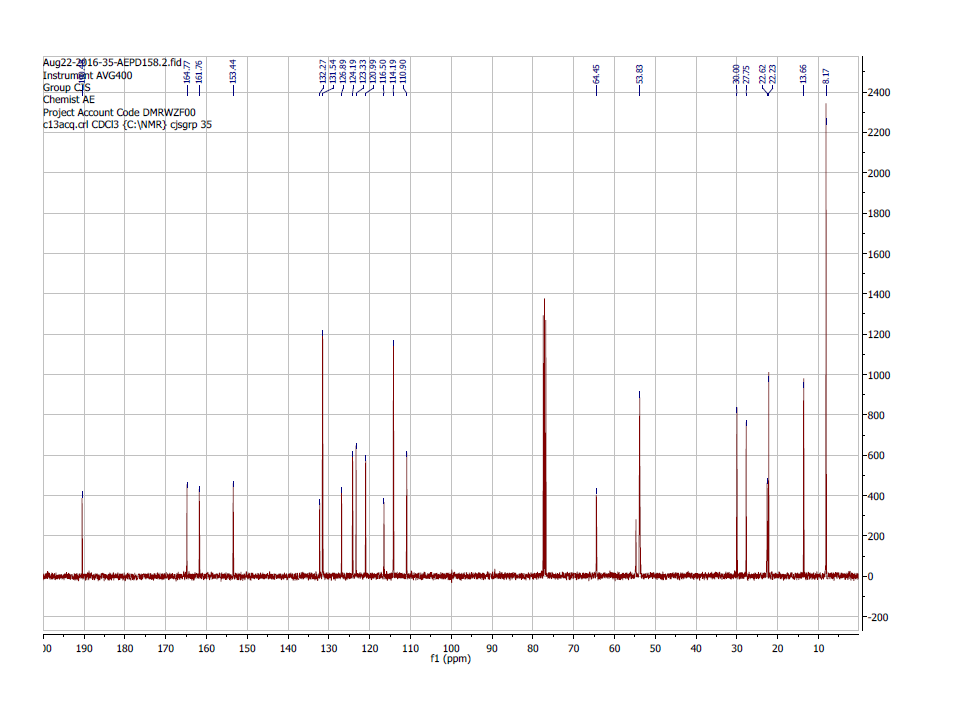


**(2-Butylbenzofuran-3-yl)(2-methoxyphenyl)methanone**

Compound **5a**’ was prepared according to general procedure C using 2-butylbenzofuran (0.26 mL; 1.47 mmol), 2-methoxybenzoyl chloride (0.22 mL; 1.47 mmol) and aluminium trichloride (385 mg; 2.94 mmol). Purification of the crude product by flash chromatography (0 to 10% of EtOAc in cyclohexane) gave a yellow oil (421 mg; 95%).

**^1^H NMR (400 MHz, CDCl_3_)** *δ*: 7.45 (4H; m; H3,H6,H13,H15), 7.25 (1H; m: H4), 7.18 (1H; m; H5), 7.06 (1H; m; H14), 6.99 (1H; m; H11), 3.69 (3H; s; H16), 2.83 (2H; m; H17), 1.70 (2H; m; H18), 1.31 (2H; m; H19), 0.88 (3H; t; *J* = 7.0 Hz; H20).

**^13^C NMR (100 MHz, CDCl_3_)** *δ*: 191.5 (C9), 167.2 (C1), 157.0 (C11), 153.6 (C2), 132.0 (C15), 131.0 (C10), 128.8 (C7), 126.6 (C12), 124.3 (C4), 123.7 (C5), 121.5 (C13), 120.8 (C6), 117.8 (C8), 111.5 (C3), 110.8 (C14), 55.6 (C16), 30.1 (C17), 28.0 (C18), 22.5 (C19), 13.7 (C20).

**HRMS-ESI (m/z)**: exact mass calculated for C_20_H_21_O_3_ [M+H]^+^: 309.14852, found: 309.14860.

**IR (Diamant ATR, cm^-1^):** 2958, 2931, 2837, 1646, 1101, 731.


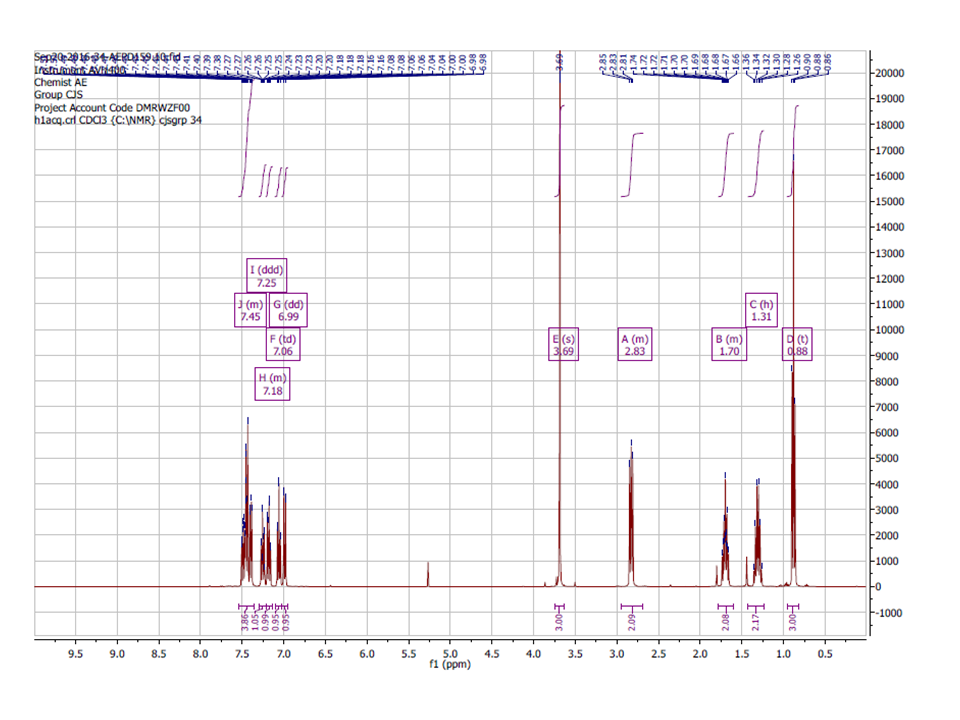


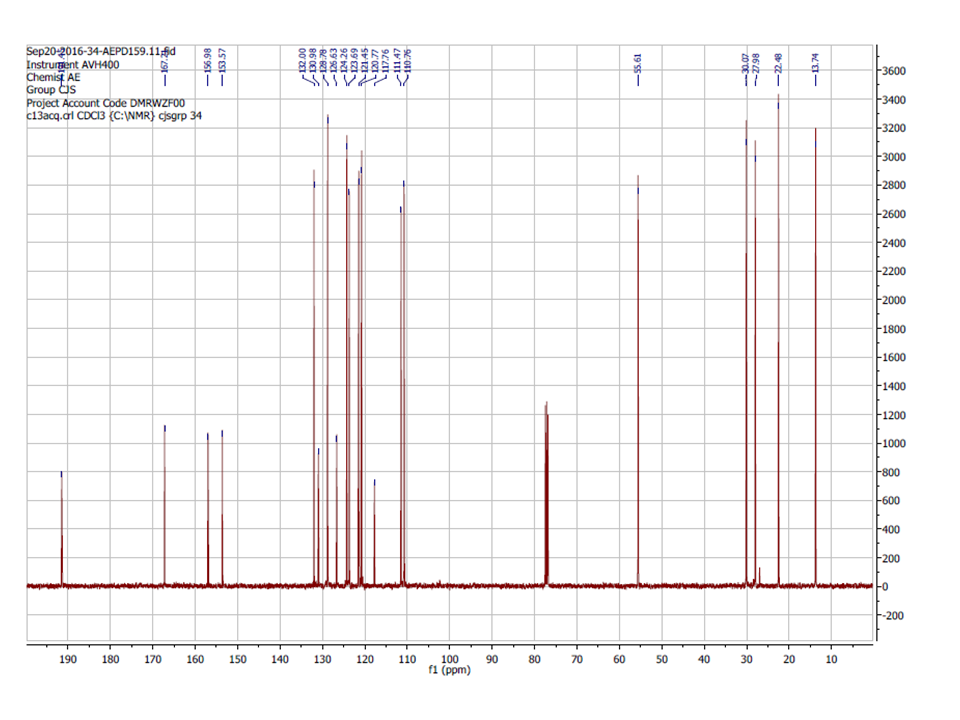


**(2-Butylbenzofuran-3-yl)(2-hydroxyphenyl)methanone**

Compound **6a′** was prepared according to general procedure D using compound **5a′** (420 mg; 1.36 mmol) and aluminium trichloride (357 mg; 2.7 mmol). Purification of the crude product by flash chromatography (0 to 20% of EtOAc in cyclohexane) gave a yellow oil (393 mg; 98%).

**^1^H NMR (400 MHz, CDCl_3_)** *δ*: 7.64 (1H; m; H3), 7.47 (2H; m; H15,H6), 7.37 (1H; m; H13), 7.26 (1H; m; H4), 7.19 (1H; m; H5), 7.05 (1H; m; H14), 6.82 (1H; m; H11), 2.88 (2H; m; H16), 1.74 (2H; m; H17), 1.34 (2H; m; H18), 0.87 (3H; t; *J* = 7.0 Hz; H19).

**^13^C NMR (100 MHz, CDCl_3_)** *δ*: 196.3 (C9), 164.0 (C1), 162.7 (C12), 153.7 (C2), 136.7 (C15), 132.9 (C11), 127.0 (C7), 124.6 (C4), 123.7 (C5), 121.0 (C14), 120.6 (C6), 118.9 (C10), 118.4 (C8), 116.4 (C13), 111.3 (C3), 30.3 (C16), 27.9 (C17), 22.5 (C18), 13.8 (C19).

**HRMS-ESI (m/z)**: exact mass calculated for C_19_H_19_O_3_ [M+H]^+^: 295.13287, found: 295.13289.

**IR (Diamant ATR, cm^-1^):** 2958, 2930, 2872, 1623, 914, 762.


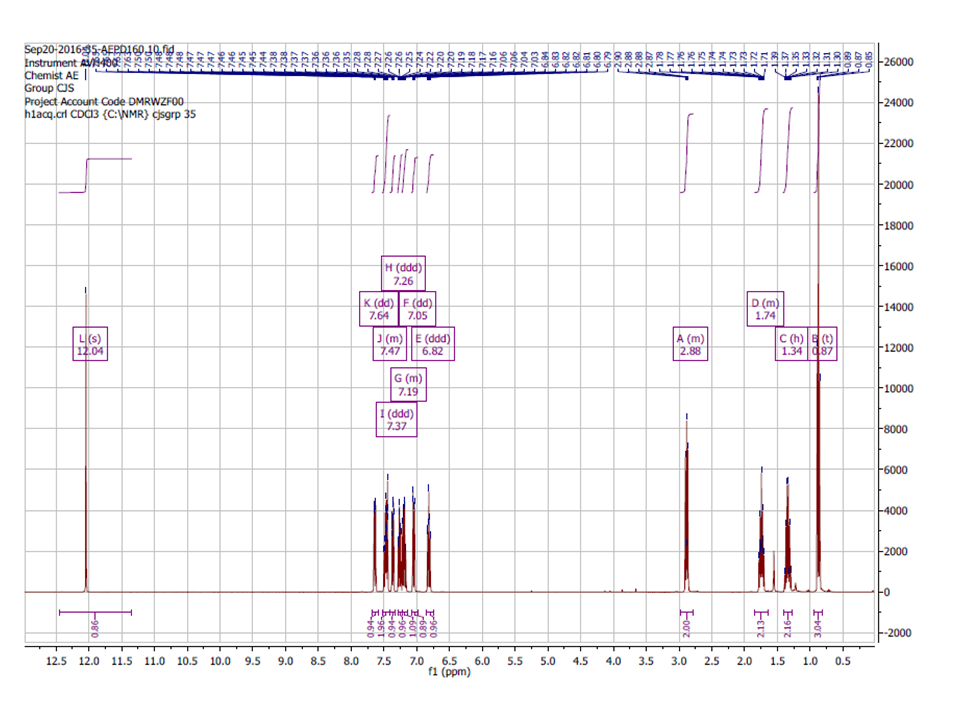


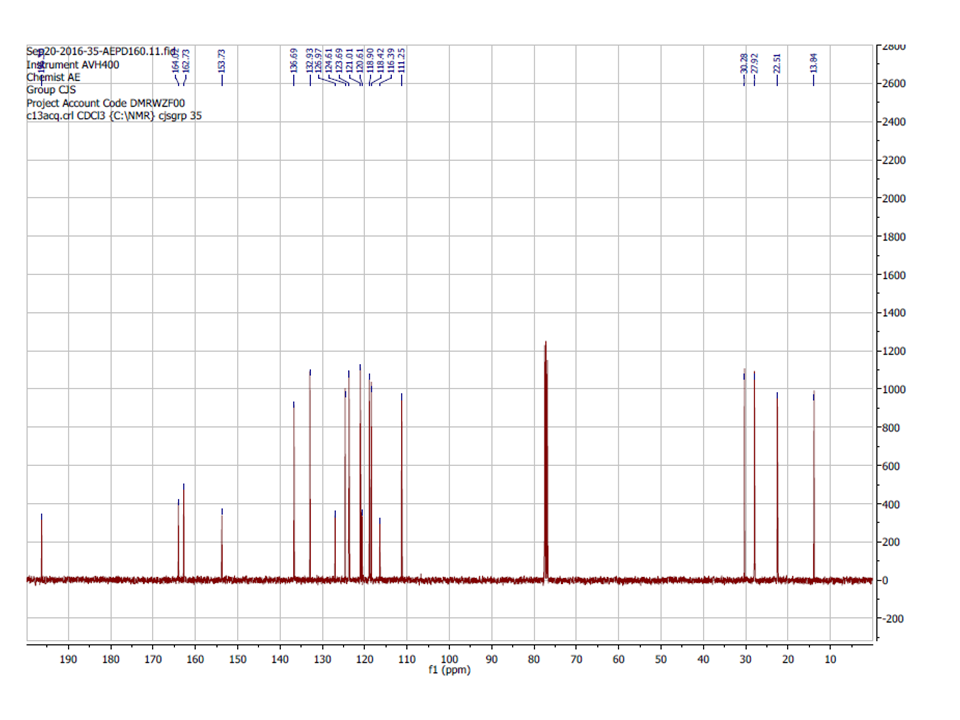


**(2-Butylbenzofuran-3-yl)(2-(3-(dimethylamino)propoxy)phenyl)methanone**

Compound **1a’** was prepared according to general procedure A using compound **6a′** (393 mg; 2.49 mmol), 3-chloro-*N*,*N*-dimethylpropan-1-amine hydrochloride (2.16 g; 13.7 mmol) and potassium carbonate (2.27 g; 16.4 mmol). Purification of the crude product by flash chromatography (0 to 15 % of MeOH in CH_2_Cl_2_) gave a yellow oil (211 mg; 42 %).

**^1^H NMR (400 MHz, CDCl_3_)** *δ*: 8.21 (1H; m; H6), 7.62 (1H; m; H15), 7.44 (1H; m; H3), 7.33 (2H; m; H13,H4), 7.13 (1H; m; H14), 6.98 (2H; m; H11,H5), 3.96 (2H; m; H17), 2.49 (2H; m; H18), 2.24 (2H; m; H21), 2.07 (6H; s; H19,H20), 1.77 (2H; m; H22), 1.63 (2H; m; H16), 1.27 (2H; m; H23), 0.81 (3H; t; *J* = 7.0 Hz; H24).

**^13^C NMR (100 MHz, CDCl_3_)** *δ*: 176.8 (C9), 166.8 (C1), 156.8 (C14), 156.1 (C2), 133.1 (C12), 131.8 (C15), 129.5 (C11), 126.2 (C7), 124.6 (C4), 123.4 (C5), 122.6 (C6), 120.7 (C10), 120.2 (C8), 117.7 (C13), 112.3 (C3), 66.6 (C16), 56.4 (C18), 45.4 (C19,C20), 32.4 (C21), 29.2 (C22), 27.6 (C17), 22.2 (C23), 13.8 (C24).

**HRMS-ESI (m/z)**: exact mass calculated for C_24_H_30_O_3_N [M+H]^+^: 380.22202, found: 380.22168.

**IR (Diamant ATR, cm^-1^):** 2957, 2930, 2859, 2764, 1641, 1387, 760.


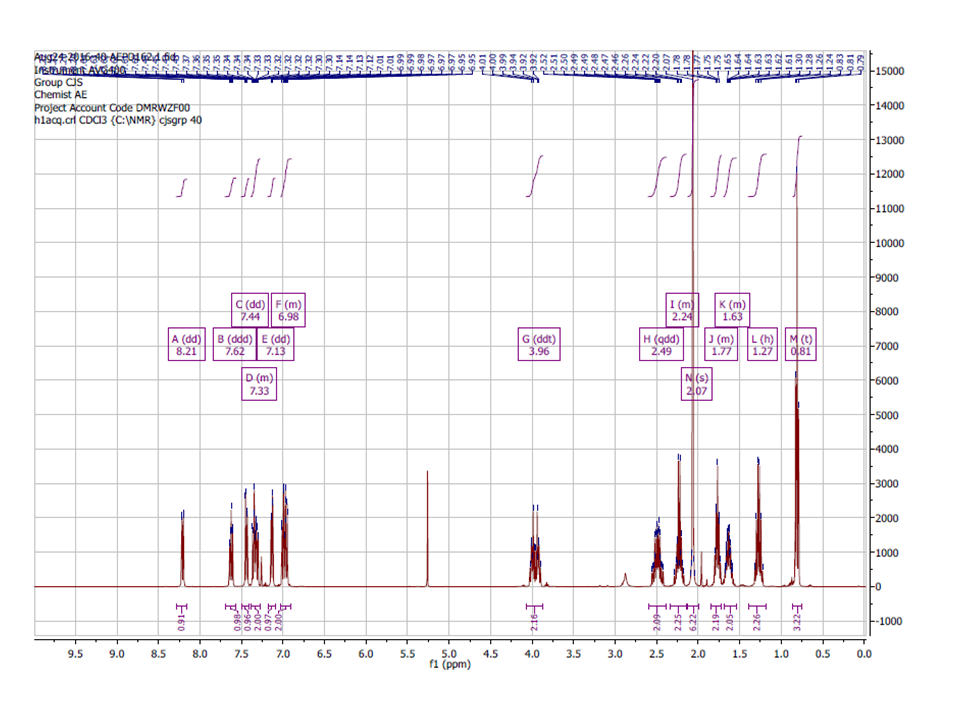


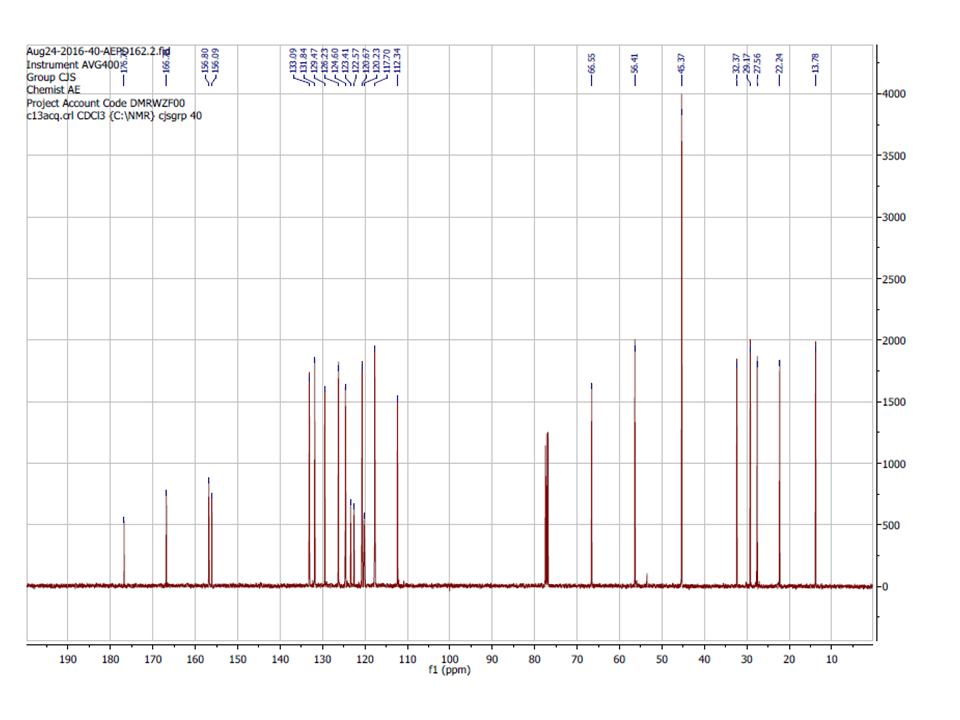

Compound **7a** was prepared according to general procedure B using compound **1a′** (100 mg; 0.26 mmol) and iodomethane (50 µL; 0.79 mmol) to give a yellow powder (120 mg; 87 %).

**^1^H NMR (400 MHz, CDCl_3_)** *δ*: 8.07 (1H; m; H3), 7.70 (1H; m; H15), 7.51 (1H; m; H6), 7.41 (1H; m; H4), 7.36 (1H; m; H13), 7.08 (1H; m; H5), 7.00 (2H; m; H11,H14), 4.27 (1H; m; H17), 4.10 (1H; m; H17), 3.59 (1H; m; H18), 3.48 (1H; m; H18), 3.20 (9H; s; H19,H20,H21), 2.46 (2H; m; H22), 2.24 (1H; m; H16), 2.03 (1H; m; H16), 1.63 (2H; m; H23), 1.26 (2H; m; H24), 0.81 (3H; t; *J* = 7.5 Hz; H25).

**^13^C NMR (100 MHz, CDCl_3_)** *δ*: 177.0 (C9), 168.0 (C1), 156.2 (C14), 155.2 (C2), 133.9 (C12), 131.9 (C15), 130.0 (C11), 125.4 (C7), 125.3 (C4), 123.0 (C5), 122.7 (C6), 121.4 (C10), 120.4 (C8), 118.3 (C13), 112.1 (C3), 63.9 (C19,C20,C21), 63.2 (C16), 53.6 (C18), 32.5 (C22), 29.4 (C23), 22.8 (C17), 22.3 (C24), 13.8 (C25).

**HRMS-ESI (m/z)**: exact mass calculated for C_25_H_32_O_3_N [M]^+^: 394.23767, found: 394.23743.

**IR (Diamant ATR, cm^-1^):** 3007, 2958, 2930, 1632, 1464, 729.


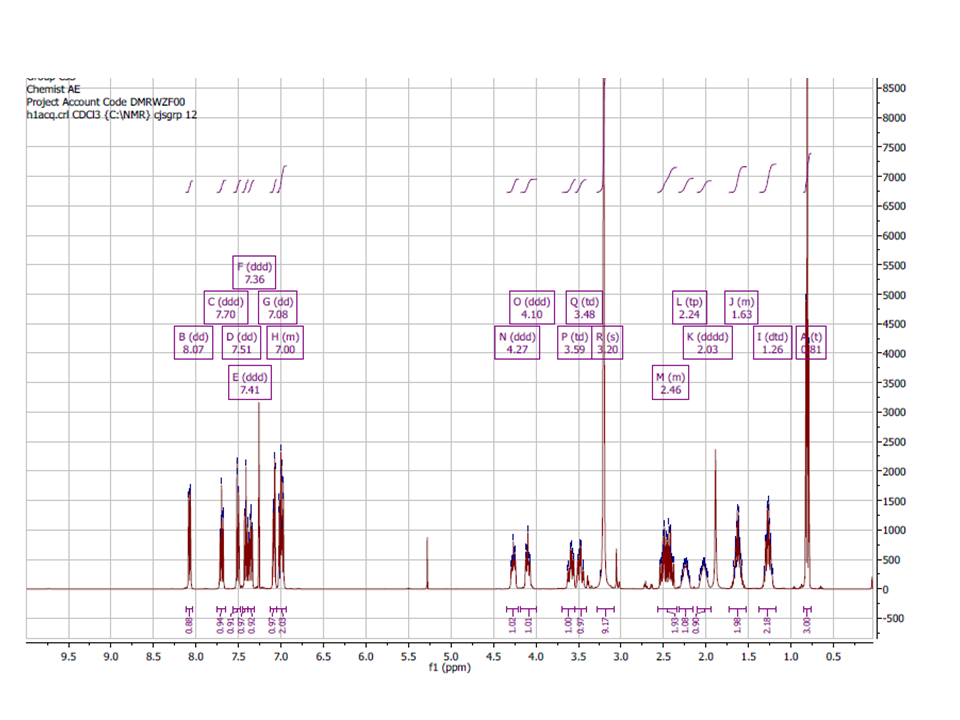


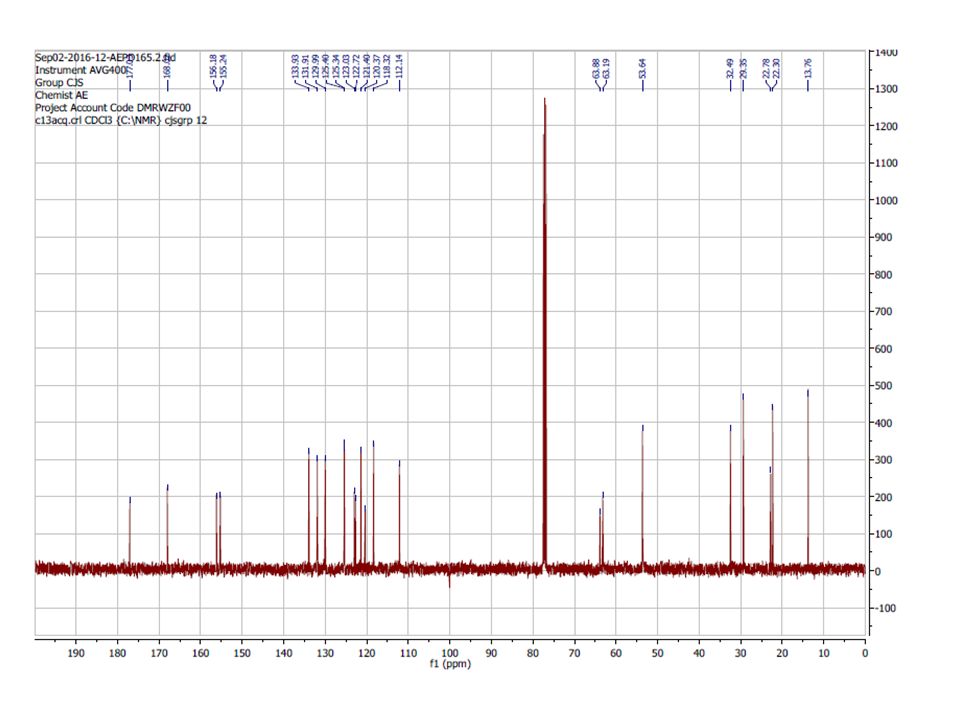

Compound **7b** was similarly prepared according to general procedure B using (2-methylbenzofuran-3-yl)(2-(3-(dimethylamino)propoxy)phenyl)methanone (200 mg; 0.6 mmol) and iodomethane (110 µL; 1.8 mmol) to give a yellow powder (30 mg; 14 %)

**^1^H NMR (400 MHz, CDCl_3_)** δ: 8.10 (1H; dd; H6), 8.06 (1H; dd; H12), 7.71 (1H; ddd; H15), 7.52 (1H; dd; H3), 7.43 (1H; ddd; H4), 7.37 (1H; ddd; H5), 7.10 (1H; dd; H14), 7.01 (1H; m; H13), 4.23(1H; ddd; H16), 4.12 (1H; ddd; H16), 3.53 (2H; m; H18), 3.19 (9H; s; H19, H20, H21), 2.25 (3H; s; H22), 2.06 (2H; m; H17)

**^13^C NMR (100 MHz, CDCl_3_)** δ: 177.0 (C9), 165.1 (C11), 162.7(C2), 156.1 (C1), 155.3 (C15), 134.1(C12), 131.9 (C4), 130.1(C7), 125.5 (C10), 125.4 (C13), 122.9 (C5), 122.6 (C8), 120.6 (C6), 118.4 (C14), 112.4 (C3), 64.1 (C16), 63.5 (C18), 53.7 (C19, 20, 21), 22.9 (C17), 19.7 (C22)

**HRMS-ESI (m/z)**: exact mass calculated for C_22_H_26_NO_3_^+^[M]^+^: 352.1910, found: 352.1906.

**IR (Diamant ATR, cm^-1^):** 2959, 2360, 2342, 1631, 1491, 763.

**References**

1 Rose, N. R. *et al.* Plant Growth Regulator Daminozide Is a Selective Inhibitor of Human KDM2/7 Histone Demethylases. *J. Med. Chem.* **55**, 6639-6643, doi:10.1021/jm300677j (2012).

2 Johansson, C. *et al.* Structural analysis of human KDM5B guides histone demethylase inhibitor development. *Nature Chem Biol,* **12**(7):539-45. doi: 10.1038/nchembio.2087 (2016).
